# Immunoproteomic Analysis Reveals Novel Candidate Antigens for the Diagnosis of Paracoccidioidomycosis Due to *Paracoccidioides lutzii*

**DOI:** 10.3390/jof6040357

**Published:** 2020-12-11

**Authors:** Anderson Messias Rodrigues, Paula Helena Kubitschek-Barreira, Breno Gonçalves Pinheiro, André Teixeira-Ferreira, Rosane Christine Hahn, Zoilo Pires de Camargo

**Affiliations:** 1 Laboratory of Emerging Fungal Pathogens, Department of Microbiology, Immunology, and Parasitology, Discipline of Cellular Biology, Federal University of São Paulo (UNIFESP), São Paulo 04023062, Brazil; brenogonpi@gmail.com; 2 Department of Cellular Biology, Roberto Alcantara Gomes Institute of Biology, Rio de Janeiro State University (UERJ), Rio de Janeiro 20511010, Brazil; paulahkb@gmail.com; 3 Toxinology Laboratory, Department of Physiology and Pharmacodynamics, Fiocruz, Rio de Janeiro 21040900, Brazil; atsferreira@gmail.com; 4 Laboratory of Mycology/Research, Faculty of Medicine, Federal University of Mato Grosso, Cuiabá 78060900, Brazil; rchahn@terra.com.br; 5 Júlio Muller University Hospital, Federal University of Mato Grosso, Cuiabá 78048902, Brazil; 6 Department of Medicine, Discipline of Infectious Diseases, Federal University of São Paulo (UNIFESP), São Paulo 04023062, Brazil

**Keywords:** immunoproteomic, proteomic, *Paracoccidioides brasiliensis*, *Paracoccidioides lutzii*, paracoccidioidomycosis, serology, diagnosis, endemic mycosis, systemic mycosis, biomarker

## Abstract

Paracoccidioidomycosis (PCM) is a life-threatening systemic infection caused by the fungal pathogen *Paracoccidioides brasiliensis* and related species. Whole-genome sequencing and stage-specific proteomic analysis of *Paracoccidioides* offer the opportunity to profile humoral immune responses against *P. lutzii* and *P. brasiliensis s. str.* infection using innovative screening approaches. Here, an immunoproteomic approach was used to identify PCM-associated antigens that elicit immune responses by combining 2-D electrophoresis of *P. lutzii* and *P. brasiliensis* proteomes, immunological detection using a gold-standard serum, and mass spectrometry analysis. A total of 16 and 25 highly immunoreactive proteins were identified in *P. lutzii* and *P. brasiliensis*, respectively, and 29 were shown to be the novel antigens for *Paracoccidioides* species, including seven uncharacterized proteins. Among the panel of proteins identified, most are involved in metabolic pathways, carbon metabolism, and biosynthesis of secondary metabolites in both immunoproteomes. Remarkably, six isoforms of the surface-associated enolase in the range of 54 kDa were identified as the major antigens in human PCM due to *P. lutzii.* These novel immunoproteomes of *Paracoccidioides* will be employed to develop a sensitive and affordable point-of-care diagnostic assay and an effective vaccine to identify infected hosts and prevent infection and development of human PCM. These findings provide a unique opportunity for the refinement of diagnostic tools of this important neglected systemic mycosis, which is usually associated with poverty.

## 1. Introduction

Paracoccidioidomycosis (PCM) is a life-threatening systemic infection caused by the fungal pathogen *Paracoccidioides brasiliensis* and related species. The disease was first described in Brazil in 1908 by Adolfo Lutz [1], followed shortly thereafter by reported infections in a wide area of the Americas. The genus *Paracoccidioides* was described in 1930 by Floriano de Almeida [2], and currently it infects at least 10 million people [3,4], being the most prevalent systemic mycosis in Latin America [5,6]. The incidence of PCM is estimated to be one to three cases per 100,000 inhabitants, and the majority of cases occur in endemic areas of Brazil, Colombia, and Venezuela [6,7]. The characteristics of the work environment can play a role, together with other risk factors, in PCM development. Therefore, PCM has been mostly reported in male rural workers during the most productive years of their lives, which poses a significant public health problem and causes substantial economic losses. Mortality associated with PCM varies between 6.1% [8] and 7.6% [9], ranking it as the eighth most common cause of death among infectious and parasitic chronic diseases [10].

Patients become infected after inhaling *Paracoccidioides* propagules from the environment, leading to primary pulmonary infection in the vast majority of human cases. In the host tissue, *Paracoccidioides* undergoes a thermodimorphic transition at 37 °C and develops as a multibudding yeast [11]. The temperature-induced switching of *Paracoccidioides* species from a saprophytic filamentous morphotype in the soil at 25 °C to a pathogenic budding yeast form in the human host is an essential morphological adaptation shared with other dimorphic pathogens in the Onygenales. While the disease is classically characterized by pulmonary involvement and systemic infections, lesions in the cutaneous and subcutaneous tissues with regional lymphocutaneous dissemination may also occur [12,13,14].

The criteria for laboratory diagnosis of PCM rely on positive microscopic identification of the causative agent in KOH preparations or biopsy specimens, where it is possible to visualize large yeasts (usually 5–15 µm) that have a thick, birefringent cell wall with single or multiple buds in a “steering wheel” or “Mickey Mouse” shape and are considered pathognomonic in the diagnosis of PCM [15]. The reference method for the definitive diagnosis is the isolation of the fungus in vitro from clinical material such as sputum or tissue fragments [15]. Even though classical techniques offer essential information due to the presence of pathognomonic forms of *Paracoccidioides* spp., nucleic acid-based diagnostic techniques are gradually replacing or complementing culture-based and biochemical assays in the routine of microbiology laboratories [16].

Serological assays are an essential aid in the clinical laboratory, as they provide a presumptive diagnosis and prognosis of the disease and represent an important tool for PCM patients’ follow-up during treatment [17,18,19,20]. Serological tests can be directed towards detecting circulating antibodies or circulating antigens in various biological fluids, such as serum, bronchoalveolar lavage, cerebrospinal fluid, sputum, etc. We highlight the routine use of double immunodiffusion (DID), which allows the quantitative detection of circulating antibodies in PCM [21,22]. Serological assays also include techniques such as counterimmunoelectrophoresis reaction (CIE) [23], enzyme-linked immunosorbent assay (ELISA) [24], latex agglutination assay [25], and immunoblotting [26], which are available from different reference services in Brazil [27]. These tests usually employ crude preparations of the glycoprotein of 43 kDa (GP43) as the primary antigen of *P. brasiliensis sensu lato* (*s.l.*) to detect circulating antibodies and have sensitivity between 85% and 100% [27]. Concerning serology tests, *P. lutzii*, for instance, has a striking antigenic variation, and since most serologic tests were developed with *P. brasiliensis s.l.* antigens, *P. lutzii*-infected hosts may have false-negative results [22].

Early DNA sequencing analyses in the 2000s demonstrated considerable diversity and recognized cryptic entities in *Paracoccidioides* [28,29,30,31]. Therefore, this classification has been revisited several times [32,33,34]. Presently, it is well known that there are at least four cryptic species included in the *P. brasiliensis* complex [35,36,37] and a distantly related group named *P. lutzii* [35]. There is a recent proposal to elevate agents in the *P. brasiliensis* complex to species level, where *P. brasiliensis sensu stricto* (*s. str.*) is represented by the S1 group (clusters S1a and S1b), and the remaining phylogenetic species are called *P. americana* (PS2), *P. restrepiensis* (PS3), and *P. venezuelensis* (PS4) [28,36,37]. The paroxysm of the conflicting organization of the *Paracoccidioides* genus is the fact that so far, name change relies only on molecular characteristics and usually does not follow phenotypic dissimilarities or the clinical pictures of the patients infected with the plurality of *Paracoccidioides* species. Although such taxonomic modifications may be excessive and could cause a great deal of confusion, there is still a need to revisit the taxonomy using modern approaches such as polyphasic taxonomy [38] or “consilient taxonomy” [39,40]. The availability of sequenced genomes [41,42] enables a new opportunity to consider the *Paracoccidioides* taxonomy in harmony with other phenotypic, epidemiological, and clinical attributes.

Useful epidemiological surveys are available for *Paracoccidioides* and reveal that the *P. brasiliensis* complex members occur in sympatry in a vast South American area. Phylogenetic species S1 (S1a and S1b) are the most common PCM agents and are widespread throughout South America, especially in southeastern and southern Brazil, Argentina, and Paraguay. A few cases of the PS2 group have been reported in Venezuela and southeast Brazil. PS3 and PS4 are rare agents of PCM, and isolated cases have been found in Colombia and Venezuela, respectively. Lately, cases related to PS3 have begun to emerge in Colombia, Argentina, Peru, and Brazil [42,43,44]. The offshoot *P. lutzii* encompasses a single species. The epicenter of *P. lutzii* mycosis is in the Midwest and Amazon regions of Brazil, with a single case reported in Ecuador [45,46].

The finding of other species of *Paracoccidioides* opens new fields for research on the centenarian PCM. Unlike the traditional *P. brasiliensis*, the newly described *P. lutzii* remains enigmatic in many aspects [37,47]. Our group recently initiated the serological study of PCM due to *P. lutzii,* thus contributing to PCM differentiation by the *P. brasiliensis* complex and *P. lutzii* [22]. However, little is known about the antigenic molecules recognized by *P. brasiliensis*/*P. lutzii*-PCM patient sera, except for the gp43 of *P. brasiliensis*, which is recognized by all *P. brasiliensis*-PCM sera [48]. Moreover, no studies have compared the immunogenic antigens recognized between PCM caused by *P. brasiliensis s. str.* and *P. lutzii* in humans.

**Figure 1:**
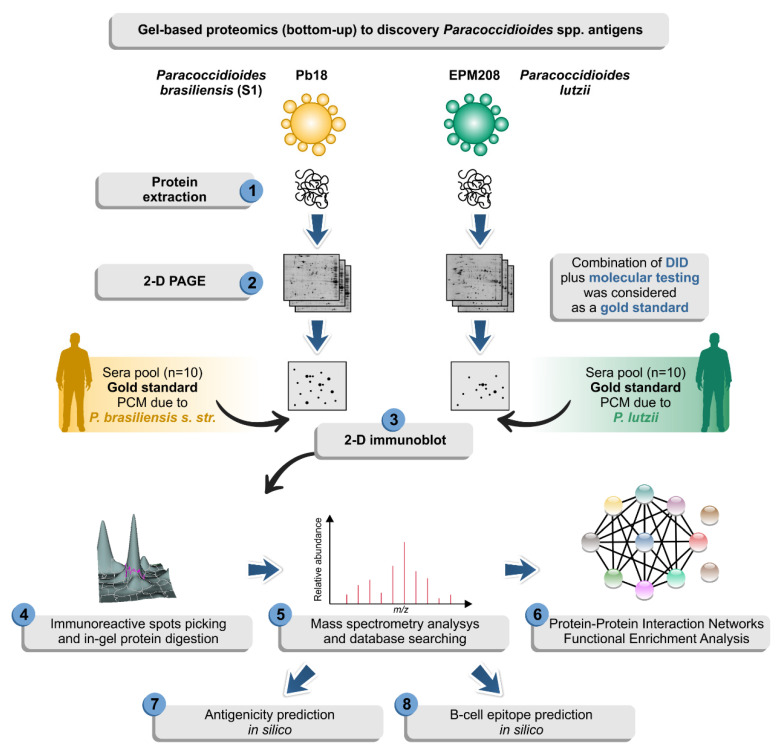
Diagram depicting a two-dimensional (2-D) proteomic approach followed by 2-D immunoblot used in this study in an attempt to identify possible antigens of *Paracoccidioides* species. The isolate EPM208 of *P. lutzii* was selected based on previous experiments showing that patients with *P. lutzii* mycosis show 100% reactivity with antigenic preparations from this strain [22]. The classical isolate Pb18 (S1) was used for comparison in the immunoproteomics experiments [54]. Afterward, the yeasts were used to prepare protein extracts (step 1) and then resolved by 2-D gel electrophoresis (step 2). Proteomes were transferred to membranes and probed against sera from patients with confirmed paracoccidioidomycosis (PCM) due to *P. lutzii* or *P. brasiliensis* (S1) (step 3). We consider as gold standard serum, the samples that presented positive double immunodiffusion (DID) results and where it was possible to isolate the strain from the patient, following molecular characterization of this isolate. Therefore, only a paired serum: strain was used in the 2-D immunoblot. IgG-reactive proteins (step 4) were identified by mass spectrometry (step 5). Finally, a solid bioinformatic characterization of the immunoreactive protein was employed to highlight potential biomarkers for diagnosis of paracoccidioidomycosis due to *P. lutzii* (steps 6–8).

Immunoproteomics is a potentially useful tool to identify disease-associated antigens that elicit immune responses by combining protein separation (2-DE, gel-free separation), immunological detection (immunoblotting), and mass spectrometry or by combining immunocapture and mass spectrometry [49]. Therefore, immunoproteomics effectively provides general diagnostic indications for selecting biomarkers [50] and has been used to analyze several medically relevant microorganisms [51,52,53]. Here, we propose an experimental immunoproteomics design using antigen preparation derived from the yeast phase of *Paracoccidioides* species probed against a “gold standard” serum, where for the same patient, we were able to combine fungal isolates identified down to species level by molecular methods and serum of the patient typified by serology (Figure 1). We explored the potential of immunoproteomics to gain insights into the main IgG-reactive molecules in PCM caused by the two main PCM agents, including *P. lutzii* and *P. brasiliensis s. str.* Emphasis was given to the *P. lutzii* system since there is still no indication of the main antigenic molecule responsible for antigen–antibody reaction in PCM due to *P. lutzii*.

## 2. Materials and Methods

### 2.1. Paracoccidioides Strains and Molecular Characterization

The *Paracoccidioides* spp. strains used in this study were the classical EPM208 and Pb18 (EPM16) isolates. Isolates were characterized down to species level by *TUB1*-RFLP of the alpha-tubulin gene, as described earlier [55]. Attenuation of virulence can occur in some *Paracoccidioides* isolates when subjected to successive in vitro subculturing [56,57] and thus affect the proteome expressed by isolates. Therefore, to avoid any bias among *Paracoccidioides* spp. isolates at the start of in vitro culturing, isolates were passed through BALB/c and then reisolated before protein extraction, as previously described by our group [54].

### 2.2. Protein Sample Extraction

*Paracoccidioides* yeast cells were grown for 7 days at 36 °C in biological triplicate in semisolid Fava-Netto medium, and protein extraction was performed as previously described by Rodrigues et al. [58]. Briefly, yeast cells were washed in PBS, centrifuged (5000× *g*, 5 min, 4 °C), frozen in liquid nitrogen, and ground in a mortar and pestle until a fine powder was obtained. The powder was suspended in 3 mL of Tris-Ca^2+^ buffer (20 mM Tris-HCl pH 8.8, 2 mM CaCl_2_) containing a commercial cocktail of protease inhibitors (1:100) (GE Healthcare, Piscataway, NJ, USA), RNase, and DNase enzymes (1:100) (GE Healthcare, Piscataway, NJ, USA). Then, glass beads (Sigma, St. Louis, MO, USA, 425–600 µm) were added, and the mixture was vigorously vortexed for 30 min at 4 °C. Cell debris and glass beads were removed by centrifugation (11,000× *g*, 4 °C, 10 min), and dithiothreitol (20 mM) was added to the supernatant [59]. Protein concentrations were determined by the Bradford method [60], and the protein extracts were kept at −70 °C until use.

### 2.3. Two-Dimensional Gel Electrophoresis

Proteins (300 µg) from each replicate were precipitated using the 2D Clean-Up Kit (GE Healthcare, Piscataway, NJ, USA) following the manufacturer’s recommendations. Proteins were diluted with rehydration solution (7 M urea, 2 M thiourea, 2% CHAPS, 1.2% DeStreak, 2% vol/vol isoelectric focusing (IEF) buffer pH 4–7, and trace bromophenol blue) to a final volume of 250 µL. IEF was performed using an Ettan IPGphor III system (GE Healthcare, USA). Precast IPG strips (pH 4–7, 13 cm) were rehydrated at 30 V for 12 h. Proteins were focused at 200 V for 2 h, 500 V for 2 h, 1000 V for 5 h, and then a gradient was applied from 1000 to 5000 V for 2 h. Finally, the voltage was set to 5000 V until 60,000 Vhr. All IEF experiments were performed at 20 °C. Prior to running the second dimension, the IPG strips were reduced for 15 min with 1.5% dithioerythritol and alkylated for 15 min with 2.5% iodoacetamide in equilibration buffer (6 M urea, 50 mM Tris–HCl pH 6.8, 30% glycerol, and 2% SDS). Equilibrated strips were placed on homogeneous 10% polyacrylamide gels (16 cm × 16 cm, GE Healthcare, Piscataway, NJ, USA) and sealed with 0.5% low-melting-point agarose and separated at 10 °C using a Hoefer SE 600 unit (15 mA/gel for 30 min and then 23 mA/gel until the dye front reached the bottom of the gel) [58]. Proteins were either developed with silver [61] and Coomassie staining [62] or directly transferred in the case of immunoblot analysis.

### 2.4. Naturally Infected Human Serum Samples

A total of 20 sera from patients with confirmed PCM and 10 self-reported healthy normal donors were used in this investigation. Patients had the chronic form of PCM and exhibited clinical and laboratory signs of the disease, with pulmonary system involvement and mucosal or mucocutaneous lesions [22]. PCM was confirmed in all patients by laboratory demonstration of pathognomonic “ship’s wheel” budding yeast cells and fungal isolation in vitro. Therefore, only paired samples (strain: serum) were used as gold standard. Serum samples from patients with PCM due to *P. lutzii* (*n* = 10) were recovered from the serum bank of the Laboratory of Mycology at the Federal University of Mato Grosso (Cuiabá, Midwest region, Brazil) in 2012–2015, whereas samples from patients with PCM due to *P. brasiliensis s. str.* (S1; *n* = 10) were retrieved from the serum bank of the Laboratory of Medical and Molecular Mycology at the Federal University of São Paulo (São Paulo state, Southeast region, Brazil) in 2013–2015. Samples were pooled to maximize the recognition of immunoreactive spots and avoid patient-specific effects. The pooled positive reference serum was generated by pooling equal volumes of anti–*P. lutzii* or anti-*P. brasiliensis* IgG antibody-positive sera from 10 patients, and the pooled negative reference serum was generated with sera from 10 healthy adult individuals. All serum samples were aliquoted and stored at −70 °C until use. This study was approved (CAAE: 17177613.6.0000.5541) by the research ethics committee of Federal University of Mato Grosso, and protocol numbers 1796–10 and CEP 3147220120 were approve by the counterpart committee of Federal University of São Paulo. All adult subjects provided informed written consent, and the study was also approved under number 288.250/CEP/HUJM/UFMT.

### 2.5. Double Immunodiffusion Assays

All individual sera were investigated via double immunodiffusion assays (DID) using a *P. lutzii* cell-free antigen (CFA) preparation derived from the reference isolate EPM208 or the exoantigens from the reference B-339 strain (AgPbB339; PS3) as described earlier [21,22]. A quantitative DID assay was used to establish the titration of each patient’s serum. Briefly, 3 mm of melted 1% agarose (Sigma A-6877) in PBS was poured onto a glass slide (75 mm × 25 mm) [22]. This micro-ID test pattern consisted of a central well surrounded by six wells, each 3 mm in diameter. The central well, located 6 mm (edge-to-edge) from the other wells, was filled with the antigen solution. Each slide contained two sets of wells. On each slide, the two central wells were filled with 10 µL of *Paracoccidioides* antigen. Slides were incubated overnight in a moist chamber at room temperature (20–25 °C) and then washed for 1 h in 5% sodium citrate and 24–48 h in saline. The slides were dried, stained for 5 min with 0.15% Coomassie Brilliant blue (Sigma) in ethanol∶ acetic acid∶ water (4∶2∶4; v∶v), and destained in the solvent mixture alone until precipitin lines were maximally visible. Precipitation bands were noted by visual observation [21].

### 2.6. 2-D Immunoblot of Paracoccidioides Species Proteins

For immunoblotting, proteins separated by conventional 2-D gel electrophoresis were electrophoretically transferred onto 0.45 μm polyvinylidene difluoride (PVDF) membranes (Bio-Rad, USA) at 25 V for 1 h with transfer buffer (25 mM Tris base, 192 mM glycine, 20% methanol; pH 8.3) [63] using a Trans-Blot SD semidry transfer cell (Bio-Rad, Rockville, MD, USA). The success of electrotransfer was evaluated by Ponceau S staining (0.15% Ponceau S and 1% acetic acid (v/v)) staining. Membranes were destained and then free binding sites were blocked overnight in PBS blocking buffer (1% bovine serum albumin, supplemented with 0.05% (v/v) Tween 20 and 5% (wt/vol) skim milk, pH 7.6) at 4 °C [64].

Membranes were probed with primary antibody diluted 1:500 (gold standard pooled human sera) at 25 °C for 2 h. Afterward, the membranes were washed three times with Tris-buffered saline (pH 7.5) containing 0.05% (v/v) Tween 20 (TBST) for 10 min and incubated with horseradish peroxidase (HRP)-conjugated goat anti-human IgG (1:1000 dilution) for 2 h at room temperature. The membranes were then washed with TBST, and the signal was detected with an enhanced chemiluminescence detection kit (GE Healthcare, Uppsala, Sweden). Immunoblots were imaged in a transilluminator (Uvitec, Cambridge). Alliance software v. 4.7 was used to capture several images at different exposure times, reaching 10 shots with a 2 s increment.

### 2.7. Identification of Immunoreactive Proteins by MALDI-ToF MS/MS Analysis

Fresh Coomassie-stained protein spots matching 2-D immunoblot immunoreactive spots were manually excised and processed by MALDI-ToF/MS. Spots were washed in ultrapure water, destained in 50 mM ammonium bicarbonate (pH 8.0) with 50% acetonitrile (ACN), shrunk with 100% acetonitrile, and vacuum dried. Next, the gel pieces were incubated with 12.5 ng/µL of sequencing grade trypsin (Promega, Madison, WI, USA) overnight at 37 °C. After trypsin digestion, the supernatants were separated, and the peptides extracted first into 0.5% trifluoroacetic acid/50% acetonitrile and then into 100% ACN. All extracts were pooled, and the volume was reduced by SpeedVac [58].

Following trypsin digestion, the peptide suspension derived for each spot was spotted on a MALDI target plate, mixed with the matrix α-cyano-4-hydroxy-trans-cinnamic acid (Sigma), and allowed to air dry at room temperature. The samples were analyzed with an AB SCIEX TOF/TOF 5800 mass spectrometer (Applied Biosystems, Foster City, CA, USA) in automated mode. Initially, a MALDI MS spectrum was acquired for each spot (800 shots/spectrum), and peaks with a signal-to-noise ratio greater than 50 in at least two consecutive fractions until a maximum of 10 precursors per fraction were automatically selected for MS/MS analysis (at least 2200 shots/spectrum) using collision energy of 1 keV with air as the collision gas. All mass spectra were calibrated using the Mass Standards Kit for Calibration of AB SCIEX TOF/TOF (Applied Biosystems, Foster City, CA, USA) [58]. The spectra were searched against an in-house database constructed with genome information using two reference strains of *P. brasiliensis s.l.* (Pb03, accession number ABHV00000000; Pb18, accession number ABKI00000000) and one strain of *P. lutzii* (Pb01; accession number ABKH00000000) [65] as selection criteria and the Paragon algorithm in the Protein Pilot software (Applied Biosystems, Foster City, CA, USA). A false discovery rate of 1% was applied, and only peptides with >95% confidence were listed.

### 2.8. Bioinformatics Analysis

The Search Tool for the Retrieval of Interacting Genes/Proteins (STRING) (version 11) [66] was used to construct a protein interaction network (experimentally verified as well as predicted) of the immunoreactive proteins in the *P. brasiliensis* and *P. lutzii* immunoproteomes. Since most biological information is available for *Paracoccidioides brasiliensis*, it was used as a model organism in both analyses. In addition, the Kyoto Encyclopedia of Genes and Genomes (KEGG) pathway analysis [67], implemented in the STRING database, was used to ascertain the function of the identified proteins. The web application iPath3.0 was used to visualize and analyze cellular pathways enriched in STRING analysis (KEGG pathway analysis) [68]. WoLF PSORT was used for predicting subcellular protein localization of the immunoreactive proteins based on their amino acid sequences [69]. The InteractiVenn software (unions by list) [66] was used to generate Venn diagrams for our list of immunoreactive proteins, as well as to compare the dataset found in this study with those immunoreactive proteins published for human PCM [70] and murine PCM [71].

High-throughput in silico prediction of protein antigenicity was used to compare B-cell epitopes between *P. brasiliensis* (Pb18) and *P. lutzii* (Pb01) immunoreactive proteins. Protein sequences were retrieved from NCBI and submitted to ABCpred [72], BepiPred [73], and COBEpro [74] using the default parameters. AntigenPRO [74] and VaxiJen v2.0 [75], two sequence-based, alignment-free and pathogen-independent predictors of protein antigenicity, were used to predict full-length protein antigenicity with a prediction threshold of 0.5. Therefore, proteins having antigenic scores >0.5 were considered to be antigenic.

Potential biomarkers for PCM immunodiagnostics were submitted to the ConSurf server [76] to estimate the evolutionary conservation of amino acid residue positions based on the phylogenetic relations between homologous sequences. Multiple sequence alignment was achieved using MAFFT [77], and the homologs were collected from UNIREF90 [78] using the homolog search algorithm HMMER (HMMER E-value: 0.0001, number of HMMER iterations: 1, maximal %ID between sequences: 95; minimal %ID for homologs: 35) [79]. The degree to which an amino acid position is evolutionarily conserved (i.e., its evolutionary rate) is highly dependent on its structural and functional importance and was compared with predicted B-cell epitopes. The continuous conservation scores were partitioned into a discrete scale of 9 bins for visualization, where bin 9 contained the most conserved positions, and bin 1 had the most variable positions [76].

Finally, for modeling the protein’s 3D molecular structures of *Paracoccidioides* antigens, the amino acid sequences were retrieved from NCBI and submitted to RaptorX [80], a web server for protein structure–function prediction. Protein 3D molecular structures, including molecular surface and inspection of molecular structures, were determined using the 3D Molecule Viewer implemented in the CLC Genomics Workbench v.9.0.1. In the last step of the homology modeling, the model’s refined structure was subjected to a series of tests to check its internal consistency and reliability. Backbone conformation was evaluated by examining the Psi/Phi Ramachandran plot obtained from PROCHECK analysis [81]. We considered a model to have good quality when over 90% of the amino acid residues were located in the most favored regions [81].

## 3. Results

### 3.1. Protein Profile of Paracoccidioides Species

**Figure 2:**
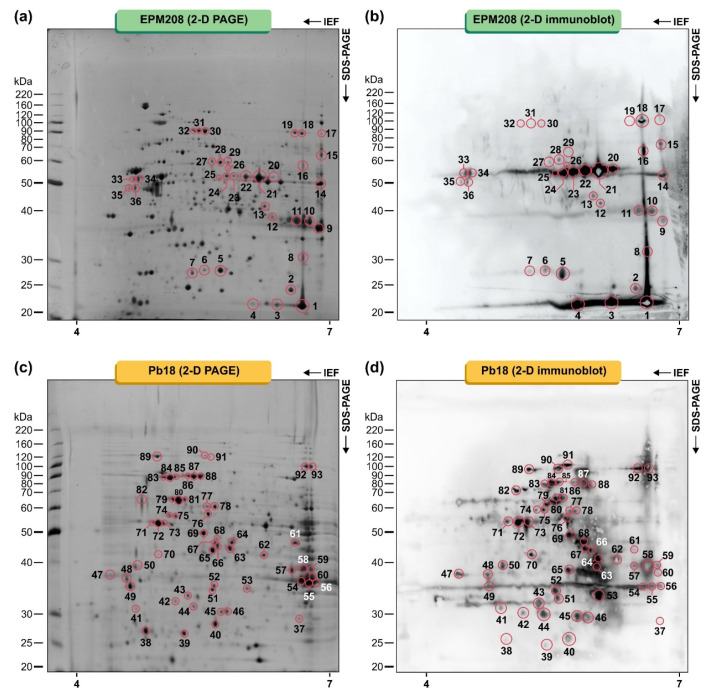
Proteins from *Paracoccidioides lutzii* (EPM208) and *Paracoccidioides brasiliensis* (Pb18) were fractionated using 13 cm pH 4–7 (left to right) strips in the first dimension and 10% SDS-PAGE gels in the second dimension, developed by silver staining. (**a**) 2-D PAGE of EPM208, (**b**) 2-D immunoblot using a pool of sera of patients (*n* = 10) with PCM due to *P. lutzii*, (**c**) 2-D PAGE of Pb18, and (**d**) 2-D immunoblot using a serum pool of patients (*n* = 10) with PCM due to *P. brasiliensis s. str.* (S1). Data are representative of three independent experiments. Molecular masses of standard proteins are given on the gels’ left side (BenchMark Protein Ladder, Invitrogen, Waltham, MA, USA). The spots recognized by sera from patients with PCM and submitted to MALDI-ToF mass spectrometry analysis (MS/MS) are highlighted. The numbers refer to the spot identification used in Table 1 and Table 2.

Protein extraction using whole yeast cells yielded around 3 mg of proteins and the proteins integrity was observed by 1D SDS-PAGE. Whole-cell lysates (300 µg) from *P. lutzii* and *P. brasiliensis* were fractionated by 2-D electrophoresis, using an immobilized pH gradient of 4–7, which provided better sample separation and improved resolution (Figure 2a,c).

### 3.2. Profiling the Humoral Response in Human PCM

All sera used in this study were typified by immunodiffusion using the AgPbB339 reference exoantigen or EPM208 cell-free antigen, as previously described [22]. Sera of patients from São Paulo were reactive to the classical antigen AgPbB339, with titration varying between 1:4 and 1:64 (Supplementary Figure S1a). The molecular identification of the isolates by *TUB1*-RFLP revealed they belong to the S1 group (*P. brasiliensis s. str.*). On the other hand, a positive reaction was detected for sera from patients living in the Brazilian Midwest using the EPM208 CFA preparation, with titration varying between 1:4 and 1:32 (Supplementary Figure S1b), showing a strong identity with *P. lutzii*. Likewise, the isolates from Mato Grosso were classified as *P. lutzii* by *TUB1*-RFLP, confirming the serological data. Sera from the healthy normal donors did not react with AgPbB339 or EPM208 CFA preparation (Supplementary Figure S1).

Afterward, typified sera were used in two-dimensional immunoblot tests, revealing a total of 93 IgG-reactive spots in the proteome of *Paracoccidioides* species (Figure 2). Thirty-six spots with molecular weights (MW) ranging from 23 to 74 kDa and with isoelectric points in the range from 5.08 to 9.02 were strongly detected in the *P. lutzii* proteome using the pooled human serum of patients with PCM due to *P. lutzii* (gold standard; Figure 2b). The immunoproteome of *P. brasiliensis* was much more diverse than that of *P. lutzii,* and 57 spots with molecular weights (MW) ranging from 16.9 to 94.6 kDa and with isoelectric points in the range from 4.68 to 7.67 were strongly detected in the pooled human serum of patients with PCM due to *P. brasiliensis s. str.* (gold standard; Figure 2d). Moreover, no significant IgG binding reactivity was detected with the sera from healthy subjects.

### 3.3. IgG-Reactive Protein Identification by Mass Spectrometry

**Figure 3:**
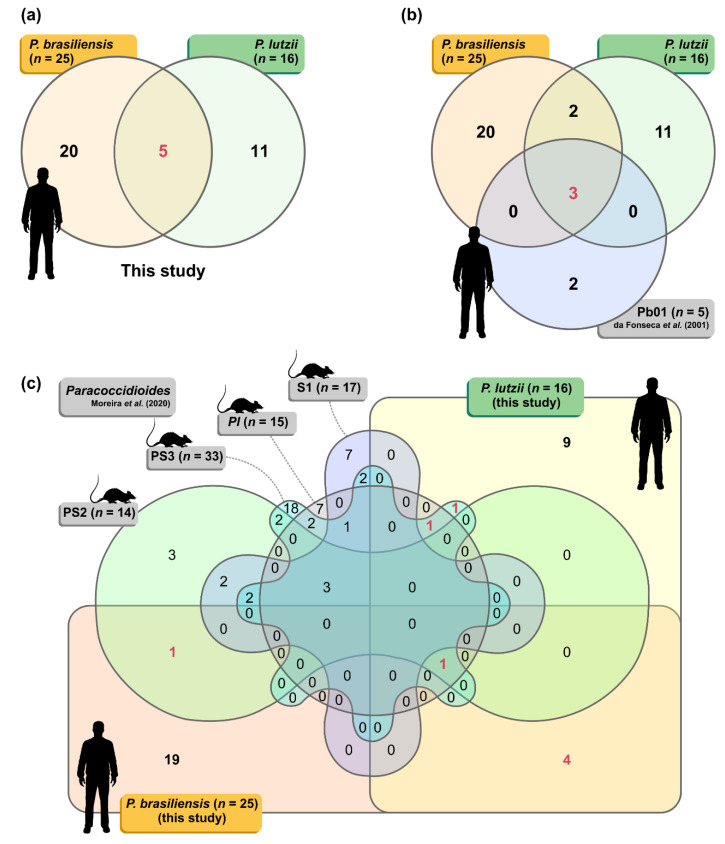
(**a**) The Venn diagram summarizes the number of proteins identified in the *P. lutzii* (*n* = 16) and *P. brasiliensis* (*n* = 25) proteomes. (**b**) The three-way Venn diagram summarizes the number of proteins in each combination found in this study (*P. lutzii* and *P. brasiliensis*) shared with the immunoproteome published by da Fonseca et al. [70] for human PCM (*n* = 5 immunoreactive proteins). (**c**) The six-way Venn diagram summarizes the number of proteins in each combination found in this study (*P. lutzii* and *P. brasiliensis*) shared with the immunoproteomes published by Moreira et al. [71] for murine PCM using *P. brasiliensis s. str.* (S1; *n* = 17 immunoreactive proteins), *P. americana* (PS2; *n* = 14 immunoreactive proteins), *P. restrepiensis* (PS3; *n* = 33 immunoreactive proteins), and *P. lutzii* (*n* = 15 immunoreactive proteins). Shared proteins are highlighted in red.

A total of 93 spots matching the immunoreactive proteins described above were excised from fresh 2-D Coomassie-stained gels and submitted to mass spectrometry analysis (MS/MS). Homology database search with the Mascot search engine allowed identifying 77 immunoreactive spots corresponding to 16 proteins in the *P. lutzii* proteome (Table 1) and 25 proteins in the *P. brasiliensis* proteome (Table 2). Interestingly, several spots showed two or more isoforms, such as NAD(P)H:quinone oxidoreductase, type IV (spots # Pl01, Pl03, and Pl04); glyceraldehyde-3-phosphate dehydrogenase (spots # Pl09 and Pl10), HSP72-like protein (spots # Pl13, Pl30, Pl31, and Pl32), transketolase (spots # Pl17 and Pl19), and enolase (spots # Pl20, Pl21, Pl22, Pl23, Pl24 and Pl34). Three uncharacterized proteins were detected for the first time in the *P. lutzii* immunoproteome and four were detected for the first time in the *P. brasiliensis* immunoproteome. Surprisingly, we did not identify any isoforms of the glycoprotein of 43,000 Da (gp43) in the immunoproteome of *P. lutzii* or *P. brasiliensis*. However, this molecule has been reported as the immunodominant antigen in PCM patients infected with members of the *P. brasiliensis* complex. Only five proteins were found to be shared between *P. lutzii* and *P. brasiliensis* immunoproteomes (i.e., triosephosphate isomerase, glyceraldehyde-3-phosphate dehydrogenase, fructose-bisphosphate aldolase 1, HSP72-like protein, and HSP60, mitochondrial) (Figure 3a), and three of them were previously described in PCM (Figure 3b). A comparison between our dataset and previous immunogenic proteins reported for murine PCM revealed a remarkable difference between human and murine immunogenic proteins. Only four proteins were found to be common between the systems (Figure 3c).

### 3.4. STRING Analysis of Paracoccidioides Immunoproteome

**Figure 4:**
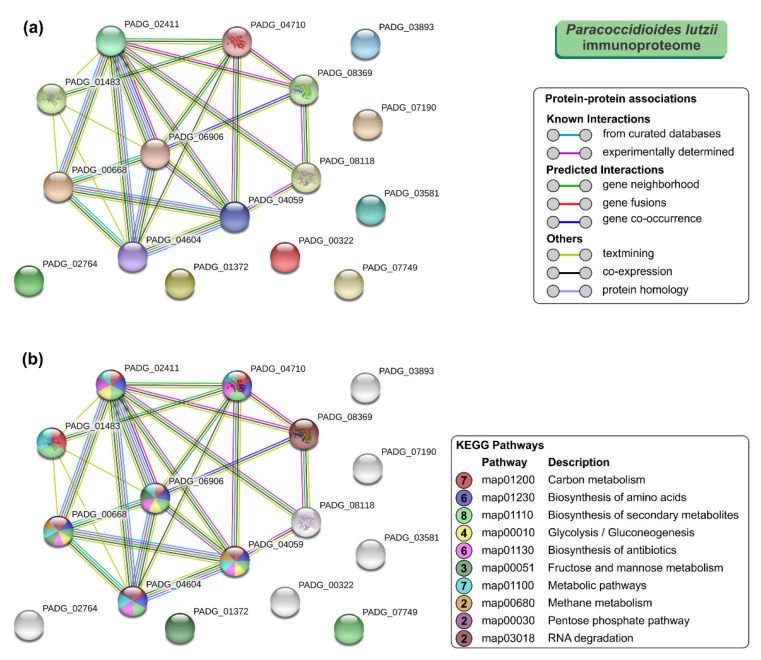
The Search Tool for the Retrieval of Interacting Genes/Proteins (STRING) analysis of *Paracoccidioides lutzii* immunoproteome. (**a**) A total of 16 immunoreactive proteins from *P. lutzii* were used as input for STRING analysis, and a network was built based on high confidence evidence from experimental protein–protein interaction (purple line) and curated (light blue lines) databases. Proteins are indicated by nodes labeled with the encoding gene symbol (number of edges = 25) from *P. brasiliensis* as a model. (**b**) Classification of proteins based on the Kyoto Encyclopedia of Genes and Genomes (KEGG) pathways. Protein–protein interactions’ enrichment *p*-value = 4.33 × 10^−15^.

To further understand the properties and correlation of immunogenic proteins, the 16 antigens of *P. lutzii* were used to generate a protein–protein interaction network (*p* = 4.33 × 10^−15^; average node degree: 3.12; Figure 4a) and visualize the most representative classes of these proteins for KEGG pathways (depicted in Figure 4b). The *P. lutzii* immunogenic proteins represented a broad range of biological functions, including biosynthesis of secondary metabolites (*n* = 8; FDR = 2.29 × 10^−7^; map01110), carbon metabolism (*n* = 7; FDR = 8.22 × 10^−9^; map01200), metabolic pathways (*n* = 7; FDR = 0.00054; map01100), biosynthesis of amino acids (*n* = 6; FDR = 2.29 × 10^−7^; map01230), biosynthesis of antibiotics (*n* = 6; FDR = 6.96 × 10^−6^; map01130), glycolysis/gluconeogenesis (*n* = 4; FDR = 2.95 × 10^−6^; map00010), fructose and mannose metabolism (*n* = 3; FDR = 4.02 × 10^−5^; map00051), methane metabolism (*n* = 2; FDR = 0.0018; map00680), pentose phosphate pathway (*n* = 2; FDR = 0.0021; map00030), glyoxylate and dicarboxylate metabolism (*n* = 2; FDR = 0.0037; map00630), and RNA degradation (*n* = 2; FDR = 0.0068; map03018) (Figure 4b). The protein–protein interaction network showed that seven proteins that are mainly involved in carbon metabolism, biosynthesis of secondary metabolites, and metabolic pathways could interact with each other. The three uncharacterized proteins (XP_015700719, XP_015701261, and XP_002797075) are described as newly identified antigens, and along with NAD(P)H:quinone oxidoreductase, type IV, phenylacetone monooxygenase, proteasome subunit alpha, and mannitol-1-phosphate 5-dehydrogenase showed no relationship with others (Figure 4).

**Figure 5:**
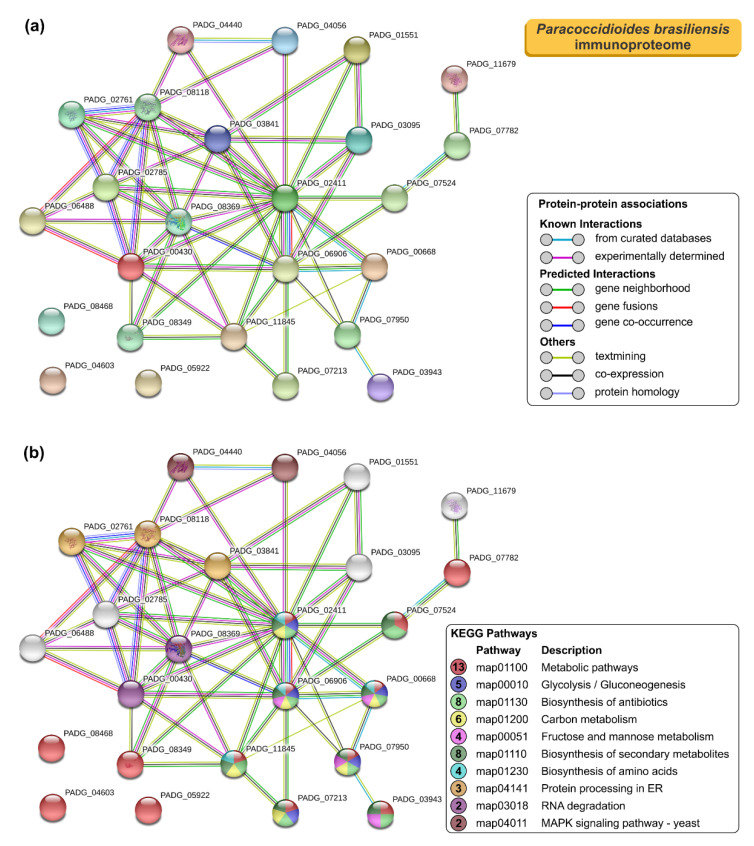
STRING analysis of *Paracoccidioides brasiliensis* immunoproteome. (**a**) A total of 25 immunoreactive proteins from *P. brasiliensis* were used as input for STRING analysis, and a network was built based on high confidence evidence from experimental protein–protein interaction (purple line) and curated (light blue lines) databases. Proteins are indicated by nodes labeled with the encoding gene symbol (number of edges = 25) from *P. brasiliensis* as a model. (**b**) Classification of proteins based on KEGG pathways. Protein–protein interactions’ enrichment *p*-value < 1.0 × 10^−16^.

As we expected, the protein–protein interaction network in STRING for the 25 immunoreactive proteins of *P. brasiliensis* had higher node degrees (*p* < 1.0 × 10^−16^; average node degree: 4.72; Figure 5a). The most representative classes of these proteins for KEGG pathways are shown in Figure 5b. Likewise, the *P. brasiliensis* immunogenic proteins covered an extensive range of biological functions, including metabolic pathways (*n* = 13; FDR = 9.58 × 10^−7^; map01100), biosynthesis of antibiotics (*n* = 8; FDR = 1.56 × 10^−6^; map01130), biosynthesis of secondary metabolites (*n* = 8; FDR = 8.33 × 10^−6^; map01110), carbon metabolism (*n* = 6; FDR = 3.94 × 10^−6^; map01200), glycolysis/gluconeogenesis (*n* = 5; FDR = 1.34 × 10^−6^; map00010), fructose and mannose metabolism (*n* = 4; FDR = 5.72 × 10^−6^; map00051), biosynthesis of amino acids (*n* = 4; FDR = 0.0013; map01230), protein processing in endoplasmic reticulum (*n* = 3; FDR = 0.0060; map04141), RNA degradation (*n* = 2; FDR = 0.0262; map03018), and MAPK signaling pathway—yeast (*n* = 2; FDR = 0.0311; map04011) (Figure 5b). The protein–protein interaction network showed that 22 proteins, mainly involved in metabolic pathways, biosynthesis of secondary metabolites, biosynthesis of antibiotics, and carbon metabolism, could interact with each other. Two uncharacterized proteins (XP_010761900 and XP_010758132), described here as newly identified antigens, interacted with each other as well as with proteins related to protein processing in the endoplasmic reticulum and RNA degradation. A newly identified 1-Cys peroxiredoxin (PbPrx1, XP_010758730) and an uncharacterized protein (XP_010759842) showed interactions with proteins related to the protein processing in the endoplasmic reticulum (e.g., HSP72-like protein and protein disulfide-isomerase). Three proteins, named, spermidine synthase, 4-hydroxyphenylpyruvate dioxygenase, and an uncharacterized protein (XP_010761260, M20_dimer domain-containing protein) showed no relationship with the others (Figure 5).

### 3.5. Immunoinformatic Analysis of Paracoccidioides Antigens

Bioinformatic analyses were used to predict subcellular localization of immunoreactive proteins. Results showed 20 cytoplasmic (55.6%), followed by 13 mitochondrial (36.1%), two nuclear (5.6%), and one extracellular localization (2.7%) (Table 3).

For the 16 *P. lutzii* immunogenic proteins, predicted antigenicity varied from 0.3002 to 0.9448 using AntigenPRO and from 0.4058 to 0.9471 using VaxiJen. Therefore, citrate synthase was not predicted to be an antigen using both predictors. In contrast, VaxiJen failed to predict another four proteins, namely, isocitrate lyase (0.4291), NAD(P)H: quinone oxidoreductase type IV (0.4315), fructose-bisphosphate aldolase 1 (0.4472), and a hypothetical protein (XP_002797075; 0.4142) using a cutoff level of 0.5. Similar predictions were found for the homolog sequences of *P. brasiliensis* (Table 3).

Judging from the 25 *P. brasiliensis* immunogenic proteins, predicted antigenicity varied from 0.2767 to 0.9382 using AntigenPRO and from 0.3264 to 0.95 using VaxiJen. Therefore, ATP synthase subunit beta (0.2767), hexokinase (0.3815), and spermidine synthase (0.429) were not predicted to be antigens using AntigenPRO, and deoxyuridine 5′-triphosphate nucleotidohydrolase (0.3264), spermidine synthase (0.4429), fructose-bisphosphate aldolase 1 (0.4869), and 4-hydroxyphenylpyruvate dioxygenase (0.4948) showed scores below 0.5. Similar predictions were found for the homolog’s sequences in *P. lutzii* (Table 3).

**Figure 6:**
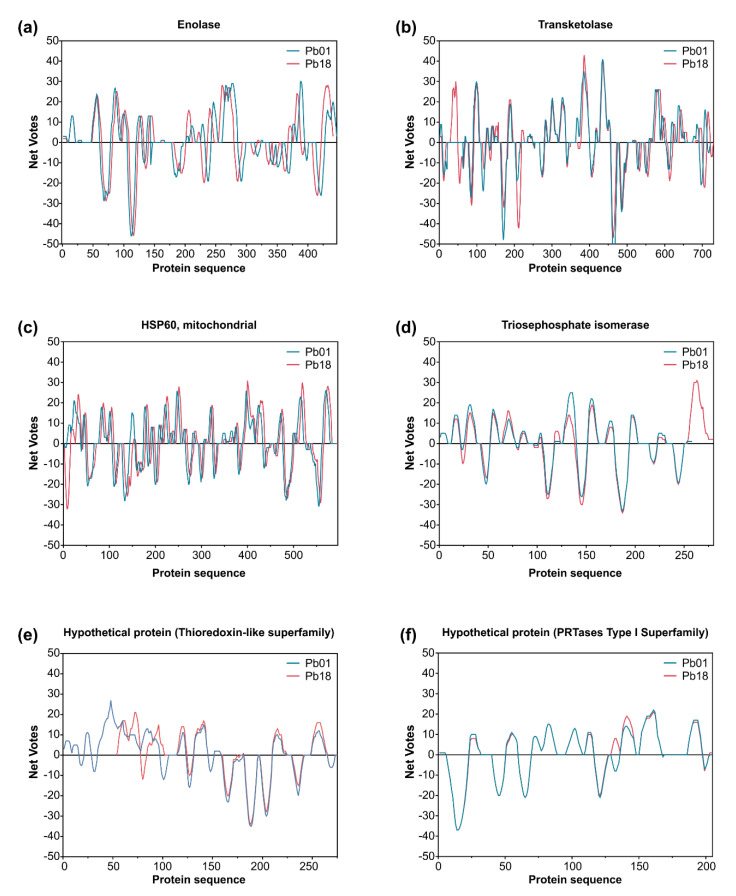
Immunoreactive proteins found in the *P. lutzii* immunoproteome with the highest antigenic scores. Residue epitope propensity score predictions for (**a**) enolase (XP_015703472 and XP_010759701), (**b**) transketolase (XP_002793534 and XP_010760067), (**c**) heat shock protein 60, mitochondrial (XP_002789992 and XP_010763632), (**d**) triosephosphate isomerase (XP_002795879 and XP_010762134), (**e**) uncharacterized protein—Thioredoxin-like superfamily (XP_015700719 and XP_010758121), and (**f**) uncharacterized protein—PRTases type I superfamily (XP_015701261 and XP_010756299). The antigenic propensity scores are plotted against position along the amino acid sequence as predicted by the COBEpro algorithm [82]. The higher the antigenic propensity scores, the more likely is the antigenic activity for the respective region. COBEpro uses a support vector machine to assign epitope propensity scores to peptide fragments.

**Figure 7:**
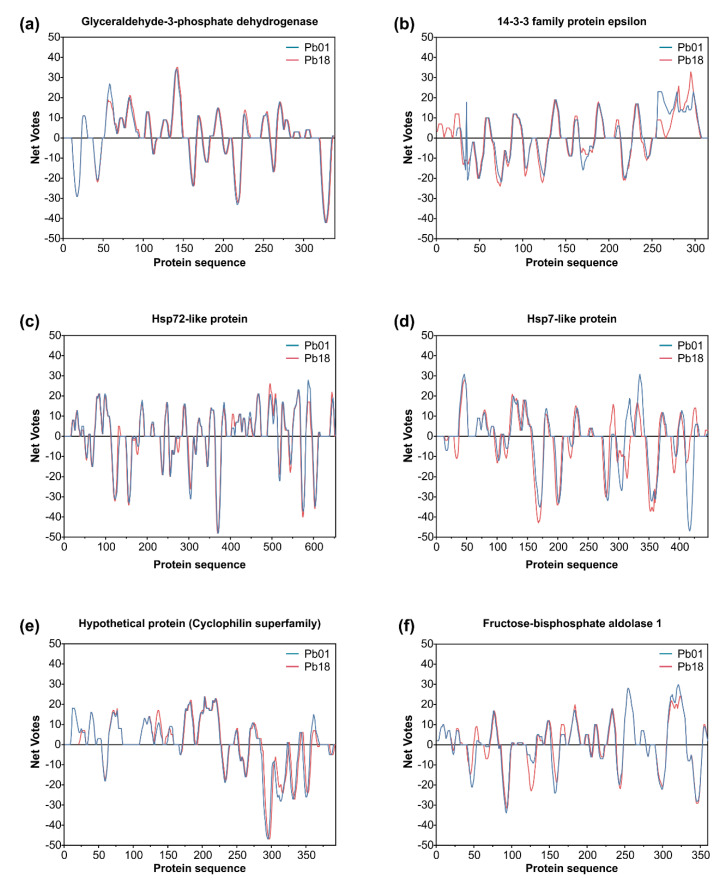
Immunoreactive proteins found in the *P. brasiliensis* immunoproteome with the highest antigenic scores. Residue epitope propensity score predictions for (**a**) glyceraldehyde-3-phosphate dehydrogenase (Q8 × 1 × 3 and XP_010758444), (**b**) 14-3-3 family protein epsilon (XP_002796914 and XP_010759301), (**c**) HSP72-like protein (XP_002790117 and XP_010763478), (**d**) HSP7-like protein (XP_015703361 and XP_010756347), (**e**) uncharacterized protein—cyclophilin superfamily (XP_015701903 and XP_010761900), and (**f**) fructose-bisphosphate aldolase 1 (XP_002796107 and XP_010756450). The antigenic propensity scores are plotted against position along the amino acid sequence as predicted by the COBEpro algorithm [82]. The higher the antigenic propensity scores, the more likely is the antigenic activity for the respective region. COBEpro uses a support vector machine to assign epitope propensity scores to peptide fragments.

The characterization of epitopes that invoke substantial responses from B-cells is critical in designing useful diagnostic assays, since B-cell epitopes can recognize definite antibodies, thus allowing diagnosis with more specificity. Based on the list of IgG-reactive proteins, epitopes were predicted from the whole protein sequence found in *P. lutzii* and its homolog in *P. brasiliensis* (and vice versa). We aimed to describe the candidates for the design of new serological assays and highlight potential vaccine targets for future studies. Our analyses revealed 219 epitopes for *P. lutzii* and 379 epitopes for *P. brasiliensis* (Supplementary Table S1). Most of the predicted immunogenic epitopes are accessible on the protein surface, located in flexible regions, and often overlap between *P. lutzii* and *P. brasiliensis*, as depicted in Figure 6 and Figure 7, reaching a total of 183 common epitopes. Therefore, although the antigenicity value was found to be somewhat similar between homologs and although the epitope regions were predicted to occur in the same region (Figure 6 and Figure 7), there were substitutions of one or more amino acid residues along with epitopes, which could dramatically decrease the antigen–antibody interaction between the *P. lutzii* or *P. brasiliensis* epitopes and the antibodies in heterologous systems. Table 3 shows the number of predicted epitopes and the number of epitopes shared between the antigenic proteins found in 2-D immunoblotting and its homolog in the sister species, indicating they have a chance of cross-reaction with other cryptic *Paracoccidioides* species causing similar infections. In this scenario, five proteins, namely, 14-3-3 family protein epsilon (Figure 7b), ATP synthase subunit beta, aconitate hydratase, mitochondrial HSP75-like protein, and an uncharacterized protein (M20_dimer domain-containing protein), appeared with the highest number of shared epitopes.

**Figure 8:**
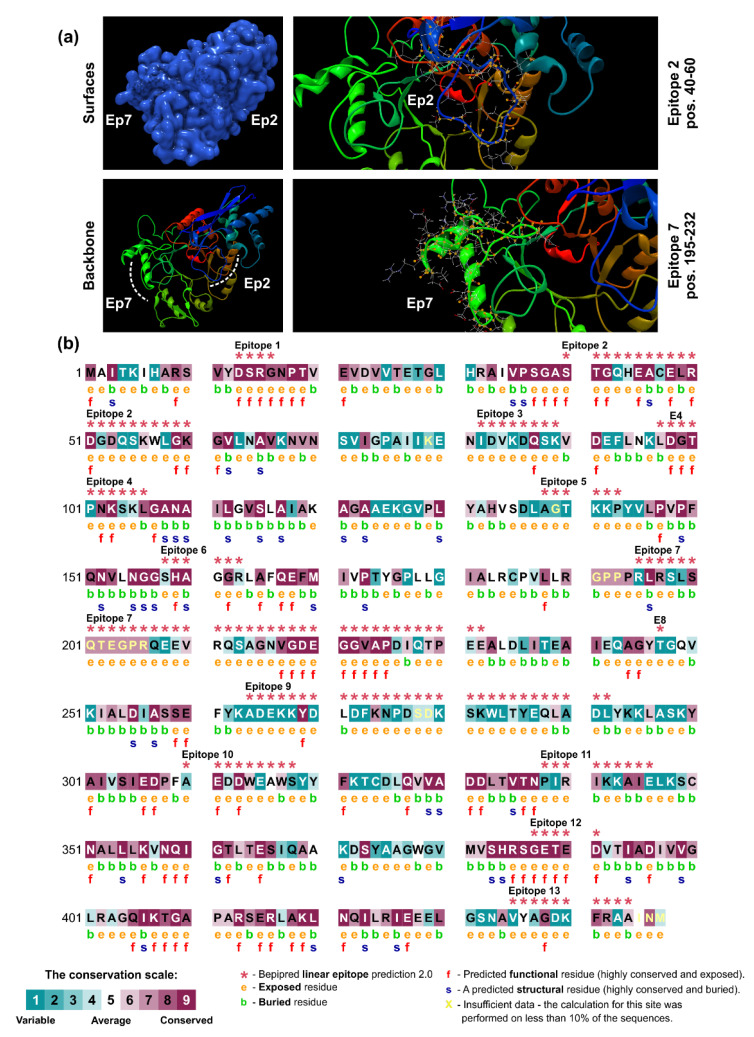
B-cell epitopes are exposed in the *P. lutzii* enolase. A comparative model for enolase was obtained from the Protein Data Bank (PDB; http://www.rcsb.org/pdb/) webserver [84], and the *Saccharomyces cerevisiae* enolase 1 (strain ATCC 204508/S288c; PDB ID: 1EBG) [83], showing a sequence identity of 70.81%, was selected as a template model. (**a**) Protein surface view (blue) based on prediction (*p* = 5.1 × 10^−12^) using RaptorX [80]. Epitope 2 (Ep2) and epitope 7 (Ep7) are exclusive of *P. lutzii* and are indicated in the zoom area. A yellow dot indicates amino acid residues in the epitopes. (**b**) ConSurf analysis of the enolase protein structure of *P. lutzii* (accession number: XP_015703472) reveals that the 13 predicted B-cell epitopes (red asterisks) match exposed residues. Amino acid residues are color coded by their conservation grades using a nine-grade color-coding bar (bin 1 to 9), with turquoise-through-maroon indicating variable through conserved. The conservation calculation was performed on a sample of 150 sequences representing the list of homologs to the query XP_015703472. The figure also reveals the functionally and structurally important regions of the enolase.

Previous results by our group [22] showed that an immunodominant protein in PCM due to *P. lutzii* occurs in the range of 54 kDa, and our 2-D immunoblot revealed a large number of protein spots (Pl20, Pl21, Pl22, Pl23, Pl24, and Pl34) recognized by the *P. lutzii* antisera. The strongest reactors were observed in the 54 kDa range. The immunodominant protein in the *P. lutzii* mycosis was identified as enolase (XP_015703472), and characterization of its 3D molecular structure was used to highlight the putative epitopes to improve serodiagnosis of PCM due to *P. lutzii* (Figure 8a). The homology modeling found for *P. lutzii* enolase used the RaptorX software and revealed that enolase 1 from *Saccharomyces cerevisiae* (strain ATCC 204508/S288c; PDB ID: 1EBG) [83] was the best template model, showing a sequence identity of 70.81%. PROCHECK analyzed the quality of the model predicted by RaptorX for the evaluation of the Ramachandran plot quality. We found that 90.7% of the residues of this protein (351 amino acid residues) lay in the most favored region, while only three residues were in disallowed regions (0.8%), supporting the good quality of the predicted model (Supplementary Figure S2). A comparison between enolase homologs using BepiPred 2.0 revealed 13 epitopes in *P. lutzii* and 15 epitopes predicted in *P. brasiliensis*, with only four epitopes shared. We highlight epitope #2 (pos.40–60: STGQHEACELRDGDQSKWLGK) and epitope #7 (pos. 195–232: RLRSLSQTEGPRQEEVRQSAGNVGDEGGVAPDIQTPEE) in enolase, which are exclusive to *P. lutzii* and were shown by ConSurf analysis to be related to exposed residues of the globular protein according to the neural network algorithm (Figure 8b).

## 4. Discussion

The recent introduction of dissimilar species in *Paracoccidioides* has raised important questions about the immunodiagnosis of this disease. We highlight the paradoxical diagnosis in PCM, where the same patient may present positive mycological exams, with the observation of pathognomonic structures related to *Paracoccidioides* species, whereas serological tests, such as immunodiffusion, show no reactivity in the presence of the reference antigen AgPbB339 [15]. The phylogenetic recognition of *P. lutzii* brought the first clue to solving this problem [35,47], and currently regional antigenic preparations are recommended for use in areas of occurrence of this fungus, such as the Brazilian Midwest [12,13,22,85,86]. The second clue to solving this puzzle emerges from this study, where we performed immunoproteomic analysis of the *Paracoccidioides* yeast cells aiming to screen potential biomarkers for the refinement of the immunodiagnosis of PCM due to *P. lutzii*.

The 16 specific immunogenic proteins of *P. lutzii* can be divided into three main groups. Group 1 comprises seven proteins that have been widely reported in *Paracoccidioides* under experimental physiological conditions according to STRING analysis, namely, triosephosphate isomerase, glyceraldehyde-3-phosphate dehydrogenase, citrate synthase, isocitrate lyase, enolase, HSP60-like protein, and an uncharacterized protein (thioredoxin-like superfamily) [54,70,87,88,89,90,91,92,93]. Proteins included in group 2 are antigens that already have been described in human PCM, such as triosephosphate isomerase, glyceraldehyde-3-phosphate dehydrogenase, fructose-bisphosphate aldolase [70], or *Paracoccidioides* antigens that already have been related to eliciting a humoral immune response in BALB/c mice, such as transketolase, HSP60-like protein, and isocitrate lyase (all shared with PS3) [71]. Finally, proteins in the last group correspond to novel immunogens, described for the first time in *P. lutzii* mycosis, such as NAD(P)H: quinone oxidoreductase, type IV, phenylacetone monooxygenase, proteasome subunit alpha, mannitol-1-phosphate 5-dehydrogenase, HSP72-like protein, and two uncharacterized proteins (XP_015701261 and XP_002797075).

*Paracoccidioides brasiliensis s. str.* was used as a comparative group, and to our surprise only five proteins were found to be shared between *P. lutzii* and *P. brasiliensis* (triosephosphate isomerase, glyceraldehyde-3-phosphate dehydrogenase, fructose-bisphosphate aldolase 1, and HSP72-like protein, and HSP60, mitochondrial). The immunoproteome of human PCM due to *P. brasiliensis s. str.* shares only two proteins with those described as eliciting a humoral immune response in BALB/c mice, including HSP7-like protein (shared with PS2) and HSP 60-like protein (shared with *P. lutzii*, S1, PS2, and PS3) [71]. This suggests that more research on this neglected system will reveal a greater number of antigens shared between closely related cryptic species. The 25 specific immunogenic proteins of *P. brasiliensis* can be divided into three main groups. Group 1 comprises 13 proteins that have already been described in *Paracoccidioides* under experimental physiological condition, namely, triosephosphate isomerase, spermidine synthase, glyceraldehyde-3-phosphate dehydrogenase, 4-hydroxyphenylpyruvate dioxygenase, thioredoxin reductase, ATP synthase subunit beta, hexokinase, HSP75-like protein, HSP60-like protein, HSP7-like protein, aconitate hydratase, mitochondrial, 1-Cys peroxiredoxin, and an uncharacterized protein (XP_010758132) [54,70,87,88,89,90,91,92,93,94,95], all of them enriched in our STRING network. Proteins in group 2 correspond to antigens that have already been described in human PCM [70] or immunogens that have already been described to elicit a humoral immune response in BALB/c mice, such as HSP7-like protein and HSP 60-like protein [71]. Proteins in group 3 correspond to novel antigens, such as nucleoside diphosphate kinase, deoxyuridine 5′-triphosphate nucleotidohydrolase, proliferating cell nuclear antigen, 14-3-3 family protein epsilon, phosphomannomutase, HSP72-like protein, the acetyltransferase component of pyruvate dehydrogenase complex, disulfide-isomerase, and four uncharacterized proteins (XP_010759842, XP_010761900, XP_010761260, and XP_010758132) described for the first time in *P. brasiliensis* mycosis.

Among the proteins identified by us, most are involved in metabolic pathways (KEGG map01100), carbon metabolism (KEGG map01200), and biosynthesis of secondary metabolites (KEGG map01110) in both immunoproteomes (Supplementary Figure S3). Recently, Amaral et al. [54] reported that genes involved in gluconeogenesis are upregulated in highly virulent *Paracoccidioides* species, as the protein levels related to carbohydrate metabolism increase. Remarkable progress has been achieved to reveal the potential carbon sources during infection using *A. fumigatus, C. albicans*, and *C. neoformans* exposed to immune cells or in infection models [96,97,98,99]. Parallels between the response of these fungi to phagocytosis include enhancement of metabolic pathways required during carbon starvation, along with gluconeogenesis, fatty acid metabolism, and the glyoxylate shunt, many of which are targets of the carbon catabolite repression system in these fungi [100]. The increased expression levels of gluconeogenesis-related proteins during *Paracoccidioides* infection may expose these proteins to the host immune system. In this respect, proteins involved in the metabolic and carbon metabolism pathways were found to be recognized by circulating IgG antibodies in sera of patients that developed PCM due to *P. lutzii* and *P. brasiliensis s. str.*

Fungi evolved in a polyphyletic manner, developing different lifestyles, ranging from saprophytic to human pathogenic [101]. Consequently, their metabolic flexibility has been influenced by numerous evolutionary pressures [102,103]. Fungal metabolic pathways allow them to grow in diverse host niches and synthesize an array of potent small molecules [102,103]. Therefore, metabolism is essential to the *Paracoccidioides* pathogenicity, and there is significant importance of nitrogen [104], carbon [105], and micronutrient assimilation [91], allowing them to express key virulence factors [54].

The response of *Paracoccidioides* to macrophage infection shows that the fungus most likely faces a nutrient-limited environment in macrophage phagosomes, because a significantly higher expression of genes encoding isocitrate lyase was detected in earlier studies [92,105,106]. In *Candida albicans*, isocitrate lyase is a critical enzyme for virulence in the glyoxylate cycle, and its increased activity has been shown to be an important marker for gluconeogenic carbon source utilization and starvation [97,107]. Indeed, isocitrate lyase has been shown to elicit a humoral response in BALB/c mice challenged with exoantigens from members of the *P. brasiliensis* complex [71], and here, we found that isocitrate lyase is an antigen in PCM due to *P. lutzii*.

Interestingly, glyceraldehyde-3-phosphate dehydrogenase and fructose-bisphosphate aldolase are expressed in high levels among highly virulent *P. brasiliensis* complex isolates found to trigger disseminated disease in a murine model of PCM [54,106] and have been observed in *Paracoccidioides* extracellular vesicle preparations of the Pb18 isolate [90]. Therefore, the vesicular transport may deliver substances across the *Paracoccidioides* cell wall, possibly modulating the host’s immune response and supporting the high virulence of phenotypes [54,108]. Both proteins elicited humoral responses in human PCM due to *P. lutzii* and *P. brasiliensis s. str.*

**Figure 9:**
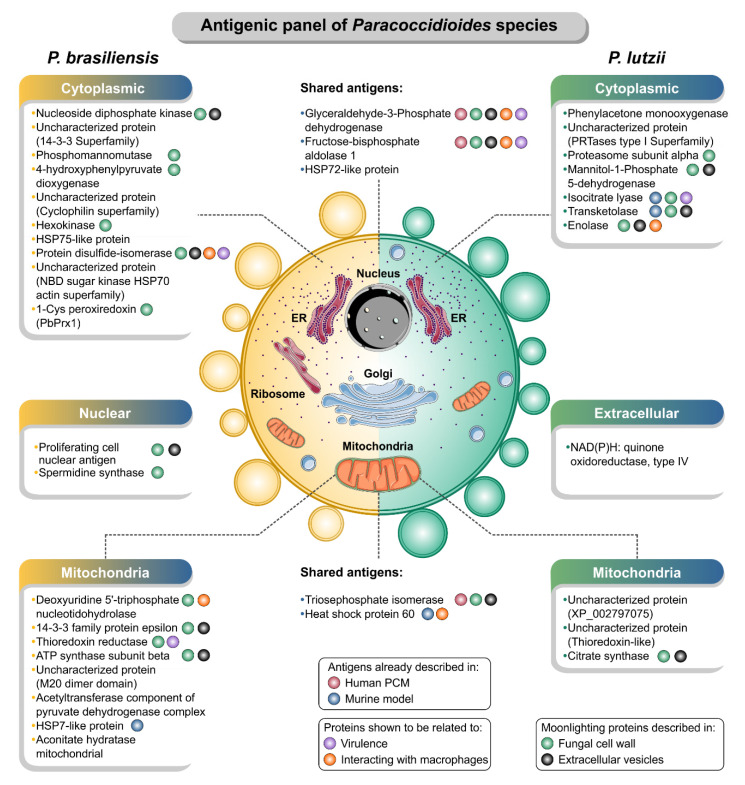
A panel of immunogenic proteins in paracoccidioidomycosis caused by *P. brasiliensis sensu stricto* (left) and *P. lutzii* (right). The immunogenic proteins were grouped according to the subcellular localization predicted by the WoLF PSORT [69]. Pieces of information about the report of these proteins as antigens in human [70] or murine PCM [71], the presence in the cell wall [95,116,126] and extracellular vesicles [90], during interaction with macrophages [122] or associated with virulence processes in *Paracoccidioides* [54,57,106] were noted. The illustration was partially based on Servier Medical Art elements and licensed under a Creative Commons Attribution 3.0 Unported License. ER: endoplasmic reticulum.

Proteins employed in carbon metabolism and metabolic or MAPK signaling pathways and that have been found in the *Paracoccidioides* cell walls are implicated in adhesion to matrix-associated components. We found that glyceraldehyde-3-phosphate dehydrogenase, enolase, triosephosphate isomerase, 14-3-3 protein, and fructose-1,6-bisphosphate aldolase [109,110,111,112,113,114,115,116] elicited IgG antibodies during PCM. *Paracoccidioides* moonlighting proteins have different functions inside and outside the cell. Many enzymes have multiple functions when secreted or when attached to the cell surface. Usually, the mechanisms behind the moonlighting proteins’ delivery and how some become attached to the cell surface are enigmatic [117]. Nevertheless, Vallejo et al. [90] proposed that extracellular vesicles act as an efficient and general mechanism of secretion of pathogenesis-related molecules in *Paracoccidioides*, delivering a concentrated payload of fungal products to host effector cells and tissues [118]. Here, we demonstrated that moonlighting proteins in *Paracoccidioides* elicited a humoral response in human PCM, so it is tempting to hypothesize that extracellular vesicles function as “virulence bags” [118], dispensing antigens during human PCM (Figure 9). The presence of these moonlighting proteins in vesicles produced by other fungi has already been demonstrated for *Histoplasma capsulatum* [119], *Cryptococcus neoformans* [118], and *Saccharomyces cerevisiae* [120]. These moonlighting proteins are usually present in increased levels in the fungus cell wall during interaction with host cells, suggesting they may be involved in host–parasite interactions and virulence [95,116,121,122,123]. Some of these proteins have already been demonstrated to be crucial vaccine candidates in pathogenic fungi (e.g., glyceraldehyde-3-phosphate dehydrogenase, enolase, 14-3-3 protein, and fructose-1,6-bisphosphate aldolase), since they are highly expressed and have low identity with homolog proteins in the human host [124,125].

Interestingly, the 43 kDa glycoprotein (gp43), a classic antigen of *P. brasiliensis* [127], also implicated in adhesion to matrix associated components [128], was not found in our immunoproteomes. However, a 1-D immunoblot using the purified gp43 protein [129] revealed that anti-gp43 antibodies were present in the serum pool of patients infected with *P. brasiliensis s. str.* (Supplementary Figure S4). Therefore, the lack of reaction in the 2-D immunoblot may be because the strain Pb18 (S1) used in our experiments is a weaker producer of gp43 compared to the isolate B-339 (PS3) [130,131], combined with technical limitations related to 2-D electrophoresis, where proteins can be lost during the purification and transfer process, making detection unfeasible [132]. Although common epitopes between *P. lutzii* and *P. brasiliensis* gp43 exist, anti-gp43 MAbs (MAb3e, Mab17c, Mab19g, MAb21f, or MAb32) do not recognize the recombining orthologous *P. lutzii* protein [133] nor did antibodies from patients infected with *P. lutzii* recognize the native B-339 [22], supporting the idea that B-339 gp43 cannot be used in the diagnosis of PCM caused by *P. lutzii* [133].

The serology of PCM due to *P. lutzii* draws attention to the intense reaction of a fraction oscillating in the range of 54 kDa in immunoblots [22,85], which has been proposed as the immunodominant antigen in *P. lutzii* mycosis [22]. However, the protein sequence of this immunogenic fraction had not been identified until the present study. In our experiments, we also observed an intense reaction in the range of 54 kDa, and interestingly, several spots were identified as enolase. The surface-associated enolase is upregulated in *Paracoccidioides* yeast cells derived from mouse-infected tissues [112], and in agreement with our results, it has been described as an isoform with increased abundance in the *P. lutzii* proteome [93]. This protein binds to plasminogen and mediates the interaction of yeast forms with host cells [134], suggesting that enolase may contribute to the pathogenesis of *Paracoccidioides* (Figure 9).

Moreover, it is well known that this adhesin can be inhibited by a specific antibody, influencing the fungus’ adhesion to pulmonary cells. Therefore, the presence of IgG antibodies against *P. lutzii* enolase in the serum of patients supports the importance of these molecules during the host–pathogen interplay [111,135], and it is tempting to hypothesize that post-translational modification of enolase is responsible for regulation of the immune response during *P. lutzii* infection. A monoclonal antibody raised against the enolase cell-surface protein of *Aspergillus fumigatus* exhibited diagnostic and therapeutic potential, inhibiting spore germination and presenting a fungicidal activity against a broad range of *Aspergillus* infections [136]. Future studies with the recombinant enolase protein or enolase B-cell epitopes may elucidate the specificity and sensitivity of antienolase antibodies’ detection and refine the immunodiagnosis of *P. lutzii* mycosis.

Energy production pathways in yeast cells of *P. lutzii* are driven by glycolysis and fermentation, whereas *P. brasiliensis* yeast cells preferentially use aerobic betaoxidation and the citrate cycle (TCA cycle) for ATP production [91]. Proteins related to the oxidative phosphorylation and TCA cycle include citrate synthase, aconitate hydratase, ATP synthase subunit beta, and acetyltransferase component of pyruvate dehydrogenase complex, which were found to be immunogenic during PCM. Citrate synthase catalyzes the entry of carbon into the citric acid cycle, and in *Paracoccidioides*, methylcitrate synthase transcripts and proteins and aconitate hydratase are upregulated throughout the adaptation to environmental conditions during the increase in the temperature [91]. Experimental data shows that *P. lutzii* presents an elevated methylcitrate synthase activity even when glucose is the sole carbon source, and two-dimensional Western blot data revealed a different pattern in isoform distribution with low mass variation, suggesting the presence of varying phosphorylation patterns [137]. Here, a single spot citrate synthase was recognized by antibodies circulating in sera of patients with PCM due to *P. lutzii*. ATP synthase subunit beta is one of the *P. lutzii*-secreted proteins that interacts with macrophages [122], and here, it was recognized by IgG in the sera of *P. brasiliensis* mycosis patients. F-ATP synthases are molecular rotary motors that catalyze ATP synthesis from ADP and inorganic phosphate using the proton-motive force generated by substrate-driven electron transfer chains [138]. Acetyltransferase component of pyruvate dehydrogenase complex, a gene involved in the TCA cycle, was found to be IgG-reactive in PCM due to *P. brasiliensis* although experimental data shows that it is downregulated upon 6 h postinfection of mouse lung compared with the in vitro control [106] (Figure 9).

Proteins related to DNA replication and mismatch repairs such as the proliferating cell nuclear antigen (PCNA) and deoxyuridine 5′-triphosphate nucleotidohydrolase (dUTPase) were found to be immunogenic in the system *P. brasiliensis*-PCM. dUTPase (EC 3.6.1.23) catalyzes the conversion of dUTP to dUMP and pyrophosphate (PPi), providing the substrate for methylation of uracil by thymidylate synthase and preventing accidental incorporation of uracil into DNA by DNA-polymerase [139,140]. Interestingly, dUTPase has been described in the secretome of *P. lutzii,* and experimental data show that this protein interacts with macrophage proteins [122]. Notwithstanding, PCNA is described for the first time as an antigen in PCM. PCNA synthesis correlates with the proliferative state of the cell that has been found in the nuclei of yeasts, plants, and animal cells that undergo cell division, suggesting a function in cell cycle regulation and DNA replication [141,142]. Afterward, PCNA was shown to also play an essential role in other processes involving the cell genome [141,142], and in *Paracoccidioides,* it has been observed in the cell wall [116].

A total of seven antigenic proteins detected in our analysis matched uncharacterized protein. This finding was not surprising as Desjardins et al. [143] reported that approximately 60% of *Paracoccidioides* genes were encoding hypothetical proteins with undetermined cellular functions with no evidence of in vivo expression [144]. Such finding is common in *Paracoccidioides* species [93] as it has poor databases and a lack of proteomic studies compared to model organisms. To the extent of our knowledge, this is the first report describing the hypothetical proteins XP_015701261 (PRTases type I superfamily) and XP_002797075 as antigens in *P. lutzii* immunoproteome. Both proteins were predicted to be antigens by AntigenPRO with high scores. Interestingly, the uncharacterized protein XP_002797075 was predicted to be in the mitochondria of *Paracoccidioides*. As antibodies were from infected patients, this strongly suggests that these proteins are expressed in vivo during PCM.

A hypothetical protein (XP_015700719) containing domains with a thioredoxin-like fold was immunogenic in *P. lutzii* proteome. Recent studies investigating the humoral response in BALB/c mice revealed antibodies targeting an uncharacterized protein with a thioredoxin-like superfamily domain in PS3 secretome [71], similar to the response found in our study in human PCM. Likewise, other four uncharacterized protein was found to be immunogenic exclusively in the *P. brasiliensis* immunoproteome, including a hypothetical protein containing a 14-3-3 superfamily domain (XP_010759842), a cyclophilin superfamily domain (XP_010761900), an M20_dimer domain-containing protein (XP_010761260), and an NBD sugar kinase superfamily (XP_010758132). Indeed, in silico characterization of *P. brasiliensis* hypothetical proteins sequences using AntigenPRO and VaxiJen predicted them to be antigens with scores above 0.5. Further studies will be needed to characterize all these hypothetical proteins and understand its role in PCM infection.

During infection, nutrient starvation results in common stress for fungal pathogens, leading to primary and secondary metabolism changes. Secondary metabolites are crucial players in fungal development and actively shape interactions with other organisms [145]. A cluster of proteins related to the biosynthesis of amino acids and purine metabolism was immunogenic in PCM, including nucleoside diphosphate kinase, spermidine synthase, 4-hydroxyphenylpyruvate dioxygenase, which are found in the *Paracoccidioides* species’ cell wall [116] (Figure 9). Spermidine synthase and 4-hydroxyphenylpyruvate dioxygenase are among the most abundant upregulated proteins of *P. lutzii* yeast cells under carbon starvation [105]. In contrast, nucleoside diphosphate kinase was described as an adhesin-like molecule, identified during copper-deprivation conditions in *P. lutzii* in the presence of extracellular matrix components [146].

Four molecular chaperones named “HSP60, HSP72, HSP75, and HSP7” were identified as antigens in *P. lutzii* or *P. brasiliensis* proteomes. Molecular chaperones, usually known as heat shock proteins, are a diverse family of proteins that are upregulated under conditions of stress and operate to protect proteins from irreversible aggregation during synthesis and in times of cellular stress [147,148]. These molecular chaperones have been demonstrated to be modulated during the nitrosative stress in *P. brasiliensis*, suggesting that they play essential roles in fungal virulence [149]. Remarkably, the HSP60 protein, have been primarily explored in fungal cells and recognized to be crucial for cell growth [150], survival in the host, morphogenesis [151,152], germination, and conidiation [153]. Immune responses to the three molecular chaperones (HSP60, HSP70, and HSP7) elicit the production of both specific antibodies in BALB/c mice challenged with proteins secreted by *Paracoccidioides* species [71].

The thioredoxin system, a central antioxidant system in nearly all living cells, comprises thioredoxin, thioredoxin reductase, and NADPH [154]. A cluster of proteins involved in the pentose phosphate pathway, including thioredoxin reductase, hexokinase, mannitol-1-phosphate 5-dehydrogenase, NAD(P)H: quinone oxidoreductase, type IV, and phenylacetone monooxygenase, were recognized by antibodies circulating in the sera of patients with PCM. Proteomic analyses demonstrated that thioredoxin reductase was induced in *Paracoccidioides* during murine infection [106], and along with hexokinase, they were detected among the group of upregulated proteins upon carbon starvation in *Paracoccidioides* [105]. Hexokinase is present in the secretome and in the cell wall of *Paracoccidioides* species [116,155]. Mannitol-1-phosphate 5-dehydrogenase was described as a virulence factor in *Paracoccidioides*, and we found that this protein elicits IgG antibodies during *P. lutzii* infection. In pathogenic fungi, the improved stress resistance resulting from accumulated six-carbon polyol D-mannitol enhances the ability to deal with defense strategies of the infected host [156]. Mannitol is known to quench reactive oxygen species (ROS) [157], and there is some evidence that fungi use mannitol to suppress ROS-mediated host defenses [156,158]. NAD(P)H: quinone oxidoreductase, type IV and phenylacetone monooxygenase, two enzymes that belong to the family of oxidoreductases and catalyze the transfer of electrons from oxidant to reductant [159,160,161], were identified as antigens in the proteome of *P. lutzii*. Indeed, cytosolic quinone reductase is a critical component of pathogenicity during the host–fungus interaction [162,163].

A unique *P. brasiliensis* 1-Cys peroxiredoxin (PbPrx1; XP_010758730) was only recently reported to localize in the cytoplasm and at the cell wall of the yeast and mycelial forms of *P. brasiliensis*, as well as in the yeast mitochondria [95]. Longo et al. suggest a possible role of PbPrx1 in the fungal antioxidant defense mechanisms [95], and here, we report PbPrx1 as an antigen in human PCM due to *P. brasiliensis s. str*. Interestingly, in plants, 1-Cys peroxiredoxin has been shown to not only relieve oxidative stresses but also play a central role as molecular chaperones under severe conditions during seed germination, and that overoxidation controls the switch in function of 1-Cys-peroxiredoxins from peroxidases to molecular chaperones [164]. In fungal pathogens, analyses of the three 1-Cys peroxiredoxins from *A. fumigatus* reveal that they act together to maintain the redox balance, playing a significant role in pathogenicity [165], two aspects that could be regulated by antibodies during *Paracoccidioides* infection.

Phosphomannomutase was found to be immunogenic in the system *P. brasiliensis*-PCM. This enzyme catalyzes the interconversion of mannose-6-phosphate and mannose-1-phosphate, and in *Saccharomyces cerevisiae*, phosphomannomutase (Sec53p) is essential for viability [155]. However, a proteomic study found that phosphomannomutase is preferentially secreted in *P. lutzii* compared to *P. restrepiensis* [155]. A sequence related to protein degradation, such as the proteasome subunit alpha, was identified as antigenic in *P. lutzii* mycosis. Proteasome is a highly sophisticated protease complex that promotes selective and efficient processive hydrolysis of intracellular client proteins in eukaryotic cells, a process that requires metabolic energy [166].

In the second part of our study, we analyzed the prediction of linear epitopes for B-cells. It is important to mention that epitope-based antibodies are currently the most promising classes of biopharmaceutical protein products [167]. Significant advances in immunoinformatics tools have revolutionized the development of diagnostic assays and vaccine design [168]. The latest tools available for analyzing antigenic properties and epitopes associated with the pathogen include AntigenPRO, BCPred, SVMTriP, COBEpro, and IEDB tools like BepiPred, Ellipro, etc. [169,170,171]. The identification of *Paracoccidioides* antigens was followed by in silico analysis to predict B-cell epitope, producing a library of 219 and 379 epitopes predicted for *P. lutzii* and *P. brasiliensis*, respectively. These are all good candidates for serological diagnosis of PCM. Interestingly, two synthetic peptides, named “P2 and L15,” have been proposed for the serodiagnosis of PCM [172,173], and our panel of epitopes can significantly increase in the number of peptides used in immunodiagnosis of PCM in the coming years.

The lack of good-quality diagnostic tests for *Paracoccidioides* infection causes slow diagnosis, and consequently poor prognosis of this neglected mycosis in the Americas. Since PCM’s clinical manifestations lack specificity, confirmatory tests are required to identify humans infected with *Paracoccidioides* spp. Further studies are under way to map these epitopes in vitro. Several antibody-detection assays employing crude preparations or recombinant proteins have been developed for laboratory diagnosis of PCM [17,18,19,21,24,25,26,48,174,175,176,177,178,179]. However, a diagnostic method with high specificity and sensitivity to guide the management and control remains to be developed for *P. lutzii*. We believe that a new generation of diagnostic tests with the expected high sensitivity and specificity should be composed of various linear B-cell epitopes, mapped in silico from the *Paracoccidioides* antigens identified in this study. The current clinical data on PCM have shown great overlap of clinical signs due to distinct *Paracoccidioides* species [12,14,180]. Therefore, the best strategy for the immunodiagnosis still involves differentiation between *P. lutzii* and members of the *P. brasiliensis* complex instead of stressing a multimarker diagnostic assay, which will likely pose a major challenge regarding specificity and sensitivity for successful implementation. A sensitive and affordable point-of-care assay is mandatory for an important neglected mycosis such as PCM, which is usually associated with poverty.

## 5. Conclusions

Our study was carried out using proteomic and in silico analysis, and we were able to identify novel antigens that represent a panel of key targets for humoral response against *P. lutzii* and *P. brasiliensis s. str.*, the most common agents of PCM in a vast area of the Americas. This is the first study to report specific antigens in the yeast phase of cryptic *Paracoccidioides* species using a gold-standard human serum. We identified the major antigen of PCM due to *P. lutzii* as enolase, and several B-cell epitopes were predicted in the *Paracoccidioides*-PCM system. Hypothetical proteins were found to elicit an antibody response in human PCM, so were identified as antigens. Further studies employing some of these native and recombinant proteins will be conducted to develop an accurate diagnostic test and an effective vaccine, identify infected hosts, and prevent infection and development of human PCM. The findings reported here provide a unique opportunity for the refinement of diagnostic tools of this important neglected systemic mycosis.

## Figures and Tables

**Table 1:** Identification of IgG-reactive proteins recognized by sera of patients with paracoccidioidomycosis due to *Paracoccidioides lutzii*.

Spot Number	Accession Number	Gene Name	Protein Name	Mass (Mr)	pI	Cov. (%)	Score
Pl01, Pl03, Pl04	XP_015702763	PADG_07749	NAD(P)H: quinone oxidoreductase, type IV	23,585	6.21	16	228
Pl02	XP_015700719	PADG_02764	Uncharacterized protein (Thioredoxin-like Superfamily)	30,945	8.45	14	124
Pl05	XP_002795879	PADG_06906	Triosephosphate isomerase	27,159	5.39	24	408
Pl06	XP_002797384	PADG_03581	Phenylacetone monooxygenase	68,182	5.95	1	33
Pl07	XP_015701261	PADG_00322	Uncharacterized protein (PRTases type I Superfamily)	23,117	5.28	22	186
Pl08	XP_002797532	PADG_07190	Proteasome subunit alpha	29,747	6.36	3	39
Pl09, Pl10	Q8X1X3	PADG_02411	Glyceraldehyde-3-Phosphate dehydrogenase	36,619	8.26	10	185
Pl11	XP_002796107	PADG_00668	Fructose-bisphosphate aldolase 1	39,721	6.09	18	429
Pl12	XP_002791734	PADG_01372	Mannitol-1-Phosphate 5-dehydrogenase	43,119	5.66	3	78
Pl13, Pl30, Pl31, Pl32	XP_002790117	PADG_08118	HSP72-like protein	70,919	5.08	9	390
Pl14	XP_002793640	PADG_04710	Citrate synthase	51,516	9.02	16	307
Pl16	XP_002791040	PADG_01483	Isocitrate lyase	60,173	6.79	2	26
Pl17, Pl19	XP_002793534	PADG_04604	Transketolase	74,940	5.97	2	28
Pl20, Pl21, Pl22, Pl23, Pl24, Pl34	XP_015703472	PADG_04059	Enolase	48,515	5.49	19	389
Pl27, Pl28, Pl29	XP_002789992	PADG_08369	HSP60, mitochondrial	62,266	5.51	13	510
Pl36	XP_002797075	PADG_03893	Uncharacterized protein	38,092	8.46	3	26

**Table 2:** Identification of IgG-reactive proteins recognized by sera of patients with paracoccidioidomycosis due to *Paracoccidioides brasiliensis sensu stricto*.

Spot Number	Accession Number	Gene Name	Protein	Mass (Mr)	pI	Cov. (%)	Score
Pb37	XP_010762888	PADG_07524	Nucleoside diphosphate kinase	16,908	6.84	23	226
Pb38, Pb40, Pb41, Pb44	XP_010758730	PADG_03095	1-Cys peroxiredoxin (PbPrx1)	24,931	5.28	17	175
Pb45	XP_010763035	PADG_07782	Deoxyuridine 5′-triphosphate nucleotidohydrolase	22,190	5.27	12	75
Pb46	XP_010762134	PADG_06906	Triosephosphate isomerase	27,176	5.39	10	66
Pb48	XP_010759909	PADG_11679	Proliferating cell nuclear antigen	36,276	4.87	11	205
Pb49	XP_010759842	PADG_04440	Uncharacterized protein (14-3-3 Superfamily)	29,738	4.68	24	322
Pb50	XP_010759301	PADG_04056	14-3-3 family protein epsilon	32,531	4.74	19	240
Pb52	XP_010760066	PADG_04603	Spermidine synthase	33,714	5.33	8	220
Pb53	XP_010759656	PADG_03943	Phosphomannomutase	30,560	5.6	5	33
Pb54, Pb55, Pb56	XP_010758444	PADG_02411	Glyceraldehyde-3-phosphate dehydrogenase	37,397	7.12	12	232
Pb57, Pb58, Pb59, Pb60	XP_010756450	PADG_00668	Fructose-bisphosphate aldolase 1	39,764	6.28	14	391
Pb62, Pb70	XP_010763814	PADG_08468	4-hydroxyphenylpyruvate dioxygenase	45,844	5.64	3	37
Pb65	XP_010761900	PADG_06488	Uncharacterized protein (cyclophilin superfamily)	41,276	5.42	15	352
Pb66, Pb68, Pb69, Pb83, Pb84, Pb85	XP_010763478	PADG_08118	HSP72-like protein	70,949	5.08	14	513
Pb67	XP_010756960	PADG_01551	Thioredoxin reductase	38,134	5.42	7	83
Pb71, Pb72, Pb73	XP_010763627	PADG_08349	ATP synthase subunit beta	66,837	6.01	12	423
Pb74	XP_010763342	PADG_07950	Hexokinase	55,065	5.19	13	224
Pb75	XP_010758595	PADG_02761	HSP75-like protein	67,330	5.27	9	446
Pb76	XP_010761260	PADG_05922	Uncharacterized protein (M20_dimer domain-containing protein)	63,634	6.36	6	145
Pb78	XP_010762465	PADG_07213	Acetyltransferase component of pyruvate dehydrogenase complex	55,658	7.67	3	93
Pb79, Pb80, Pb81	XP_010763632	PADG_08369	HSP60, mitochondrial	62,522	5.51	16	797
Pb82, Pb87	XP_010759621	PADG_03841	Protein disulfide isomerase	58,878	4.88	15	498
Pb86, Pb88	XP_010756347	PADG_00430	HSP7-like protein	94,608	6.4	8	393
Pb89, Pb90	XP_010758132	PADG_02785	Uncharacterized protein (NBD sugar kinase HSP70 actin superfamily)	80,759	4.91	9	205
Pb92, Pb93	XP_010760563	PADG_11845	Aconitate hydratase, mitochondrial	80,073	6.46	8	136

**Table 3:** Characterization of full-length immunoreactive proteins of *Paracoccidioides* spp., for predicted antigenicity using AntigenPRO and VaxiJen.

*P. lutzii* Accession	*P. brasiliensis* Accession	Protein Name	Localization WoLF PSORT	Pb01 AntigenPRO	Pb01 VaxiJen	Pb01 No. of Epitopes	Pb18 AntigenPRO	Pb18 VaxiJen	Pb18 No. of Epitopes	Shared Epitopes
XP_015702763	XP_010763025	NAD(P)H: quinone oxidoreductase, type IV	Extracellular	0.6407	0.4315	7	0.7233	0.5125	6	0
XP_015700719	XP_010758121	Uncharacterized protein (thioredoxin-like superfamily)	Mitochondrial	0.9448	0.6254	12	0.9222	0.6070	10	3
XP_002795879	XP_010762134	Triosephosphate isomerase	Mitochondrial	0.6495	0.7753	11	0.7712	0.7536	13	7
XP_002797384	XP_010759514	Phenylacetone monooxygenase	Cytoplasmic	0.8696	0.5354	17	0.8388	0.4979	16	0
XP_015701261	XP_010756299	Uncharacterized protein (PRTases type I Superfamily)	Cytoplasmic	0.8444	0.7386	5	0.7880	0.7390	5	1
XP_002797532	XP_010762451	Proteasome subunit alpha	Cytoplasmic	0.8091	0.6841	9	0.6377	0.6426	9	2
Q8X1X3	XP_010758444	Glyceraldehyde-3-Phosphate dehydrogenase	Cytoplasmic	0.8114	0.7938	11	0.7921	0.8081	12	5
XP_002796107	XP_010756450	Fructose-bisphosphate aldolase 1	Cytoplasmic	0.7233	0.4472	11	0.7153	0.4869	13	6
XP_002791734	XP_010757428	Mannitol-1-Phosphate 5-dehydrogenase	Cytoplasmic	0.6436	0.5805	14	0.7117	0.5592	16	2
XP_002790117	XP_010763478	HSP72-like protein	Cytoplasmic	0.8263	0.6064	20	0.8291	0.6046	23	5
XP_002793640	XP_010760342	Citrate synthase	Mitochondrial	0.3002	0.4058	20	0.3103	0.4266	16	7
XP_002791040	XP_010757468	Isocitrate lyase	Cytoplasmic	0.8288	0.4291	19	0.8450	0.3904	19	4
XP_002793534	XP_010760067	Transketolase	Cytoplasmic	0.6889	0.7441	15	0.8113	0.6775	18	6
XP_015703472	XP_010759701	Enolase	Cytoplasmic	0.6237	0.8143	13	0.6419	0.7853	15	4
XP_002789992	XP_010763632	HSP60, mitochondrial	Mitochondrial	0.5421	0.9471	25	0.5124	0.9500	22	6
XP_002797075	XP_010759641	Uncharacterized protein	Mitochondrial	0.8841	0.4142	10	0.8729	0.5637	8	1
XP_002794019	XP_010762888	Nucleoside diphosphate kinase	Cytoplasmic	0.5476	0.5195	6	0.6612	0.5236	6	5
XP_002794671	XP_010758730	1-Cys peroxiredoxin (PbPrx1)	Cytoplasmic	0.8698	0.8177	9	0.8434	0.8314	10	3
XP_002792326	XP_010763035	Deoxyuridine 5’-triphosphate nucleotidohydrolase	Mitochondrial	0.9407	0.3919	3	0.9382	0.3264	3	1
XP_015700963	XP_010759909	Proliferating cell nuclear antigen	Nuclear	0.7182	0.6226	7	0.7234	0.7115	21	1
XP_002791205	XP_010759842	Uncharacterized protein (14-3-3 Superfamily)	Cytoplasmic	0.8629	0.5059	14	0.8728	0.5530	12	6
XP_002796914	XP_010759301	14-3-3 family protein epsilon	Mitochondrial	0.8586	0.5232	14	0.8844	0.5074	15	8
XP_002793533	XP_010760066	Spermidine synthase	Nuclear	0.3752	0.4093	11	0.4290	0.4429	8	3
XP_002797030	XP_010759656	Phosphomannomutase	Cytoplasmic	0.8278	0.6743	12	0.8003	0.6633	11	0
XP_002789912	XP_010763814	4-hydroxyphenylpyruvate dioxygenase	Cytoplasmic	0.7016	0.5006	11	0.7664	0.4948	14	4
XP_015701903	XP_010761900	Uncharacterized protein (cyclophilin superfamily)	Cytoplasmic	0.9025	0.5236	13	0.9179	0.5328	11	4
XP_002791109	XP_010756960	Thioredoxin reductase	Mitochondrial	0.8249	0.5353	12	0.8541	0.5592	12	3
XP_002789970	XP_010763627	ATP synthase subunit beta	Mitochondrial	0.4385	0.6881	25	0.2767	0.7620	21	8
XP_002791907	XP_010763342	Hexokinase	Cytoplasmic	0.3520	0.8065	20	0.3815	0.8873	21	6
XP_015700721	XP_010758595	HSP75-like protein	Cytoplasmic	0.8588	0.7374	21	0.8632	0.7372	22	11
XP_002789172	XP_010761260	Uncharacterized protein (M20_dimer domain)	Mitochondrial	0.6981	0.7508	22	0.5960	0.6827	23	11
XP_002797511	XP_010762465	Acetyltransferase component of pyruvate dehydrogenase complex	Mitochondrial	0.6820	0.8293	13	0.6725	0.6868	14	4
XP_002797127	XP_010759621	Protein disulfide-isomerase	Cytoplasmic	0.8475	0.5827	19	0.8856	0.7384	19	4
XP_015703361	XP_010756347	HSP7-like protein	Mitochondrial	0.7989	0.7184	27	0.9019	0.7541	27	4
XP_002790451	XP_010758132	Uncharacterized protein (NBD sugar kinase superfamily)	Cytoplasmic	0.8700	0.7442	26	0.82411	0.7502	25	5
XP_015699682	XP_010760563	Aconitate hydratase, mitochondrial	Mitochondrial	0.7657	0.6783	27	0.766503	0.6689	23	10

## Supplementary Materials

The following are available online at https://www.mdpi.com/2309-608X/6/4/357/s1, Figure S1: Anti-*Paracoccidioides* antibody titers in reactive sera of patients with paracoccidioidomycosis due to *P. brasiliensis sensu stricto* (S1; *n* = 10) or *P. lutzii* (*n* = 10). NHS: normal human serum (sera from the healthy normal donors; *n* = 10). A quantitative double immunodiffusion assay (DID) was used to establish the titration of each patient’s serum using (a) the exoantigens from the reference B-339 strain (AgPbB339; PS3) or (b) a *P. lutzii* CFA preparation derived from the reference isolate EPM208 as described earlier [21,22]. Each dot represents the result of a single patient. Bars represent the lowest (Min) and the highest (Max) value in each column. Figure S2: Ramachandran plot generated by PROCHECK validation server [81] showing the stereochemical quality of the *Paracoccidioides lutzii* enolase structure (accession number: XP_015703472). Figure S3: Overview of the metabolic pathways in (a) *P. lutzii* and (b) *P. brasiliensis* proteomes. The red lines represent proteins (IgG immunoreactive) that were highly enriched, and the boxes represent metabolic pathways. These metabolic maps were visualized and analyzed by the web application iPath3.0 [68]. Figure S4: Representative immunoblot of purified gp43 antigen (B-339 strain) using sera from patients with confirmed paracoccidioidomycosis due to *P. brasiliensis s. str.* (Pb, yellow lane) or *P. lutzii* (Pl, green lane). Table S1: BepiPred Linear Epitope Prediction 2.0 results.

## References

[1] Lutz A. (1908). Uma micose pseudococídica localizada na boca e observada no Brasil: Contribuição ao conhecimento das hifoblastomicoses americanas. Bras. Med..

[2] De Almeida F. (1930). Estudos comparativos do granuloma coccidióidico nos Estados Unidos e no Brasil. Novo gênero para o parasito brasileiro. An. Fac. Med. Sao Paulo.

[3] Brummer E., Castaneda E., Restrepo A. (1993). Paracoccidioidomycosis: An update. Clin. Microbiol. Rev..

[4] Restrepo A., McEwen J.G., Castaneda E. (2001). The habitat of *Paracoccidioides brasiliensis*: How far from solving the riddle?. Med. Mycol..

[5] Bocca A.L., Amaral A.C., Teixeira M.M., Sato P.K., Shikanai-Yasuda M.A., Soares Felipe M.S. (2013). Paracoccidioidomycosis: Eco-epidemiology, taxonomy and clinical and therapeutic issues. Future Microbiol..

[6] Queiroz-Telles F., Fahal A.H., Falci D.R., Caceres D.H., Chiller T., Pasqualotto A.C. (2017). Neglected endemic mycoses. Lancet Infect. Dis..

[7] Colombo A.L., Tobon A., Restrepo A., Queiroz-Telles F., Nucci M. (2011). Epidemiology of endemic systemic fungal infections in Latin America. Med. Mycol..

[8] De Deus Vieira G., da Cunha Alves T., de Lima S.M.D., Camargo L.M.A., de Sousa C.M. (2014). Paracoccidioidomycosis in a western Brazilian Amazon State: Clinical-epidemiologic profile and spatial distribution of the disease. Rev. Soc. Bras. Med. Trop..

[9] Paniago A.M.M., Aguiar J.I.A., Aguiar E.S., da Cunha R.V., de Pereira G.R.O.L., Londero A.T., Wanke B. (2003). Paracoccidioidomycosis: A clinical and epidemiological study of 422 cases observed in Mato Grosso do Sul. Rev. Soc. Bras. Med. Trop..

[10] Coutinho Z.F., Silva D., Lazera M., Petri V., Oliveira R.M., Sabroza P.C., Wanke B. (2002). Paracoccidioidomycosis mortality in Brazil (1980–1995). Cad. Saude Publica.

[11] San-Blas F., Cova L.J. (1975). Growth curves of the yeast-like form of *Paracoccidioides brasiliensis*. Sabouraudia.

[12] Hahn R.C., Rodrigues A.M., Della Terra P.P., Nery A.F., Hoffmann-Santos H.D., Góis H.M., Fontes C.J., de Camargo Z.P. (2019). Clinical and epidemiological features of paracoccidioidomycosis due to *Paracoccidioides lutzii*. PLoS Negl. Trop. Dis..

[13] Hahn R.C., Rodrigues A.M., Fontes C.J., Nery A.F., Tadano T., de Padua Queiroz Junior L., de Camargo Z.P. (2014). Fatal Fungemia due to *Paracoccidioides lutzii*. Am. J. Trop. Med. Hyg..

[14] Pereira E.F., Gegembauer G., Chang M.R., Camargo Z.P.D., Nunes T.F., Ribeiro S.M., Carvalho L.R.D., Maldonado B.M., Mendes R.P., Paniago A.M.M. (2020). Comparison of clinico-epidemiological and radiological features in paracoccidioidomycosis patients regarding serological classification using antigens from *Paracoccidioides brasiliensis* complex and *Paracoccidioides lutzii*. PLoS Negl. Trop. Dis..

[15] Camargo Z.P., Rodrigues A.M., Cordeiro R.A. (2019). *Paracoccidioides* Complex. Pocket Guide to Mycological Diagnosis.

[16] Pinheiro B.G., Hahn R.C., Camargo Z.P., Rodrigues A.M. (2020). Molecular tools for detection and identification of *Paracoccidioides* species: Current status and future perspectives. J. Fungi.

[17] Marques da Silva S.H., Queiroz-Telles F., Colombo A.L., Blotta M.H.S.L., Lopes J.D., de Camargo Z.P. (2004). Monitoring gp43 antigenemia in paracoccidioidomycosis patients during therapy. J. Clin. Microbiol..

[18] Marques da Silva S.H., de Mattos Grosso D., Lopes J.D., Colombo A.L., Blotta M.H.S.L., Queiroz-Telles F., de Camargo Z.P. (2004). Detection of *Paracoccidioides brasiliensis* gp70 circulating antigen and follow-up of patients undergoing antimycotic therapy. J. Clin. Microbiol..

[19] Marques-da-Silva S.H., Colombo A.L., Blotta M.H.S.L., Lopes J.D., Queiroz-Telles F., de Camargo Z.P. (2003). Detection of circulating gp43 antigen in serum, cerebrospinal fluid, and bronchoalveolar lavage fluid of patients with paracoccidioidomycosis. J. Clin. Microbiol..

[20] Martins R., Marques S., Alves M., Fecchio D., de Franco M.F. (1997). Serological follow-up of patients with paracoccidioidomycosis treated with itraconazole using Dot-blot, ELISA and western-blot. Rev. Inst. Med. Trop. Sao Paulo.

[21] de Camargo Z.P., Unterkircher C., Campoy S.P., Travassos L.R. (1988). Production of *Paracoccidioides brasiliensis* exoantigens for immunodiffusion tests. J. Clin. Microbiol..

[22] Gegembauer G., Araujo L.M., Pereira E.F., Rodrigues A.M., Paniago A.M., Hahn R.C., de Camargo Z.P. (2014). Serology of paracoccidioidomycosis due to *Paracoccidioides lutzii*. PLoS Negl. Trop. Dis..

[23] Bellissimo-Rodrigues F., Vitali L.H., Martinez R. (2010). Serological diagnosis of paracoccidioidomycosis in HIV-coinfected patients. Mem. Inst. Oswaldo Cruz.

[24] De Camargo Z.P., Guesdon J.L., Drouhet E., Improvisi L. (1984). Enzyme-linked immunosorbent assay (ELISA) in the paracoccidioidomycosis. Comparison with counterimmunoelectrophoresis and erythro-immunoassay. Mycopathologia.

[25] Dos Santos P.O., Rodrigues A.M., Fernandes G.F., da Silva S.H., Burger E., de Camargo Z.P. (2015). Immunodiagnosis of paracoccidioidomycosis due to *Paracoccidioides brasiliensis* using a latex test: Detection of specific antibody anti-gp43 and specific antigen gp43. PLoS Negl. Trop. Dis..

[26] Camargo Z.P., Unterkircher C., Travassos L.R. (1989). Identification of antigenic polypeptides of *Paracoccidioides* brasiliensis by immunoblotting. Med. Mycol..

[27] Camargo Z.P. (2008). Serology of paracoccidioidomycosis. Mycopathologia.

[28] Matute D.R., McEwen J.G., Puccia R., Montes B.A., San-Blas G., Bagagli E., Rauscher J.T., Restrepo A., Morais F., Niño-Vega G. (2006). Cryptic speciation and recombination in the fungus *Paracoccidioides brasiliensis* as revealed by gene genealogies. Mol. Biol. Evol..

[29] Matute D.R., Sepulveda V.E., Quesada L.M., Goldman G.H., Taylor J.W., Restrepo A., McEwen J.G. (2006). Microsatellite analysis of three phylogenetic species of *Paracoccidioides brasiliensis*. J. Clin. Microbiol..

[30] Morais F.V., Barros T.F., Fukada M.K., Cisalpino P.S., Puccia R. (2000). Polymorphism in the gene coding for the immunodominant antigen gp43 from the pathogenic fungus *Paracoccidioides brasiliensis*. J. Clin. Microbiol..

[31] Hebeler-Barbosa F., Morais F.V., Montenegro M.R., Kuramae E.E., Montes B., McEwen J.G., Bagagli E., Puccia R. (2003). Comparison of the sequences of the internal transcribed spacer regions and PbGP43 genes of *Paracoccidioides brasiliensis* from patients and armadillos (*Dasypus novemcinctus*). J. Clin. Microbiol..

[32] Del Negro G., Garcia N., Rodrigues E., Assis C., Melo N., Lacaz C.D.S. (1993). Note on *Paracoccidioides tenuis* Moore 1938 a possible synonym for *Paracoccidioides brasiliensis*. Rev. Iberoam. Micol..

[33] Moore M. (1935). A new species of the *Paracoccidioides* Almeida (1930): *P. cerebriformis* Moore, (1935). Rev. Biol. Hig..

[34] Gezuele E. Aislamiento de *Paracoccidioides* sp. de heces de pinguino de la Antartida. Proceedings of the IV International Symposium on Paracoccidioidomycosis.

[35] Teixeira M.M., Theodoro R.C., de Carvalho M.J.A., Fernandes L., Paes H.C., Hahn R.C., Mendoza L., Bagagli E., San-Blas G., Felipe M.S.S. (2009). Phylogenetic analysis reveals a high level of speciation in the *Paracoccidioides* genus. Mol. Phylogenet. Evol..

[36] Theodoro R.C., Teixeira M.d.M., Felipe M.S.S., Paduan K.D.S., Ribolla P.M., San-Blas G., Bagagli E. (2012). Genus *Paracoccidioides*: Species recognition and biogeographic aspects. PLoS ONE.

[37] Teixeira M.M., Theodoro R.C., Nino-Vega G., Bagagli E., Felipe M.S.S. (2014). *Paracoccidioides* species complex: Ecology, phylogeny, sexual reproduction, and virulence. PLoS Pathog..

[38] Vandamme P., Pot B., Gillis M., de Vos P., Kersters K., Swings J. (1996). Polyphasic taxonomy, a consensus approach to bacterial systematics. Microbiol. Rev..

[39] Jančič S., Nguyen H.D.T., Frisvad J.C., Zalar P., Schroers H.-J., Seifert K.A., Gunde-Cimerman N. (2015). A taxonomic revision of the *Wallemia sebi* species complex. PLoS ONE.

[40] Quaedvlieg W., Binder M., Groenewald J.Z., Summerell B.A., Carnegie A.J., Burgess T.I., Crous P.W. (2014). Introducing the consolidated species concept to resolve species in the Teratosphaeriaceae. Persoonia.

[41] Muñoz J.F., Farrer R.A., Desjardins C.A., Gallo J.E., Sykes S., Sakthikumar S., Misas E., Whiston E.A., Bagagli E., Soares C.M.A. (2016). Genome diversity, recombination, and virulence across the major lineages of *Paracoccidioides*. mSphere.

[42] De Melo Teixeira M., Cattana M.E., Matute D.R., Muñoz J.F., Arechavala A., Isbell K., Schipper R., Santiso G., Tracogna F., de los Ángeles Sosa M. (2020). Genomic diversity of the human pathogen *Paracoccidioides* across the South American continent. Fungal Genet. Biol..

[43] Cocio T.A., Nascimento E., Kress M.R., Bagagli E., Martinez R. (2020). Characterization of a *Paracoccidioides* spp. strain from southeastern Brazil genotyped as *Paracoccidioides restrepiensis* (PS3) and review of this phylogenetic species. Genet. Mol. Biol..

[44] Cocio T.A., Nascimento E., von Zeska Kress M.R., Bagagli E., Martinez R. (2020). Phylogenetic species of *Paracoccidioides* spp. isolated from clinical and environmental samples in a hyperendemic area of paracoccidioidomycosis in Southeastern Brazil. J. Fungi.

[45] Marques-da-Silva S.H., Rodrigues A.M., de Hoog G.S., Silveira-Gomes F., de Camargo Z.P. (2012). Occurrence of *Paracoccidioides lutzii* in the Amazon region: Description of two cases. Am. J. Trop. Med. Hyg..

[46] Sarmento Tatagiba L., Bridi Pivatto L., Faccini-Martínez Á.A., Mendes Peçanha P., Grão Velloso T.R., Gonçalves S.S., Messias Rodrigues A., Pires Camargo Z., Falqueto A. (2018). A case of paracoccidioidomycosis due to *Paracoccidioides lutzii* presenting sarcoid-like form. Med. Mycol. Case Rep..

[47] Teixeira M.M., Theodoro R.C., Oliveira F.F., Machado G.C., Hahn R.C., Bagagli E., San-Blas G., Felipe M.S. (2014). *Paracoccidioides lutzii* sp. nov.: Biological and clinical implications. Med. Mycol..

[48] Blotta M.H., Camargo Z.P. (1993). Immunological response to cell-free antigens of *Paracoccidioides brasiliensis*: Relationship with clinical forms of paracoccidioidomycosis. J. Clin. Microbiol..

[49] Ohyama K., Kuroda N., Makowski G.S. (2013). Immune Complexome Analysis. Advances in Clinical Chemistry.

[50] Ballesté R.N., Cobo F. (2018). Proteomics: Technology and Applications. The Use of Mass Spectrometry Technology (MALDI-TOF) in Clinical Microbiology.

[51] Haas G., Karaali G., Ebermayer K., Metzger W.G., Lamer S., Zimny-Arndt U., Diescher S., Goebel U.B., Vogt K., Roznowski A.B. (2002). Immunoproteomics of *Helicobacter pylori* infection and relation to gastric disease. Proteomics.

[52] Gautam P., Sundaram C.S., Madan T., Gade W.N., Shah A., Sirdeshmukh R., Sarma P.U. (2007). Identification of novel allergens of *Aspergillus fumigatus* using immunoproteomics approach. Clin. Exp. Allergy.

[53] Costa M.M., Andrade H.M., Bartholomeu D.C., Freitas L.M., Pires S.F., Chapeaurouge A.D., Perales J., Ferreira A.T., Giusta M.S., Melo M.N. (2011). Analysis of *Leishmania chagasi* by 2-D difference gel eleTrop.horesis (2-D DIGE) and immunoproteomic: Identification of novel candidate antigens for diagnostic tests and vaccine. J. Proteome Res..

[54] Do Amaral C.C., Fernandes G.F., Rodrigues A.M., Burger E., de Camargo Z.P. (2019). Proteomic analysis of *Paracoccidioides brasiliensis* complex isolates: Correlation of the levels of differentially expressed proteins with in vivo virulence. PLoS ONE.

[55] Roberto T.N., Rodrigues A.M., Hahn R.C., de Camargo Z.P. (2016). Identifying *Paracoccidioides* phylogenetic species by PCR-RFLP of the alpha-tubulin gene. Med. Mycol..

[56] Brummer E., Restrepo A., Hanson L.H., Stevens D.A. (1990). Virulence of *Paracoccidiodes brasiliensis*: The influence of in vitro passage and storage. Mycopathologia.

[57] Castilho D.G., Chaves A.F., Xander P., Zelanis A., Kitano E.S., Serrano S.M., Tashima A.K., Batista W.L. (2014). Exploring potential virulence regulators in *Paracoccidioides brasiliensis* isolates of varying virulence through quantitative proteomics. J. Proteome Res..

[58] Rodrigues A.M., Kubitschek-Barreira P.H., Fernandes G.F., de Almeida S.R., Lopes-Bezerra L.M., de Camargo Z.P. (2015). Immunoproteomic analysis reveals a convergent humoral response signature in the *Sporothrix schenckii* complex. J. Proteom..

[59] Missall T.A., Pusateri M.E., Donlin M.J., Chambers K.T., Corbett J.A., Lodge J.K. (2006). Posttranslational, translational, and transcriptional responses to nitric oxide stress in *Cryptococcus neoformans*: Implications for virulence. Eukaryot. Cell.

[60] Bradford M.M. (1976). A rapid and sensitive method for the quantitation of microgram quantities of protein utilizing the principle of protein-dye binding. Anal. Biochem..

[61] Blum H., Beier H., Gross H.J. (1987). Improved silver staining of plant proteins, RNA and DNA in polyacrylamide gels. Electrophoresis.

[62] Candiano G., Bruschi M., Musante L., Santucci L., Ghiggeri G.M., Carnemolla B., Orecchia P., Zardi L., Righetti P.G. (2004). Blue silver: A very sensitive colloidal Coomassie G-250 staining for proteome analysis. Electrophoresis.

[63] Towbin H., Staehelin T., Gordon J. (1979). ElecTrop.horetic transfer of proteins from polyacrylamide gels to nitrocellulose sheets: Procedure and some applications. Proc. Natl. Acad. Sci. USA.

[64] Della Terra P.P., Rodrigues A.M., Fernandes G.F., Nishikaku A.S., Burger E., de Camargo Z.P. (2017). Exploring virulence and immunogenicity in the emerging pathogen *Sporothrix brasiliensis*. PLoS Negl. Trop. Dis..

[65] Munoz J.F., Gallo J.E., Misas E., Priest M., Imamovic A., Young S., Zeng Q., Clay O.K., McEwen J.G., Cuomo C.A. (2014). Genome update of the dimorphic human pathogenic fungi causing paracoccidioidomycosis. PLoS Negl. Trop. Dis..

[66] Szklarczyk D., Gable A.L., Lyon D., Junge A., Wyder S., Huerta-Cepas J., Simonovic M., Doncheva N.T., Morris J.H., Bork P. (2019). STRING v11: Protein-protein association networks with increased coverage, supporting functional discovery in genome-wide experimental datasets. Nucleic Acids Res..

[67] Kanehisa M., Goto S., Kawashima S., Okuno Y., Hattori M. (2004). The KEGG resource for deciphering the genome. Nucleic Acids Res..

[68] Darzi Y., Letunic I., Bork P., Yamada T. (2018). iPath3.0: Interactive pathways explorer v3. Nucleic Acids Res..

[69] Horton P., Park K.-J., Obayashi T., Fujita N., Harada H., Adams-Collier C.J., Nakai K. (2007). WoLF PSORT: Protein localization predictor. Nucleic Acids Res..

[70] Da Fonseca C.A., Jesuino R.S.A., Felipe M.S.S., Cunha D.A., Brito W.A., Soares C.M.A. (2001). Two-dimensional elecTrop.horesis and characterization of antigens from *Paracoccidioides brasiliensis*. Microbes Infect..

[71] Moreira A.L.E., Oliveira M.A.P., Silva L.O.H.S., Inácio M.M., Bailão A.M., Parente-Rocha J.A., Cruz-Leite V.R.M., Paccez J.D., de Almeida Soares C.M., Weber S.S. (2020). Immunoproteomic approach of extracellular antigens from *Paracoccidioides* species reveals exclusive B-Cell epitopes. Front. Microbiol..

[72] Saha S., Raghava G.P. (2006). Prediction of continuous B-cell epitopes in an antigen using recurrent neural network. Proteins.

[73] Jespersen M.C., Peters B., Nielsen M., Marcatili P. (2017). BepiPred-2.0: Improving sequence-based B-cell epitope prediction using conformational epitopes. Nucleic Acids Res..

[74] Magnan C.N., Zeller M., Kayala M.A., Vigil A., Randall A., Felgner P.L., Baldi P. (2010). High-throughput prediction of protein antigenicity using protein microarray data. Bioinformatics.

[75] Doytchinova I.A., Flower D.R. (2007). VaxiJen: A server for prediction of protective antigens, tumour antigens and subunit vaccines. BMC Bioinform..

[76] Ashkenazy H., Abadi S., Martz E., Chay O., Mayrose I., Pupko T., Ben-Tal N. (2016). ConSurf 2016: An improved methodology to estimate and visualize evolutionary conservation in macromolecules. Nucleic Acids Res..

[77] Katoh K., Standley D.M. (2013). MAFFT multiple sequence alignment software version 7: Improvements in performance and usability. Mol. Biol. Evol..

[78] Suzek B.E., Wang Y., Huang H., McGarvey P.B., Wu C.H., the UniProt C. (2015). UniRef clusters: A comprehensive and scalable alternative for improving sequence similarity searches. Bioinformatics.

[79] Finn R.D., Clements J., Eddy S.R. (2011). HMMER web server: Interactive sequence similarity searching. Nucleic Acids Res..

[80] Källberg M., Wang H., Wang S., Peng J., Wang Z., Lu H., Xu J. (2012). Template-based protein structure modeling using the RaptorX web server. Nat. Protoc..

[81] Laskowski R.A., MacArthur M.W., Moss D.S., Thornton J.M. (1993). PROCHECK: A program to check the stereochemical quality of protein structures. J. Appl. Crystallogr..

[82] Sweredoski M.J., Baldi P. (2009). COBEpro: A novel system for predicting continuous B-cell epitopes. Protein Eng. Des. Sel..

[83] Wedekind J.E., Poyner R.R., Reed G.H., Rayment I. (1994). Chelation of Serine 39 to Mg^2+^ latches a gate at the active site of enolase: Structure of the Bis(Mg^2+^) Complex of yeast enolase and the intermediate analog phosphonoacetohydroxamate at 2.1-.ANG. resolution. Biochemistry.

[84] Berman H.M., Westbrook J., Feng Z., Gilliland G., Bhat T.N., Weissig H., Shindyalov I.N., Bourne P.E. (2000). The Protein Data Bank. Nucleic Acids Res..

[85] Queiroz Junior Lde P., de Camargo Z.P., Tadano T., Rodrigues A.M., Takarara D.T., Gegembauer G., Araujo L.M., Hahn R.C. (2014). Serological and antigenic profiles of clinical isolates of *Paracoccidioides* spp. from Central Western Brazil. Mycoses.

[86] Batista J., de Camargo Z.P., Fernandes G.F., Vicentini A.P., Fontes C.J.F., Hahn R.C. (2010). Is the geographical origin of a *Paracoccidioides brasiliensis* isolate important for antigen production for regional diagnosis of paracoccidioidomycosis?. Mycoses.

[87] Assato P.A., da Silva Jde F., de Oliveira H.C., Marcos C.M., Rossi D., Valentini S.R., Mendes-Giannini M.J., Zanelli C.F., Fusco-Almeida A.M. (2015). Functional analysis of *Paracoccidioides brasiliensis* 14-3-3 adhesin expressed in *Saccharomyces cerevisiae*. BMC Microbiol..

[88] Longo L.V., Nakayasu E.S., Pires J.H., Gazos-Lopes F., Vallejo M.C., Sobreira T.J., Almeida I.C., Puccia R. (2015). Characterization of lipids and proteins associated to the cell wall of the acapsular mutant *Cryptococcus neoformans* cap 67. J. Eukaryot. Microbiol..

[89] Silva L.D.C., Tauhata S.B.F., Baeza L.C., de Oliveira C.M.A., Kato L., Borges C.L., de Almeida Soares C.M., Pereira M. (2018). Argentilactone molecular targets in *Paracoccidioides brasiliensis* identified by chemoproteomics. Antimicrob. Agents Chemother..

[90] Vallejo M.C., Nakayasu E.S., Matsuo A.L., Sobreira T.J., Longo L.V., Ganiko L., Almeida I.C., Puccia R. (2012). Vesicle and vesicle-free extracellular proteome of *Paracoccidioides brasiliensis*: Comparative analysis with other pathogenic fungi. J. Proteome Res..

[91] Araújo D.S., Pereira M., Portis I.G., Dos Santos Junior A.C.M., Fontes W., de Sousa M.V., Assunção L.D.P., Baeza L.C., Bailão A.M., Ricart C.A.O. (2019). Metabolic peculiarities of *Paracoccidioides* brasiliensis dimorphism as demonstrated by iTRAQ labeling proteomics. Front. Microbiol..

[92] Baeza L.C., da Mata F.R., Pigosso L.L., Pereira M., de Souza G., Coelho A.S.G., de Almeida Soares C.M. (2017). Differential metabolism of a two-carbon substrate by members of the *Paracoccidioides* genus. Front. Microbiol..

[93] Pigosso L.L., Parente A.F., Coelho A.S., Silva L.P., Borges C.L., Bailao A.M., Soares C.M. (2013). Comparative proteomics in the genus *Paracoccidioides*. Fungal Genet. Biol..

[94] Wang L., Wu X., Gao W., Zhao M., Zhang J., Huang C. (2017). Differential expression patterns of *Pleurotus ostreatus* catalase genes during developmental stages and under heat stress. Genes.

[95] Longo L.V.G., Breyer C.A., Novaes G.M., Gegembauer G., Leitão N.P., Octaviano C.E., Toyama M.H., de Oliveira M.A., Puccia R. (2020). The human pathogen *Paracoccidioides brasiliensis* has a unique 1-cys peroxiredoxin that localizes both intracellularly and at the cell surface. Front. Cell Infect. Microbiol..

[96] Fradin C., De Groot P., MacCallum D., Schaller M., Klis F., Odds F.C., Hube B. (2005). Granulocytes govern the transcriptional response, morphology and proliferation of *Candida albicans* in human blood. Mol. Microbiol..

[97] Lorenz M.C., Bender J.A., Fink G.R. (2004). Transcriptional response of *Candida albicans* upon internalization by macrophages. Eukaryot. Cell.

[98] Barelle C.J., Priest C.L., Maccallum D.M., Gow N.A., Odds F.C., Brown A.J. (2006). Niche-specific regulation of central metabolic pathways in a fungal pathogen. Cell Microbiol..

[99] Chen Y., Toffaletti D.L., Tenor J.L., Litvintseva A.P., Fang C., Mitchell T.G., McDonald T.R., Nielsen K., Boulware D.R., Bicanic T. (2014). The *Cryptococcus neoformans* transcriptome at the site of human meningitis. mBio.

[100] Ries L.N.A., Beattie S., Cramer R.A., Goldman G.H. (2018). Overview of carbon and nitrogen catabolite metabolism in the virulence of human pathogenic fungi. Mol. Microbiol..

[101] Hibbett D.S., Binder M., Bischoff J.F., Blackwell M., Cannon P.F., Eriksson O.E., Huhndorf S., James T., Kirk P.M., Lücking R. (2007). A higher-level phylogenetic classification of the Fungi. Mycol. Res..

[102] Wisecaver J.H., Slot J.C., Rokas A. (2014). The evolution of fungal metabolic pathways. PLoS Genet..

[103] Shen Q., Rappleye C.A. (2020). Living within the macrophage: Dimorphic fungal pathogen intracellular metabolism. Front. Cell Infect. Microbiol..

[104] Milhomem Cruz-Leite V.R., Salem-Izacc S.M., Novaes E., Neves B.J., de Almeida Brito W., O’Hara Souza Silva L., Paccez J.D., Parente-Rocha J.A., Pereira M., Maria de Almeida Soares C. (2020). Nitrogen catabolite repression in members of *Paracoccidioides* complex. Microb. Pathog..

[105] De Sousa Lima P., Casaletti L., Bailão A.M., de Vasconcelos A.T.R., da Rocha Fernandes G., de Almeida Soares C.M. (2014). Transcriptional and proteomic responses to carbon starvation in *Paracoccidioides*. PLoS Negl. Trop. Dis..

[106] Lacerda Pigosso L., Baeza L.C., Vieira Tomazett M., Batista Rodrigues Faleiro M., Brianezi Dignani de Moura V.M., Melo Bailao A., Borges C.L., Alves Parente Rocha J., Rocha Fernandes G., Gauthier G.M. (2017). *Paracoccidioides brasiliensis* presents metabolic reprogramming and secretes a serine proteinase during murine infection. Virulence.

[107] Brock M. (2009). Fungal metabolism in host niches. Curr. Opin. Microbiol..

[108] Da Silva T.A., Roque-Barreira M.C., Casadevall A., Almeida F. (2016). Extracellular vesicles from *Paracoccidioides brasiliensis* induced M1 polarization in vitro. Sci. Rep..

[109] Barbosa M.S., Báo S.N., Andreotti P.F., de Faria F.P., Felipe M.S., dos Santos Feitosa L., Mendes-Giannini M.J., Soares C.M. (2006). Glyceraldehyde-3-phosphate dehydrogenase of *Paracoccidioides brasiliensis* is a cell surface protein involved in fungal adhesion to extracellular matrix proteins and interaction with cells. Infect. Immun..

[110] Pereira L.A., Báo S.N., Barbosa M.S., da Silva J.L., Felipe M.S., de Santana J.M., Mendes-Giannini M.J., de Almeida Soares C.M. (2007). Analysis of the *Paracoccidioides brasiliensis* triosephosphate isomerase suggests the potential for adhesin function. FEMS Yeast Res..

[111] Donofrio F.C., Calil A.C.A., Miranda E.T., Almeida A.M.F., Benard G., Soares C.P., Veloso S.N., Soares C.M.d.A., Mendes Giannini M.J.S. (2009). Enolase from *Paracoccidioides brasiliensis*: Isolation and identification as a fibronectin-binding protein. J. Med. Microbiol..

[112] Nogueira S.V., Fonseca F.L., Rodrigues M.L., Mundodi V., Abi-Chacra E.A., Winters M.S., Alderete J.F., de Almeida Soares C.M. (2010). *Paracoccidioides brasiliensis* enolase is a surface protein that binds plasminogen and mediates interaction of yeast forms with host cells. Infect. Immun..

[113] De Oliveira H.C., da Silva J.d.F., Scorzoni L., Marcos C.M., Rossi S.A., de Paula e Silva A.C.A., Assato P.A., da Silva R.A.M., Fusco-Almeida A.M., Mendes-Giannini M.J.S. (2015). Importance of adhesins in virulence of *Paracoccidioides* spp.. Front. Microbiol..

[114] Chaves E.G., Weber S.S., Bao S.N., Pereira L.A., Bailao A.M., Borges C.L., Soares C.M. (2015). Analysis of *Paracoccidioides* secreted proteins reveals fructose 1,6-bisphosphate aldolase as a plasminogen-binding protein. BMC Microbiol..

[115] Andreotti P.F., Monteiro da Silva J.L., Bailão A.M., Soares C.M.d.A., Benard G., Soares C.P., Mendes-Giannini M.J.S. (2005). Isolation and partial characterization of a 30 kDa adhesin from *Paracoccidioides brasiliensis*. Microbes Infect..

[116] Longo L.V.G., da Cunha J.P.C., Sobreira T.J.P., Puccia R. (2014). Proteome of cell wall-extracts from pathogenic *Paracoccidioides brasiliensis*: Comparison among morphological phases, isolates, and reported fungal extracellular vesicle proteins. EuPA Open Proteom..

[117] Jeffery C.J. (2018). Protein moonlighting: What is it, and why is it important?. Philos. Trans. R. Soc. B.

[118] Rodrigues M.L., Nakayasu E.S., Oliveira D.L., Nimrichter L., Nosanchuk J.D., Almeida I.C., Casadevall A. (2008). Extracellular vesicles produced by *Cryptococcus neoformans* contain protein components associated with virulence. Eukaryot. Cell.

[119] Albuquerque P.C., Nakayasu E.S., Rodrigues M.L., Frases S., Casadevall A., Zancope-Oliveira R.M., Almeida I.C., Nosanchuk J.D. (2008). Vesicular transport in *Histoplasma capsulatum*: An effective mechanism for trans-cell wall transfer of proteins and lipids in ascomycetes. Cell Microbiol..

[120] Oliveira D.L., Nakayasu E.S., Joffe L.S., Guimarães A.J., Sobreira T.J.P., Nosanchuk J.D., Cordero R.J.B., Frases S., Casadevall A., Almeida I.C. (2010). Characterization of yeast extracellular vesicles: Evidence for the participation of different pathways of cellular traffic in vesicle biogenesis. PLoS ONE.

[121] Da Silva Jde F., de Oliveira H.C., Marcos C.M., da Silva R.A., da Costa T.A., Calich V.L., Almeida A.M., Mendes-Giannini M.J. (2013). *Paracoccidoides brasiliensis* 30 kDa adhesin: Identification as a 14-3-3 protein, cloning and subcellular localization in infection models. PLoS ONE.

[122] Tomazett M.V., Baeza L.C., Paccez J.D., Parente-Rocha J.A., Ribeiro-Dias F., de Almeida Soares C.M. (2019). Identification and characterization of *Paracoccidioides lutzii* proteins interacting with macrophages. Microbes Infect..

[123] De Oliveira H.C., Castelli R.F., Reis F.C.G., Rizzo J., Rodrigues M.L. (2020). Pathogenic delivery: The biological roles of Cryptococcal extracellular vesicles. Pathogens.

[124] Champer J., Ito J.I., Clemons K.V., Stevens D.A., Kalkum M. (2016). Proteomic analysis of pathogenic fungi reveals highly expressed conserved cell wall proteins. J. Fungi.

[125] Lee P.Y., Gam L.H., Yong V.C., Rosli R., Ng K.P., Chong P.P. (2014). Identification of immunogenic proteins of *Candida parapsilosis* by serological proteome analysis. J. Appl. Microbiol..

[126] Puccia R., Vallejo M., Matsuo A., Longo L. (2011). The *Paracoccidioides* cell wall: Past and present layers toward understanding interaction with the host. Front. Microbiol..

[127] Cisalpino P.S., Puccia R., Yamauchi L.M., Cano M.I.N., da Silveira J.F., Travassos L.R. (1996). Cloning, characterization, and epitope expression of the major diagnostic antigen of *Paracoccidioides brasiliensis*. J. Biol. Chem..

[128] Mendes-Giannini M.J., Andreotti P.F., Vincenzi L.R., da Silva J.L., Lenzi H.L., Benard G., Zancope-Oliveira R., de Matos Guedes H.L., Soares C.P. (2006). Binding of extracellular matrix proteins to *Paracoccidioides brasiliensis*. Microbes Infect..

[129] Puccia R., Travassos L.R., Rodrigues E.G., Camona A.K., Oliveira M.C., Juliano L., Maresca B., Kobayashi G.S. (1994). Purification of the specific exocellular antigen gp43 from *Paracoccidioides brasiliensis*: Immunological and proteolytic activities. Molecular Biology of Pathogenic Fungi: A Laboratory Manual.

[130] De Camargo Z.P., Berzaghi R., Amaral C.C., Silva S.H.M. (2003). Simplified method for producing *Paracoccidioides brasiliensis* exoantigens for use in immunodiffusion tests. Med. Mycol..

[131] Berzaghi R., da Silva S.H., de Camargo Z.P. (2005). Variable gp43 secretion by *Paracoccidioides brasiliensis* clones obtained by two different culture methods. J. Clin. Microbiol..

[132] Magdeldin S., Enany S., Yoshida Y., Xu B., Zhang Y., Zureena Z., Lokamani I., Yaoita E., Yamamoto T. (2014). Basics and recent advances of two dimensional- polyacrylamide gel elecTrop.horesis. Clin. Proteom..

[133] Leitão N.P., Vallejo M.C., Conceição P.M., Camargo Z.P., Hahn R., Puccia R. (2014). *Paracoccidioides lutzii* Plp43 is an active glucanase with partial antigenic identity with *P. brasiliensis* gp43. PLoS Negl. Trop. Dis..

[134] Marcos C.M., de Fátima da Silva J., de Oliveira H.C., Moraes da Silva R.A., Mendes-Giannini M.J.S., Fusco-Almeida A.M. (2012). Surface-expressed enolase contributes to the adhesion of *Paracoccidioides brasiliensis* to host cells. FEMS Yeast Res..

[135] Areitio M., Martin-Vicente A., Arbizu A., Antoran A., Aparicio-Fernandez L., Buldain I., Martin-Souto L., Rementeria A., Capilla J., Hernando F.L. (2020). Identification of *Mucor circinelloides* antigens recognized by sera from immunocompromised infected mice. Rev. Iberoam. Micol..

[136] Yadav R.K., Shukla P.K. (2019). A novel monoclonal antibody against enolase antigen of *Aspergillus fumigatus* protects experimental aspergillosis in mice. FEMS Microbiol. Lett..

[137] Santos L.P.A., Assunção L.d.P., Lima P.d.S., Tristão G.B., Brock M., Borges C.L., Silva-Bailão M.G., Soares C.M.d.A., Bailão A.M. (2020). Propionate metabolism in a human pathogenic fungus: Proteomic and biochemical analyses. IMA Fungus.

[138] Lapaille M., Thiry M., Perez E., González-Halphen D., Remacle C., Cardol P. (2010). Loss of mitochondrial ATP synthase subunit beta (Atp2) alters mitochondrial and chloroplastic function and morphology in *Chlamydomonas*. Biochim. Biophys. Acta Biomembr..

[139] Warner H.R., Duncan B.K., Garrett C., Neuhard J. (1981). Synthesis and metabolism of uracil-containing deoxyribonucleic acid in *Escherichia coli*. J. Bacteriol..

[140] Vértessy B.G., Tóth J. (2009). Keeping uracil out of DNA: Physiological role, structure and catalytic mechanism of dUTPases. Acc. Chem. Res..

[141] Strzalka W., Ziemienowicz A. (2011). Proliferating cell nuclear antigen (PCNA): A key factor in DNA replication and cell cycle regulation. Ann. Bot..

[142] Bravo R., Frank R., Blundell P.A., Macdonald-Bravo H. (1987). Cyclin/PCNA is the auxiliary protein of DNA polymerase-delta. Nature.

[143] Desjardins C.A., Champion M.D., Holder J.W., Muszewska A., Goldberg J., Bailao A.M., Brigido M.M., Ferreira M.E., Garcia A.M., Grynberg M. (2011). Comparative genomic analysis of human fungal pathogens causing paracoccidioidomycosis. PLoS Genet..

[144] Silva P.F., Novaes E., Pereira M., Soares C.M., Borges C.L., Salem-Izacc S.M. (2015). In silico characterization of hypothetical proteins from *Paracoccidioides lutzii*. Genet. Mol. Res..

[145] Keller N.P. (2019). Fungal secondary metabolism: Regulation, function and drug discovery. Nat. Rev. Microbiol..

[146] de Oliveira H.C., da Silva J.d.F., Matsumoto M.T., Marcos C.M., Peres da Silva R., Moraes da Silva R.A., Labate M.T.V., Labate C.A., Fusco Almeida A.M., Mendes Giannini M.J.S. (2014). Alterations of protein expression in conditions of copper-deprivation for *Paracoccidioides lutzii* in the presence of extracellular matrix components. BMC Microbiol..

[147] Mayer M.P., Bukau B. (2005). Hsp70 chaperones: Cellular functions and molecular mechanism. Cell Mol. Life Sci..

[148] Hartl F.U., Bracher A., Hayer-Hartl M. (2011). Molecular chaperones in protein folding and proteostasis. Nature.

[149] Navarro M.V., Chaves A.F.A., Castilho D.G., Casula I., Calado J.C.P., Conceição P.M., Iwai L.K., de Castro B.F., Batista W.L. (2020). Effect of nitrosative stress on the S-nitroso-proteome of *Paracoccidioides brasiliensis*. Front. Microbiol..

[150] Burnie J.P., Carter T.L., Hodgetts S.J., Matthews R.C. (2006). Fungal heat-shock proteins in human disease. FEMS Microbiol. Rev..

[151] Izacc S.M., Gomez F.J., Jesuino R.S., Fonseca C.A., Felipe M.S., Deepe G.S., Soares C.M. (2001). Molecular cloning, characterization and expression of the heat shock protein 60 gene from the human pathogenic fungus *Paracoccidioides brasiliensis*. Med. Mycol..

[152] Thomaz L., Nosanchuk J.D., Rossi D.C., Travassos L.R., Taborda C.P. (2014). Monoclonal antibodies to heat shock protein 60 induce a protective immune response against experimental *Paracoccidioides lutzii*. Microbes Infect..

[153] Tiwari S., Thakur R., Shankar J. (2015). Role of heat-shock proteins in cellular function and in the biology of fungi. Biotechnol. Res. Int..

[154] Léveillard T., Aït-Ali N. (2017). Cell signaling with extracellular thioredoxin and thioredoxin-like proteins: Insight into their mechanisms of action. Oxidative Med. Cell Longev..

[155] De Oliveira A.R., Oliveira L.N., Chaves E.G.A., Weber S.S., Bailão A.M., Parente-Rocha J.A., Baeza L.C., de Almeida Soares C.M., Borges C.L. (2018). Characterization of extracellular proteins in members of the *Paracoccidioides* complex. Fungal Biol..

[156] Krahulec S., Armao G.C., Klimacek M., Nidetzky B. (2011). Enzymes of mannitol metabolism in the human pathogenic fungus *Aspergillus fumigatus*--kinetic properties of mannitol-1-phosphate 5-dehydrogenase and mannitol 2-dehydrogenase, and their physiological implications. FEBS J..

[157] Jennings D.B., Ehrenshaft M., Pharr D.M., Williamson J.D. (1998). Roles for mannitol and mannitol dehydrogenase in active oxygen-mediated plant defense. Proc. Natl. Acad. Sci. USA.

[158] Upadhyay R., Meena M., Prasad V., Zehra A., Gupta V. (2015). Mannitol metabolism during pathogenic fungal–host interactions under stressed conditions. Front. Microbiol..

[159] Malito E., Alfieri A., Fraaije M.W., Mattevi A. (2004). Crystal structure of a Baeyer–Villiger monooxygenase. Proc. Natl. Acad. Sci. USA.

[160] Vasiliou V., Ross D., Nebert D.W. (2006). Update of the NAD(P)H:quinone oxidoreductase (NQO) gene family. Hum. Genom..

[161] Minerdi D., Zgrablic I., Sadeghi S.J., Gilardi G. (2012). Identification of a novel Baeyer-Villiger monooxygenase from *Acinetobacter radioresistens*: Close relationship to the *Mycobacterium tuberculosis* prodrug activator EtaA. Microb. Biotechnol..

[162] Alkan N., Fluhr R., Sherman A., Prusky D. (2008). Role of ammonia secretion and ph modulation on pathogenicity of *Colletotrichum coccodes* on tomato fruit. Mol. Plant Microbe Interact..

[163] Alkan N., Davydov O., Sagi M., Fluhr R., Prusky D. (2009). Ammonium secretion by *Colletotrichum coccodes* activates host NADPH oxidase activity enhancing host cell death and fungal virulence in tomato fruits. Mol. Plant Microbe Interact..

[164] Kim S.Y., Paeng S.K., Nawkar G.M., Maibam P., Lee E.S., Kim K.S., Lee D.H., Park D.J., Kang S.B., Kim M.R. (2011). The 1-Cys peroxiredoxin, a regulator of seed dormancy, functions as a molecular chaperone under oxidative stress conditions. Plant Sci..

[165] Rocha M.C., de Godoy K.F., Bannitz-Fernandes R., Fabri J.H.T.M., Barbosa M.M.F., de Castro P.A., Almeida F., Goldman G.H., da Cunha A.F., Netto L.E.S. (2018). Analyses of the three 1-Cys peroxiredoxins from *Aspergillus fumigatus* reveal that cytosolic Prx1 is central to H_2_O_2_ metabolism and virulence. Sci. Rep..

[166] Tanaka K. (2009). The proteasome: Overview of structure and functions. Proc. Jpn. Acad. Ser. B Phys. Biol. Sci..

[167] Potocnakova L., Bhide M., Pulzova L.B. (2016). An introduction to B-cell epitope mapping and *in silico* epitope prediction. J. Immunol. Res..

[168] Tomar N., De R.K. (2010). Immunoinformatics: An integrated scenario. Immunology.

[169] Larsen J.E., Lund O., Nielsen M. (2006). Improved method for predicting linear B-cell epitopes. Immunome Res..

[170] El-Manzalawy Y., Dobbs D., Honavar V. (2008). Predicting linear B-cell epitopes using string kernels. J. Mol. Recognit..

[171] Yao B., Zhang L., Liang S., Zhang C. (2012). SVMTriP: A method to predict antigenic epitopes using support vector machine to integrate tri-peptide similarity and propensity. PLoS ONE.

[172] Portes L.D.S., Kioshima E.S., de Camargo Z.P., Batista W.L., Xander P. (2017). Subtractive phage display selection for screening and identification of peptide sequences with potential use in serodiagnosis of paracoccidioidomycosis caused by *Paracoccidioides brasiliensis*. Lett. Appl. Microbiol..

[173] Caldini C.P., Xander P., Kioshima É.S., Bachi A.L., de Camargo Z.P., Mariano M., Lopes J.D. (2012). Synthetic peptides mimic gp75 from *Paracoccidioides brasiliensis* in the diagnosis of paracoccidioidomycosis. Mycopathologia.

[174] Mendes-Giannini M.J., Camargo M.E., Lacaz C.S., Ferreira A.W. (1984). Immunoenzymatic absorption test for serodiagnosis of paracoccidioidomycosis. J. Clin. Microbiol..

[175] Ferreira-da-Cruz M.F., Francesconi-do-Vale A.C., Espinera M.C., Wanke B., Galvao-Castro B. (1990). Study of antibodies in paracoccidioidomycosis: Follow-up of patients during and after treatment. Med. Mycol..

[176] Camargo Z.P., Taborda C.P., Rodrigues E.G., Travassos L.R. (1991). The use of cell-free antigens of *Paracoccidioides brasiliensis* in serological tests. Med. Mycol..

[177] Fernandes G.F., Deps P., Tomimori-Yamashita J., Camargo Z.P. (2004). IgM and IgG antibody response to *Paracoccidioides brasiliensis* in naturally infected wild armadillos (*Dasypus novemcinctus*). Med. Mycol..

[178] Marques-da-Silva S.H., Colombo A.L., Blotta M.H.S.L., Queiroz-Telles F., Balthazar A.B., Lopes J.D., de Camargo Z.P. (2006). Diagnosis of paracoccidioidomycosis by detection of antigen and antibody in bronchoalveolar lavage fluids. Clin. Vaccine Immunol..

[179] Carvalho K.C., Vallejo M.C., Camargo Z.P., Puccia R. (2008). Use of recombinant gp43 isoforms expressed in *Pichia pastoris* for diagnosis of paracoccidioidomycosis. Clin. Vaccine Immunol..

[180] De Macedo P.M., Teixeira M.d.M., Barker B.M., Zancopé-Oliveira R.M., Almeida-Paes R., Francesconi do Valle A.C. (2019). Clinical features and genetic background of the sympatric species *Paracoccidioides brasiliensis* and *Paracoccidioides americana*. PLoS Negl. Trop. Dis..

