# Supplementary material for: Immunoproteomic Analysis Reveals Novel Candidate Antigens for the Diagnosis of Paracoccidioidomycosis Due to Paracoccidioides lutzii
Source: J Fungi (Basel). 2020 Dec 11;6(4):357. doi: 10.3390/jof6040357 (PMC7770604; doi:10.3390/jof6040357)
Supplement: Supplementary file 1 [file jof-06-00357-s001.pdf]

Journal of *Fungi*

Supplementary material

Immunoproteomic Analysis Reveals Novel Candidate Antigens for the Diagnosis of Paracoccidioidomycosis Due to *Paracoccidioides lutzii*

Anderson Messias Rodrigues[1]*, Paula Helena Kubitschek Barreira[2], Breno Gonçalves Pinheiro[1], André Teixeira-Ferreira[3], Rosane Cristine Hahn[4,5], Zoilo Pires de Camargo[1,6]*

1 Laboratory of Emerging Fungal Pathogens, Department of Microbiology, Immunology, and Parasitology, Discipline of Cellular Biology, Federal University of São Paulo (UNIFESP), São Paulo 04023062, Brazil.
2 Department of Cellular Biology, Roberto Alcantara Gomes Institute of Biology, Rio de Janeiro State University (UERJ), Rio de Janeiro, 20511010, Brazil.
3 Department of Physiology and Pharmacodynamics, Toxinology Laboratory, Fiocruz, Rio de Janeiro 21040900, RJ, Brazil.
4 Laboratory of Mycology/Research, Faculty of Medicine, Federal University of Mato Grosso, Cuiabá, Mato Grosso 78060900, Brazil.
5 Federal University of Mato Grosso, Júlio Muller University Hospital, Mato Grosso 78048902, Brazil
6 Department of Medicine, Discipline of Infectious Diseases, Federal University of São Paulo (UNIFESP), São Paulo 04023062, Brazil.
* Correspondence: (ZPC) zpcamargo1@gmail.com; Tel.: +55-1155764551 Ext. 1512. (AMR) amrodrigues@unifesp.br; Tel.: +55-1155764551 Ext. 1540.

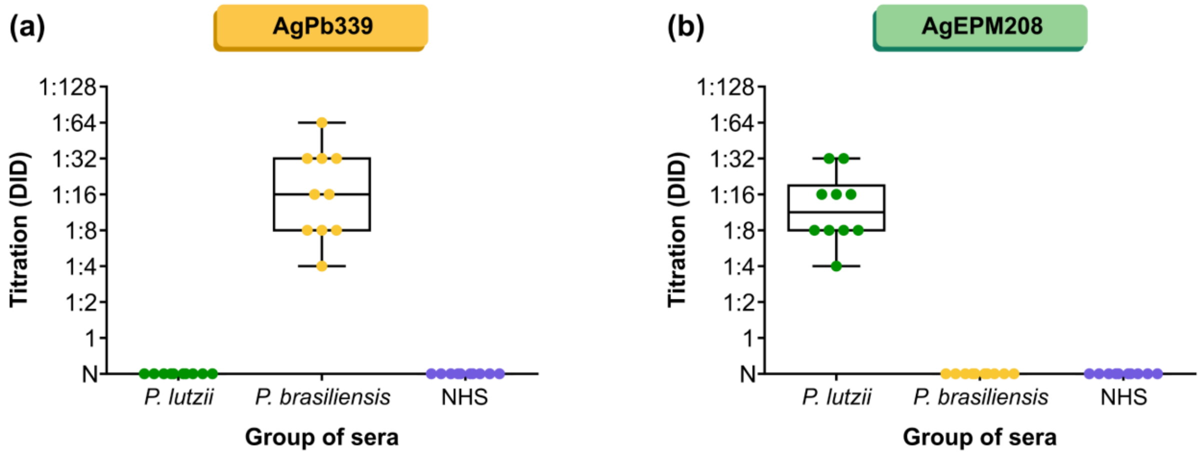

Figure S1. Anti-*Paracoccidioides* antibody titers in reactive sera of patients with paracoccidioidomycosis due to *P. brasiliensis sensu stricto* (S1; n=10) or *P. lutzii* (n=10). NHS: normal human serum (sera from the healthy normal donors; n=10). A quantitative double immunodiffusion assay (DID) was used to establish the titration of each patient's serum using **(a)** the exoantigens from the reference B-339 strain (AgPbB339; PS3) or **(b)** a *P. lutzii* CFA preparation derived from the reference isolate EPM208 as described earlier [1,2]. Each dot represents the result of a single patient. Bars represent the lowest (Min) and the highest (Max) value in each column.

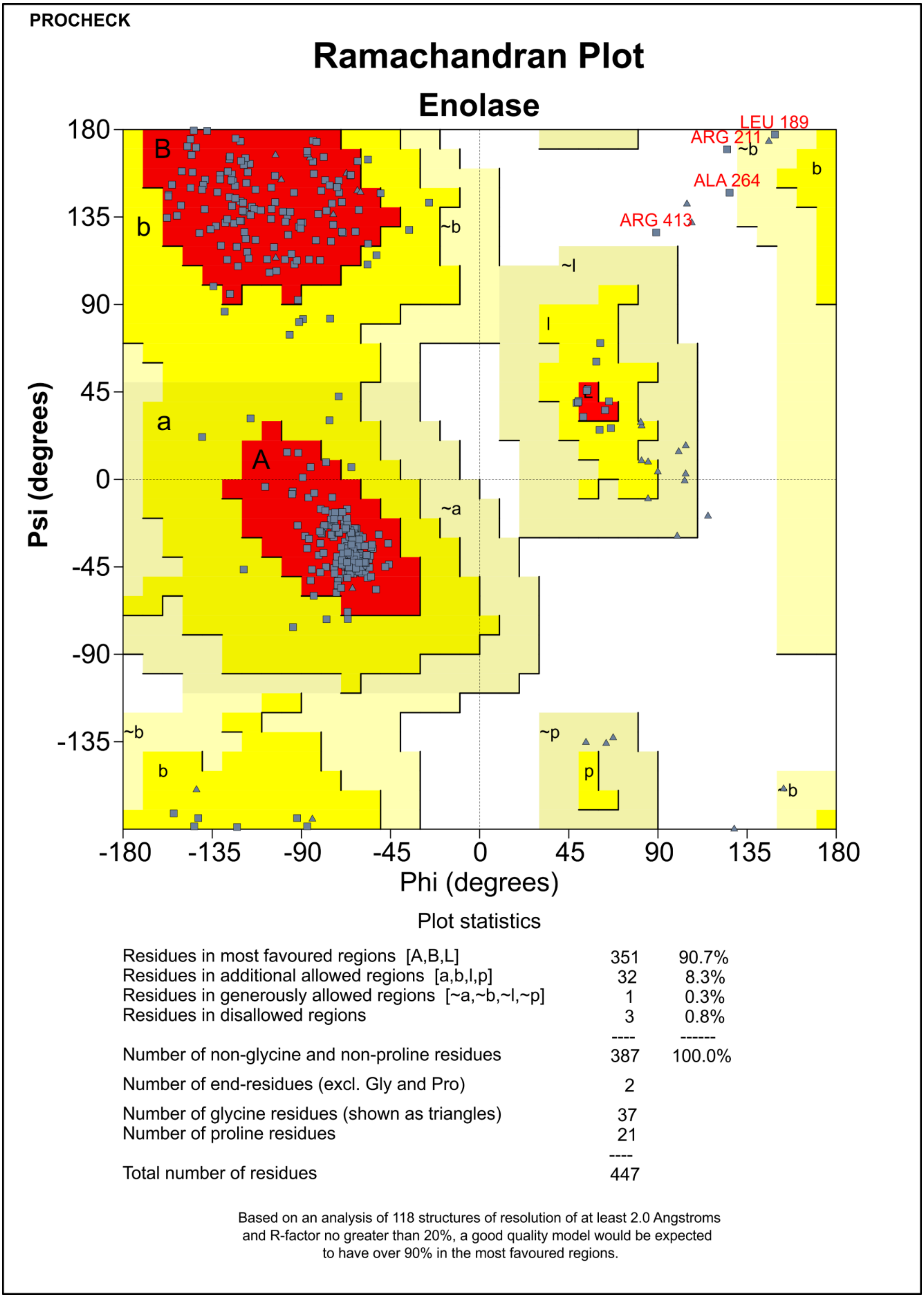

Figure S2. Ramachandran plot generated by PROCHECK validation server [3] showing the stereochemical quality of the *Paracoccidioides lutzii* enolase structure (accession number: XP_015703472).

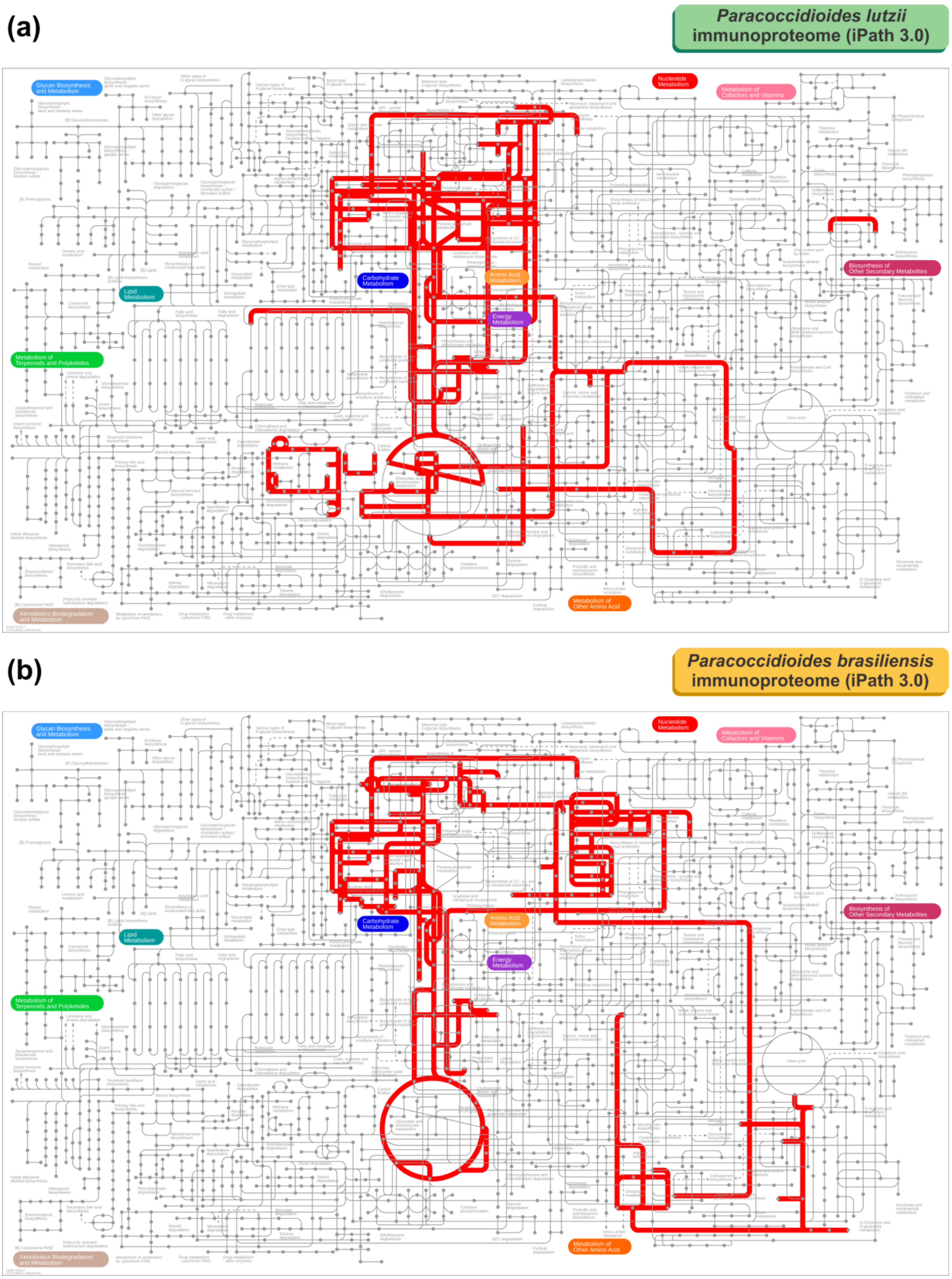

Figure S3. Overview of the metabolic pathways in **(a)** *P. lutzii* and **(b)** *P. brasiliensis* proteomes. The red lines represent proteins (IgG immunoreactive) that were highly enriched, and the boxes represent metabolic pathways. These metabolic maps were visualized and analyzed by the web-application iPath3.0 [4].

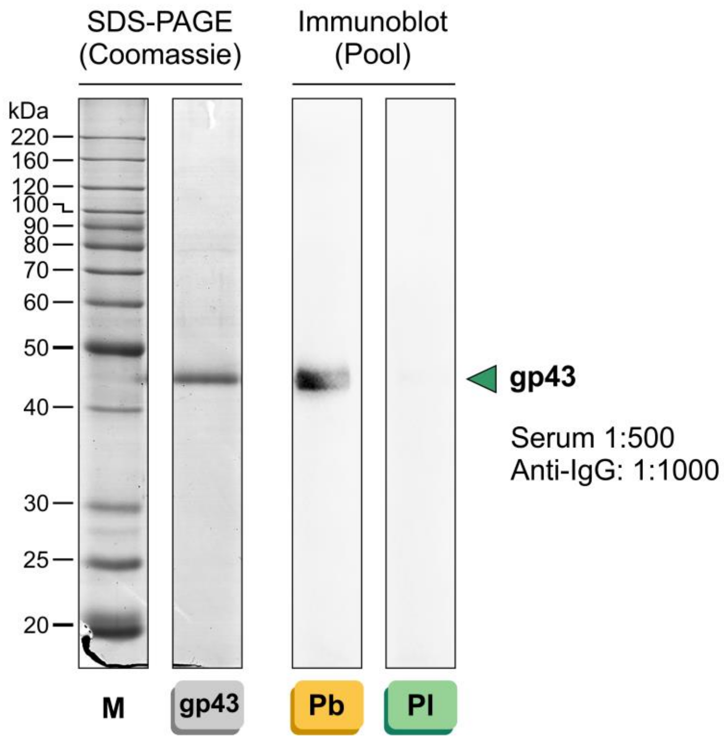

Figure S4. Representative immunoblot of purified gp43 antigen (B-339 strain) using sera from patients with confirmed paracoccidioidomycosis due to *P. brasiliensis s. str.* (Pb, yellow lane) or *P. lutzii* (Pl, green lane). For immunoblotting, gp43 (250 ng) was resolved with SDS-PAGE and transferred onto 0.45 polyvinylidene difluoride (PVDF) membranes (Bio-Rad, USA) at 25 V for 1 h with transfer buffer (25 mM Tris base, 192 mM glycine, 20% methanol; pH 8.3) using a Trans-Blot SD semi-dry transfer cell (Bio-Rad, USA). Membranes were probed individually with primary antibody diluted 1:500 (gold standard pooled human sera) at 25 °C for 2 h. Afterward, the membranes were washed three times with Tris-buffered saline (pH 7.5) containing 0.05% (v/v) Tween 20 (TBST) for 10 min and incubated with horseradish peroxidase (HRP)-conjugated goat anti-human IgG (1:1,000 dilution) for 2 h at room temperature. The membranes were then washed with TBST, and the signal was detected with an enhanced chemiluminescence detection kit (GE Healthcare, Uppsala, Sweden). Immunoblots were imaged in a transilluminator (Uvitec, Cambridge). Alliance software v. 4.7 was used to capture several images at different exposure times, reaching ten shots with a 2 s increment. M: marker.

Journal of
Fungi

Table S1. BepiPred Linear Epitope Prediction 2.0 Results.

We used a high-throughput *in silico* prediction of protein antigenicity to compare linear B-cell epitopes between *P. brasiliensis* (Pb18) and *P. lutzii* (Pb01) immunoreactive proteins. Therefore, protein sequences were retrieved from NCBI and submitted to BepiPred [5] using the default parameters.

Protein name: NAD(P)H: quinone oxidoreductase, type IV

Predicted peptides: XP_015702763 [Pb01]

No.	Start	End	Peptide	Length
1	15	18	FIPA	4
2	58	82	ETLPQEVLDKMHAPGKSSYPVAEPA	25
3	102	103	PG	2
4	114	122	GIWSTGGFW	9
5	136	139	GGGQ	4
6	163	180	KTTFPILANLNEVRGGSP	18
7	188	202	GADGSRQPSALELQL	15

Bepipred Linear Epitope Prediction 2.0

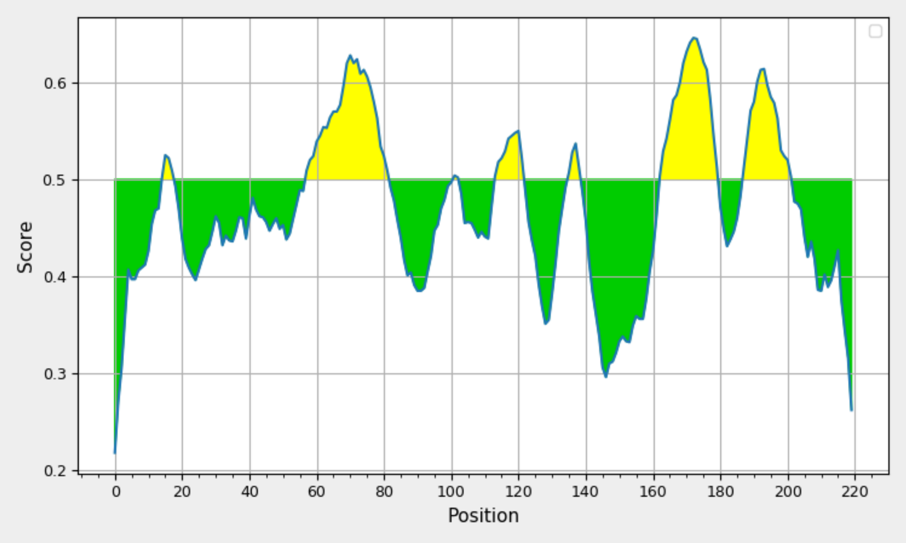

Predicted peptides: XP_010763025 [Pb18]

No.	Start	End	Peptide	Length
1	44	44	I	1
2	46	69	ETLPQEVLDKMHAPGKSSYPVAEP	24
3	105	110	STGGFW	6
4	123	127	PGGGQ	5
5	152	167	TTFPILANLDEVRGGS	16
6	175	189	AGADGSRQPSALELQ	15

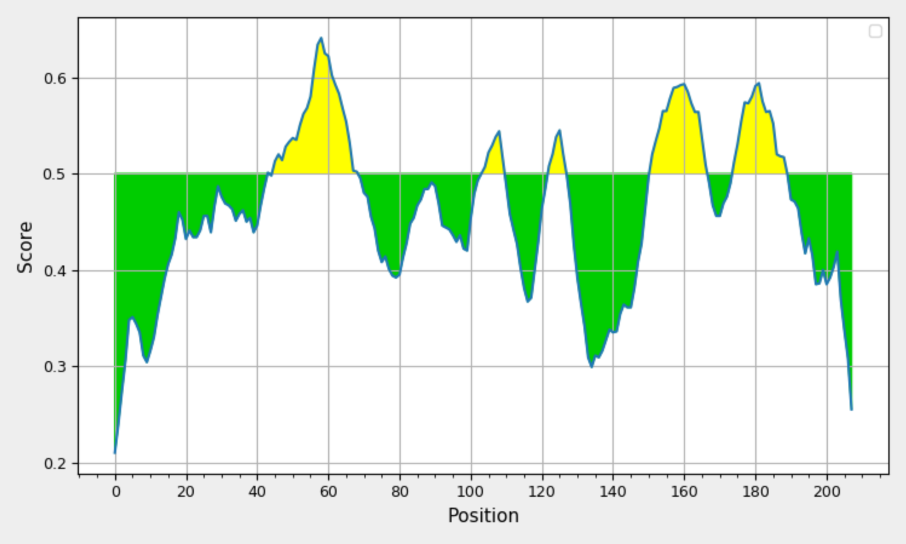

Journal of Fungi

Protein name: Uncharacterized protein (Thioredoxin-like Superfamily)

Predicted peptides: XP_015700719 [Pb01]

No.	Start	End	Peptide	Length
1	6	24	ISRLTLHDPPVYSVAKQFP	19
2	37	84	PSNPPTLRTSQITSKTYHDSKSPI RRPEALPEWRHGDQAHAGIFTPSK	48
3	116	122	SKNPHYR	7
4	134	138	**QPWHP**	5
5	153	157	LAPEK	5
6	169	186	QTEFFDANVVNETRNETY	18
7	198	198	V	1
8	200	201	EA	2
9	203	228	LLKMLAVSDKPDEQGDLNGGNGVTGD	26
10	230	230	K	1
11	233	239	**VKASRLI**	7
12	253	270	**EERTISSRFTAEQWEKWL**	18

Bepipred Linear Epitope Prediction 2.0

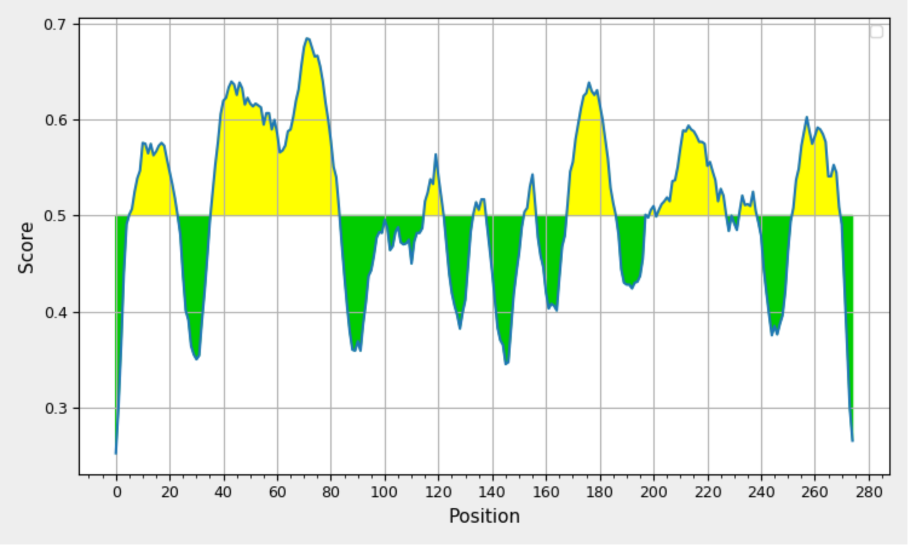

Predicted peptides: XP_010758121 [Pb18]

No.	Start	End	Peptide	Length
1	5	20	PQFAGQKLSLSGGTET	16
2	29	41	LRTQKPDTNPATS	13
3	62	69	SKNAHYRD	8
4	80	84	**QPWHP**	5
5	98	103	KLAPEK	6
6	116	131	TEFFDANVVNETRNET	16
7	144	170	VDEAALLKMLAVSDKPDEQGDLNGGNG	27
8	172	173	TG	2
9	179	185	**VKASRLI**	7
10	199	216	**EERTISSRFTAEQWEKWL**	18

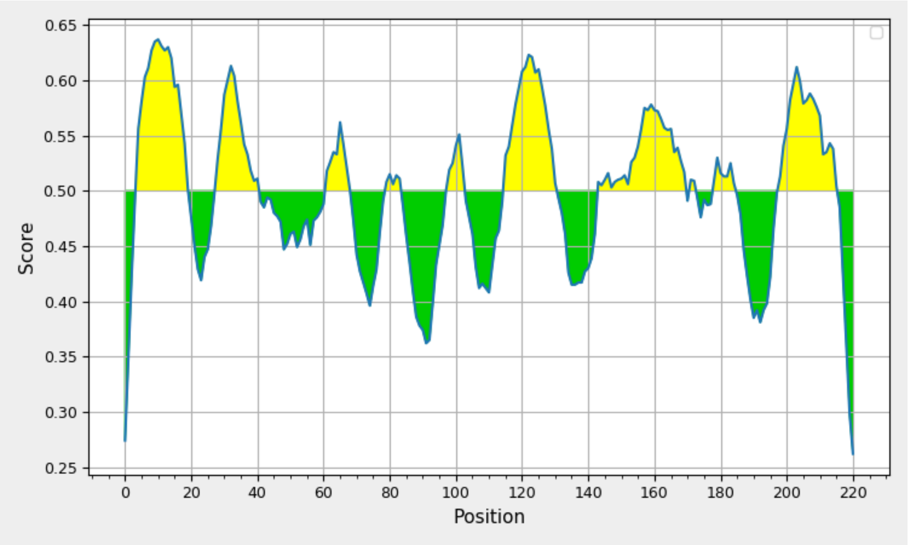

Protein name: Triosephosphate isomerase

Predicted peptides: XP_002795879 [Pb01]

No.	Start	End	Peptide	Length
1	15	19	GTAKS	5
2	31	33	KLD	3
3	69	72	PNGA	4
4	96	107	ERRVLLREDDEF	12
5	130	141	LEEREAGKTIDV	12
6	151	151	E	1
7	155	156	PA	2
8	172	183	GTGKVATTEQAQ	12
9	194	202	NEKISPEAA	9
10	215	217	ENN	3
11	251	254	GIPH	4

Bepipred Linear Epitope Prediction 2.0

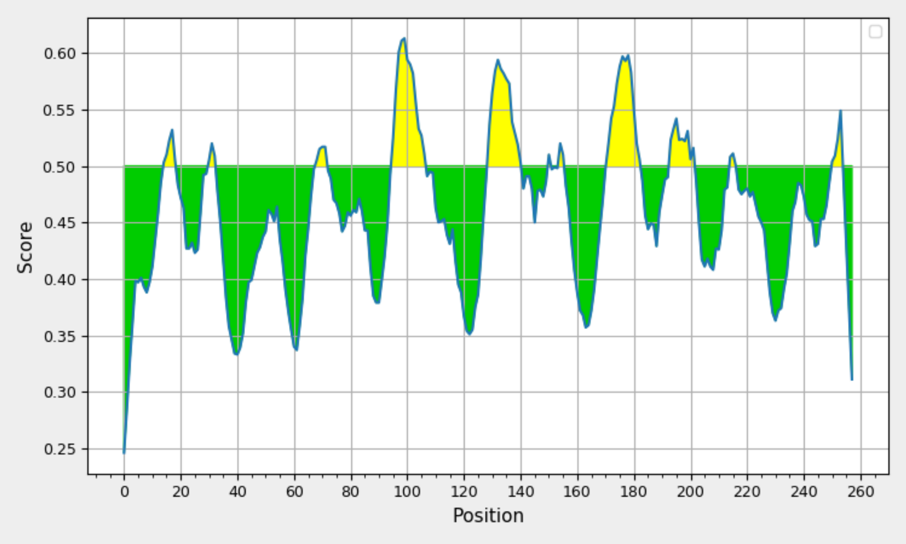

Predicted peptides: XP_010762134 [Pb18]

No.	Start	End	Peptide	Length
1	15	19	**GTAKS**	5
2	31	32	KL	2
3	69	72	**PNGA**	4
4	96	107	**ERRVLLREDDEF**	12
5	130	141	LEEREAGRTIDV	12
6	151	151	**E**	1
7	153	153	V	1
8	155	156	**PA**	2
9	172	183	**GTGKVATTEQAQ**	12
10	194	202	**NEKISPEAA**	9
11	215	216	ES	2
12	239	240	PA	2
13	249	276	MIGISYPANGQITNSSRSCRYHQRPFID	28

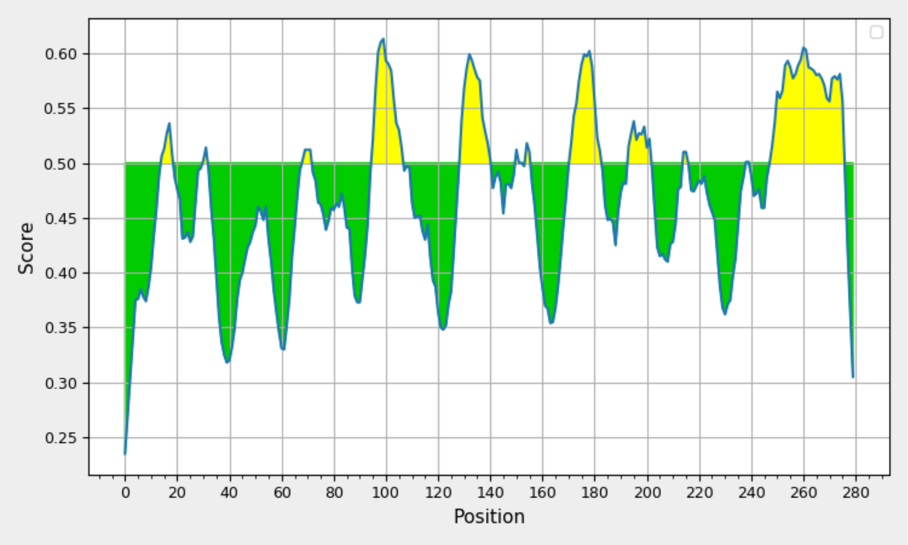

Journal of
Fungi

Protein name: Phenylacetone monooxydase

Predicted peptides: XP_002797384 [Pb01]

No.	Start	End	Peptide	Length
1	5	63	ISNEVSFDPDVLKAKYIEERNKRIRDDG IKQYQAIDTDNFKFSVEDPYTKPGFSRAPID	59
2	110	110	Y	1
3	127	141	EELNYIPTEKYARAP	15
4	174	174	T	1
5	204	213	KLPGVPGIEK	10
6	224	241	WDYDYTGGDTRGGLSKLA	18
7	281	323	RLDQPTDPAWAASLKKGWQQKR MDNFNIIVSGGHQDEDLVHDG	43
8	333	360	VFGGADQPTAKKEAASAMQMA DFKKMEQ	28
9	366	385	DQIVKDPATAGRLKAYYNQF	20
10	411	416	GKGIEC	6
11	424	424	A	1
12	427	427	K	1
13	442	471	FSTDYSKRSGVEIKGLN	30

Bepipred Linear Epitope Prediction 2.0

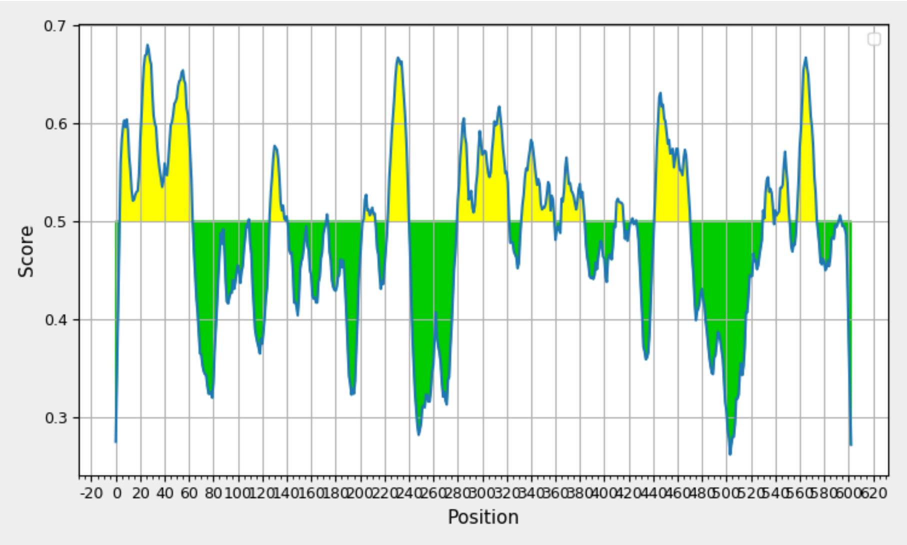

			GTTLTEKWEDGEK	
14	531	539	EAEEQWVKT	9
15	541	552	MEGGKLQQNFVK	12
16	559	575	YNQEGQITERALRNSNY	17
17	593	595	GKL	3

Predicted peptides: XP_010759514 [Pb18]

No.	Start	End	Peptide	Length
1	5	64	TSNEVTFDPDVLKAKYLEERNKRLRDDGI KQYQAIETGNLKYSVEDPYTKPGFSRASIDE	60
2	108	110	NRY	3
3	127	142	EELNYIPTEKYARAPE	16
4	171	174	WDET	4
5	203	208	PKLPGV	6
6	224	241	WDYDYTGGDTTGGLSKLA	18
7	277	323	SVDIRLDQPTDPAWAASLQKGWQQ KRMDNFNIIVSGGHQDEDLVQDG	47
8	334	349	YGGRDQPTAKKEAASA	16
9	351	360	QMADFKKMEQ	10
10	367	385	QIVKDPATAARLKAYYNQF	19

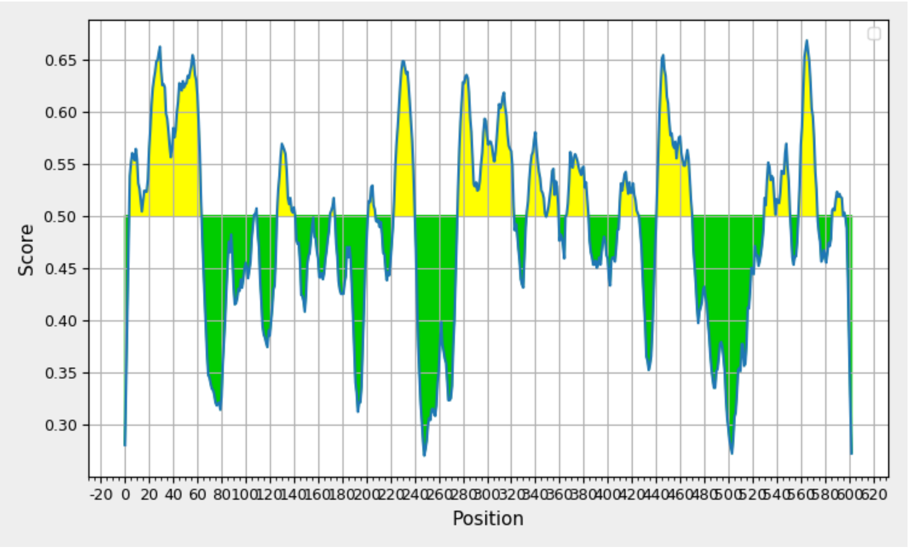

11	411	426	GKGIERITENGIVANG	16
12	442	470	FSTDYSKRSGIEIRGLNGTTLTEKWEDGE	29
13	531	552	EAEQQWVEAIMEGGKLQQNFVK	22
14	559	574	YNQEGQITERALRNSN	16
15	587	595	KDWREAGKL	9
16	597	597	G	1

Journal of Fungi

Protein name: Uncharacterized Protein (PRTases type I Superfamily)

Predicted peptides: XP_015701261 [Pb01]

No.	Start	End	Peptide	Length
1	54	55	DG	2
2	69	103	ENLGRDDDPVEQLGTKVTRTQWLDLSSLEM ANLIG	35
3	128	144	KDVQVAQQQLGREGEKT	17
4	153	170	NKDKPKKGKLPADMLESG	18
5	189	201	AKDIDQHDQLAAE	13

Bepipred Linear Epitope Prediction 2.0

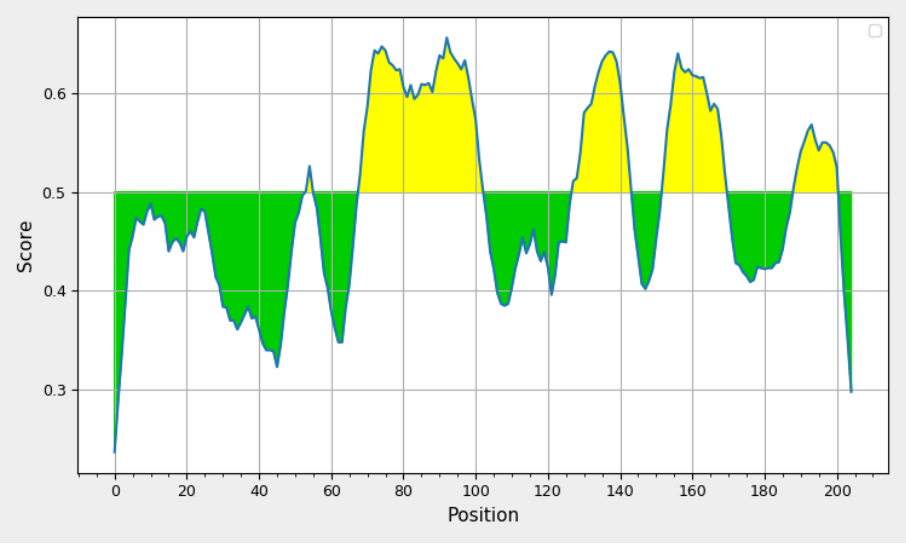

Predicted peptides: XP_010756299 [Pb18]

No.	Start	End	Peptide	Length
1	53	56	PDGA	4
2	69	102	ENLGRDDDPVEQLGTKVTRTQWLDLSSLEM ANLI	34
3	129	143	DVQVAQQQLGREGEK	15
4	153	170	NKDKLKKGKLPADMLESG	18
5	189	201	**AKDIDQHDQLAAE**	13

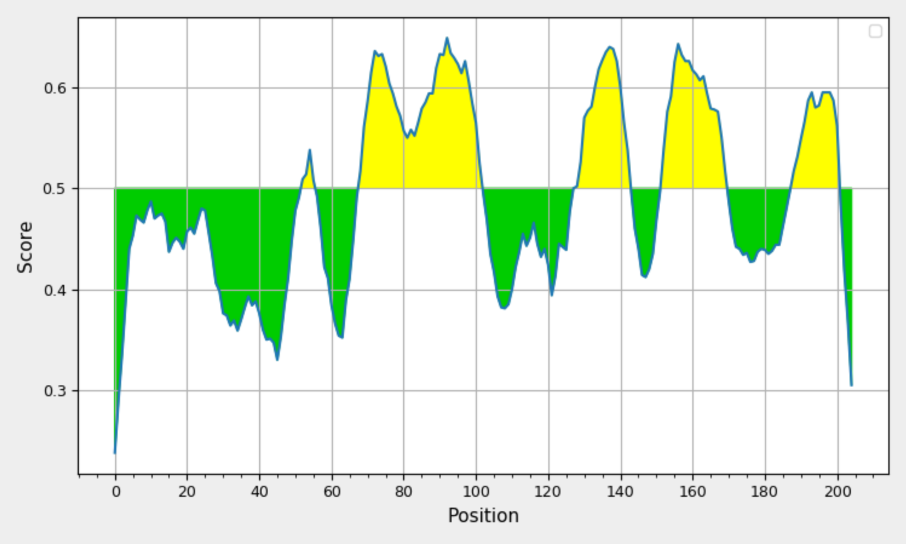

Journal of Fungi

Protein name: Proteasome subunit alpha

Predicted peptides: XP_002797532 [Pb01]

No.	Start	End	Peptide	Length
1	5	7	YSF	3
2	29	30	NQ	2
3	50	62	KSSSPLIEPPSLS	13
4	96	105	AYKRIYNEYP	10
5	122	127	TQSGGV	6
6	142	170	GVKPIPAEGELDTNKKKASGKTGGILKGG	29
7	202	212	EKRYTEGLELE	11
8	227	233	EGEMNGE	7
9	246	272	HLLGYEGVEGARGPRFRKLSKEEIEDY	27

Bepipred Linear Epitope Prediction 2.0

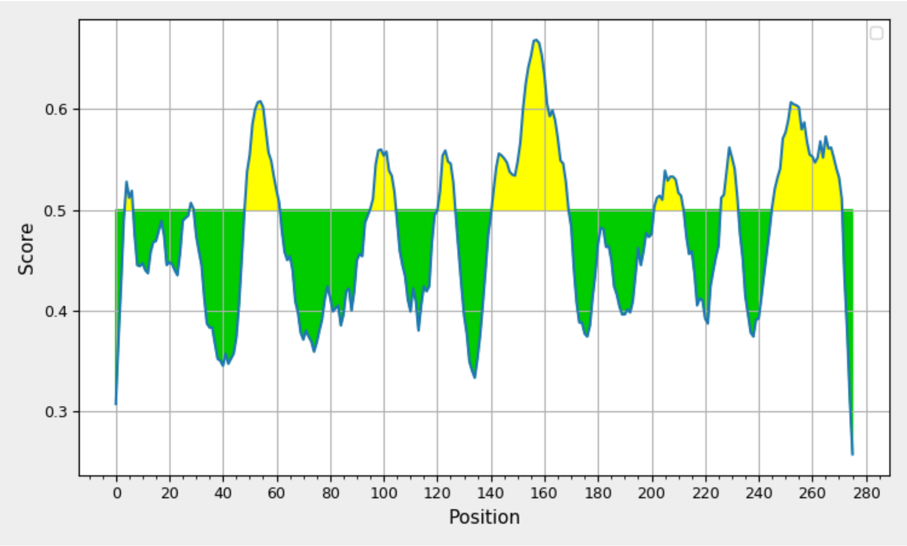

Predicted peptides: XP_010762451 [Pb18]

No.	Start	End	Peptide	Length
1	5	7	**YSF**	3
2	49	59	KSSSPLIDPPS	11
3	96	104	YKRIYNEYP	9
4	120	126	ATQSGGV	7
5	141	169	GVEPTPVEGELDRNKKKASGKTGGILKGG	29
6	181	182	YF	2
7	202	212	KRYTEGLELED	11
8	226	232	**EGEMNGE**	7
9	245	270	HLLGYEGVEGARGPRFRKLSKEEIED	26

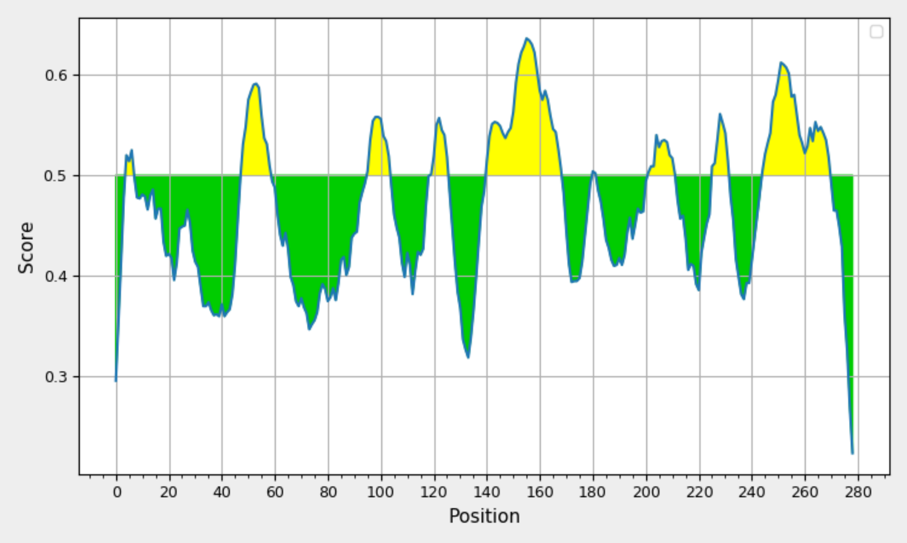

Journal of Fungi

Protein name: Glyceraldehyde-3-Phosphate dehydrogenase

Predicted peptides: Q8X1X3

No.	Start	End	Peptide	Length
1	36	38	**FIE**	3
2	51	60	THGQFKGDIQ	10
3	62	62	S	1
4	80	89	RDPANIPWGK	10
5	105	111	TEKAKAH	7
6	139	142	**SYRP**	4
7	185	202	QKTVDGPSHKDWRGGRTA	18
8	253	256	VTYD	4
9	267	268	**EG**	2
10	281	283	**LVS**	3
11	287	288	**NG**	2

Bepipred Linear Epitope Prediction 2.0

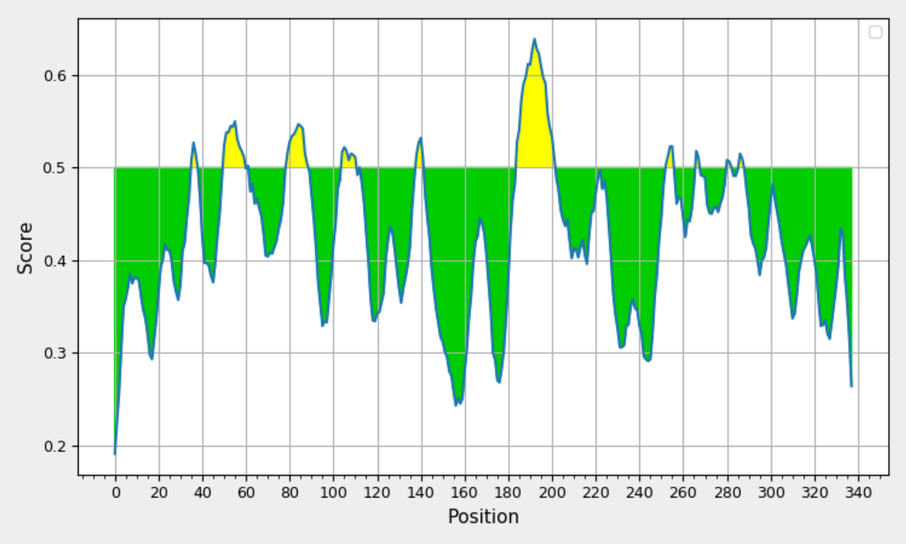

Predicted peptides: XP_010758444 [Pb18]

No.	Start	End	Peptide	Length
1	36	38	**FIE**	3
2	51	58	THGQFKGD	8
3	79	87	ERDPTNIPW	9
4	103	111	TTTEKAKAH	9
5	139	142	**SYRP**	4
6	185	198	QKTVDGPSHKDWRG	14
7	223	223	A	1
8	225	225	N	1
9	254	256	TYD	3
10	267	268	**EG**	2
11	281	283	**LVS**	3
12	287	288	**NG**	2

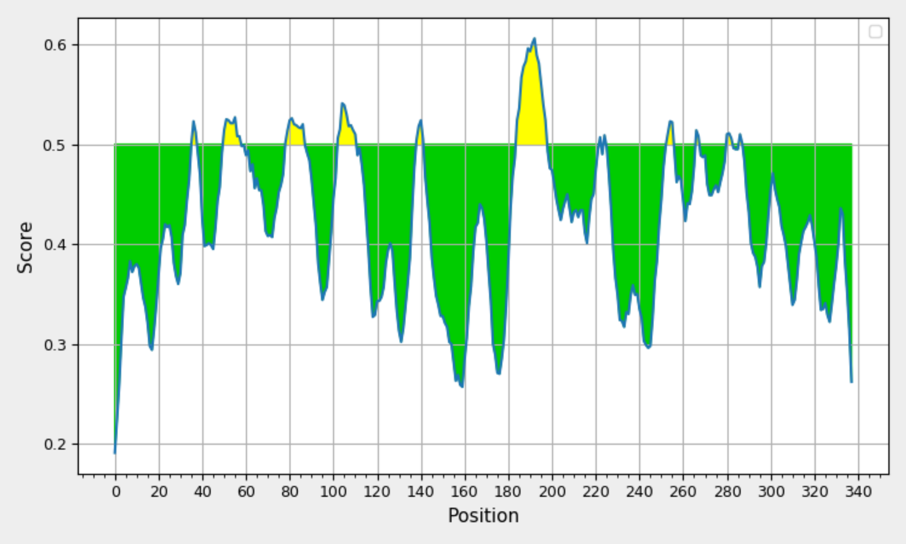

Journal of Fungi

Protein name: Fructose-bisphosphate aldolase 1

Predicted peptides: XP_002796107 [Pb01]

No.	Start	End	Peptide	Length
1	4	11	KDILSRKT	8
2	69	82	FFAGKGVPNGKQEA	14
3	113	129	AKKLLPWLDGMLDADEC	17
4	149	157	**ESVEWNIET**	9
5	180	202	**GGEEDGVNNESVDNNSLYTQPED**	23
6	212	212	**A**	1
7	229	240	VYKPGNVRLHPE	12
8	247	259	AYVKEKTGSSKNK	13
9	272	279	**TKAEFKEA**	8
10	306	333	**LKKKDYLMSAVGNPEGEDKPNKKYFDPR**	28
11	352	357	**DDFNTS**	6

Bepipred Linear Epitope Prediction 2.0

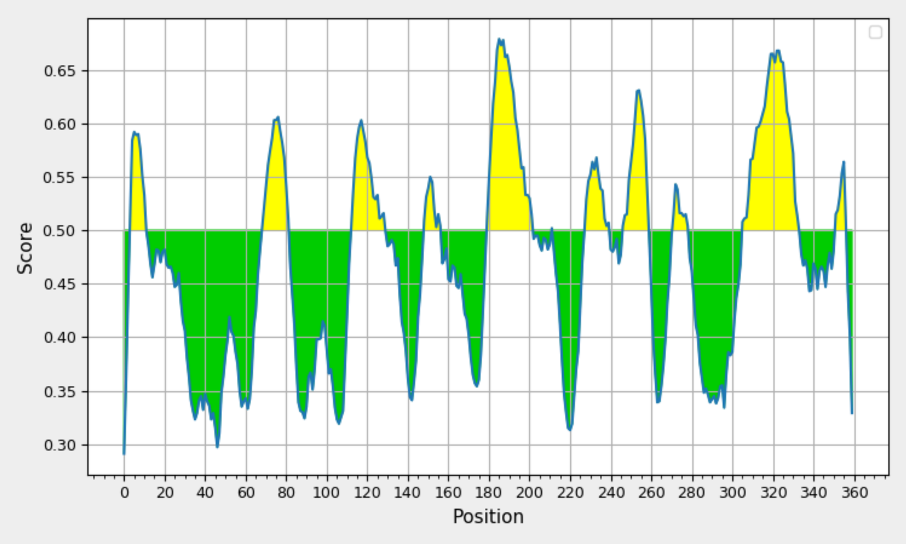

Predicted peptides: XP_010756450 [Pb18]

No.	Start	End	Peptide	Length
1	5	12	DILSRKTG	8
2	70	83	FAGKGVPNGNQEAS	14
3	113	130	AKKLLPWLDGMLDADERY	18
4	133	133	L	1
5	149	157	**ESVEWNIET**	9
6	180	202	**GGEEDGVNNESVDNNSLYTQPED**	23
7	208	208	K	1
8	212	212	**A**	1
9	229	238	VYKPGNVRLH	10
10	248	259	HVKEKTGSSKNK	12
11	272	279	**TKAEFKEA**	8
12	306	333	**LKKKDYLMSAVGNPEGEDKPNKKYFDPR**	28
13	352	357	**DDFNTS**	6

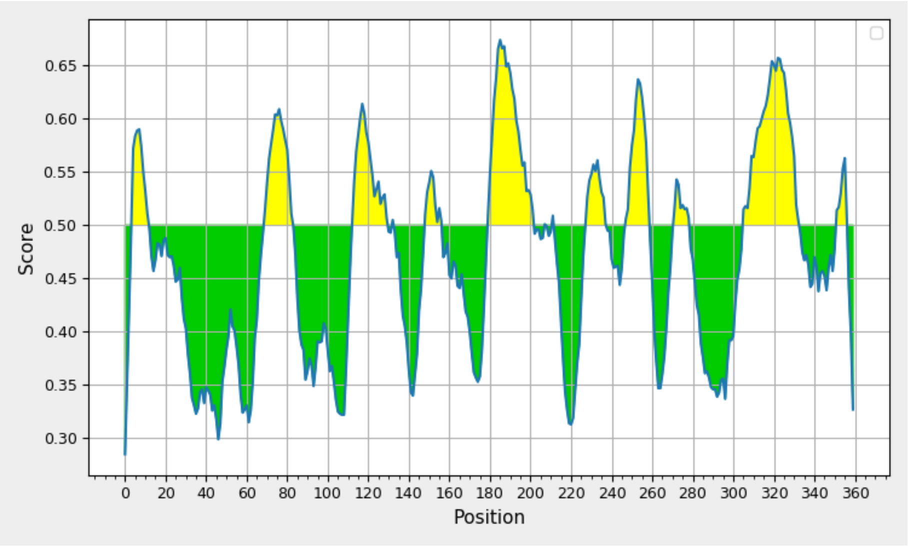

Journal of Fungi

Protein name: Mannitol-1-Phosphate 5-dehydrogenase

Bepipred Linear Epitope Prediction 2.0

Predicted peptides: XP_002791734 [Pb01]

No.	Start	End	Peptide	Length
1	57	60	GEHK	4
2	73	80	**HNLDDVIK**	8
3	129	130	TD	2
4	141	147	DESRRDS	7
5	165	172	TQDPNSGL	8
6	188	211	PFGDVGHPDIKAIHWVDNLEPYIE	24
7	260	277	IVEKHHIPAEEQHDYVQK	18
8	280	289	TRISNPYLED	10
9	299	306	LRKLSRKE	8
10	320	320	Q	1
11	339	356	PDDEESFELHKILKELSA	18
12	364	371	TDLEPDHP	8
13	373	375	YPR	3
14	378	378	**E**	1

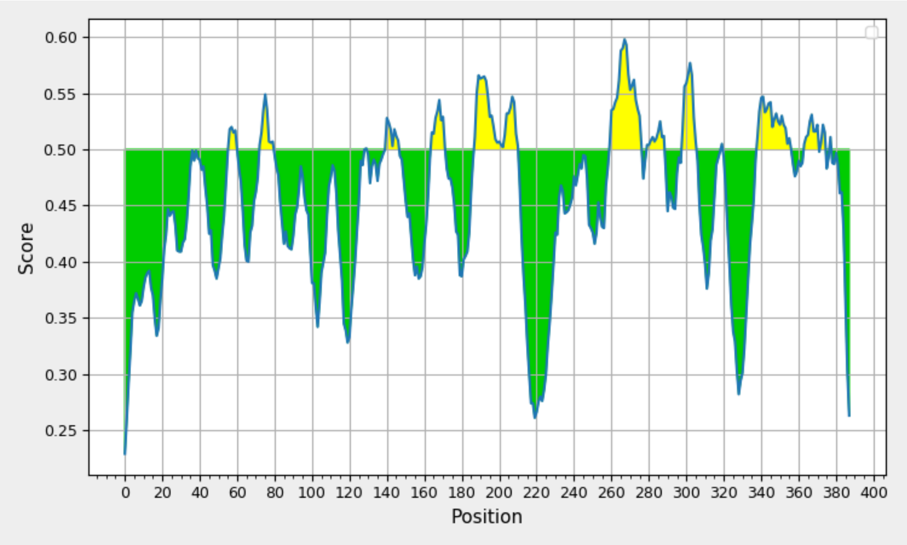

Predicted peptides: XP_010757428 [Pb18]

No.	Start	End	Peptide	Length
1	37	43	SVIDALQ	7
2	56	61	EGEHKK	6
3	73	80	**HNLDDVIK**	8
4	141	143	DES	3
5	145	146	RD	2
6	164	172	PTQDPNSGL	9
7	189	201	FGDVGHPDIKAIH	13
8	204	211	DNLEPYIE	8
9	260	277	IVEKHHIPAAEQHDYVQK	18
10	280	290	TRISNPYLEDV	11
11	296	306	RAPLRKLSRKE	11
12	339	357	PDDEESFELHKILKELSAA	19
13	361	361	T	1
14	363	371	LTDLEADHP	9
15	378	378	E	1
16	382	382	K	1

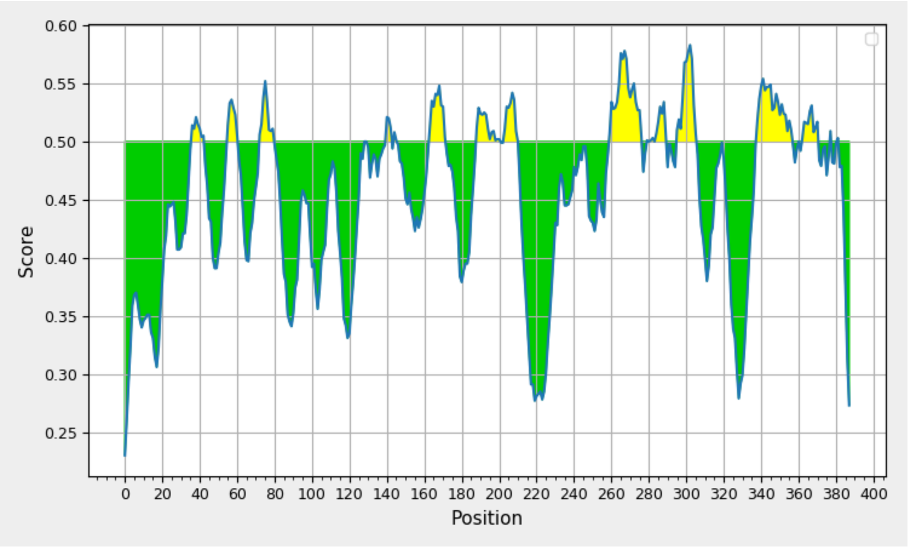

Protein name: Hsp72-like protein

Predicted peptides: XP_002790117 [Pb01]

No.	Start	End	Peptide	Length
1	29	32	NDQG	4
2	58	58	A	1
3	73	94	GRKFADPEVQSDMKHFPFKVID	22
4	131	131	S	1
5	134	134	G	1
6	149	151	NDS	3
7	186	190	KAEGE	5
8	244	257	KRKHKKDLSSNARA	14
9	270	275	RTLSSA	6
10	323	327	KIDKS	5
11	349	362	SDFFNGKEPNKSIN	14
12	380	390	GDTTSKSTNEI	11
13	400	407	VGIETAGG	8
14	413	424	IKRNTTIPTKKS	12
15	442	454	FEGERARTKDNNL	13
16	463	471	IPPAPRGVP	9

Bepipred Linear Epitope Prediction 2.0

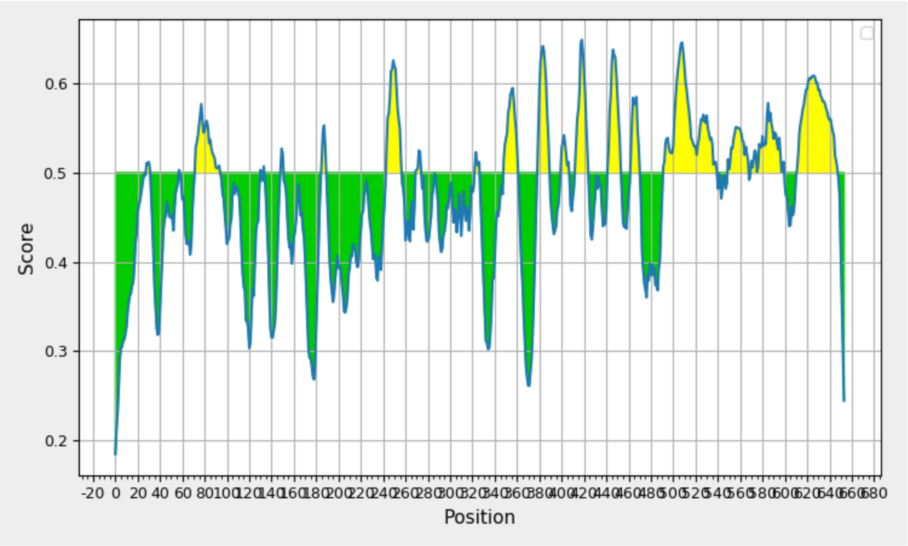

17	492	540	KGTGKTNKIVITNDKGRLSKEEIERMLAE AEKYKAEDEAEASRISAKNG	49
18	550	568	NTISDSKVDEKLDASDKEK	19
19	571	600	TEIDKTVSWLDENQTATKEEFEAQQKELES	30
20	612	648	AGGEGGAPGAGFPGAGGPGGFPGA GAGGAHSGGDDGP	37

Predicted peptides: XP_010763478 [Pb18]

No.	Start	End	Peptide	Length
1	27	27	I	1
2	29	32	**NDQG**	4
3	58	59	AM	2
4	74	96	RKFADPEVQSDMKHFPFKVIDKA	23
5	134	134	**G**	1
6	149	151	**NDS**	3
7	186	190	**KAEGE**	5
8	244	257	**KRKHKKDLSSNARA**	14
9	270	274	RTLSS	5
10	324	327	IDKS	4
11	349	361	SDFFNGKEPNKSI	13

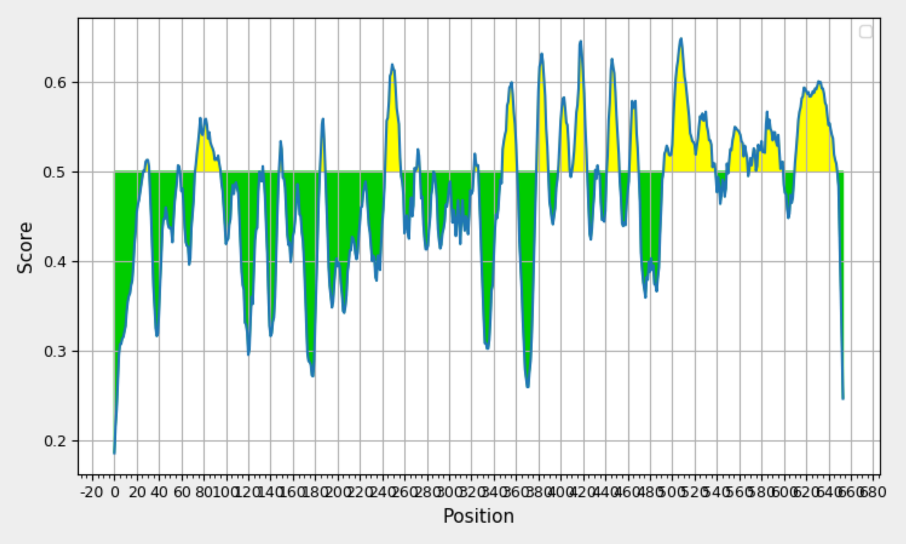

12	380	389	GDTTSKSTNE	10
13	399	409	SVGIETAGGVM	11
14	411	424	PLIKRNTTIPTKKS	14
15	432	432	D	1
16	434	434	Q	1
17	443	453	EGERARTKDNN	11
18	463	470	IPPAPRGV	8
19	494	539	TGKTNKIVITNDKGRLSKEEIERML AEAEKYKAEDEAEASRISAKN	46
20	550	550	N	1
21	552	568	ISDSKVDEKLDASDKEK	17
22	570	600	KTEIDKTVSWLDENQTATKEEFEAQQKELES	31
23	611	648	GAGGEGGAPGAGFPGAGGPG GFPGAGASGAHSGGDDGP	38

Journal of Fungi

Protein name: Citrate synthase

Predicted peptides: XP_002793640 [Pb01]

No.	Start	End	Peptide	Length
1	6	10	RATRQ	5
2	24	38	SSRFYSTAESDLKTV	15
3	40	59	EKAIPAKKELFQEVKKRGED	20
4	73	73	M	1
5	107	113	**PKGTSGT**	7
6	129	137	**VPTTPQVRA**	9
7	140	140	S	1
8	143	150	AQKSHLPK	8
9	160	161	**PK**	2
10	181	199	KFAKAYADGLSKASYWEPT	19
11	222	234	**EIEKVGTQELDPT**	13
12	249	253	SKANE	5
13	304	307	LHGL	4
14	322	331	TIGNKFTDAD	10
15	344	359	QVVPGYGHGVLRKPDP	16

Bepipred Linear Epitope Prediction 2.0

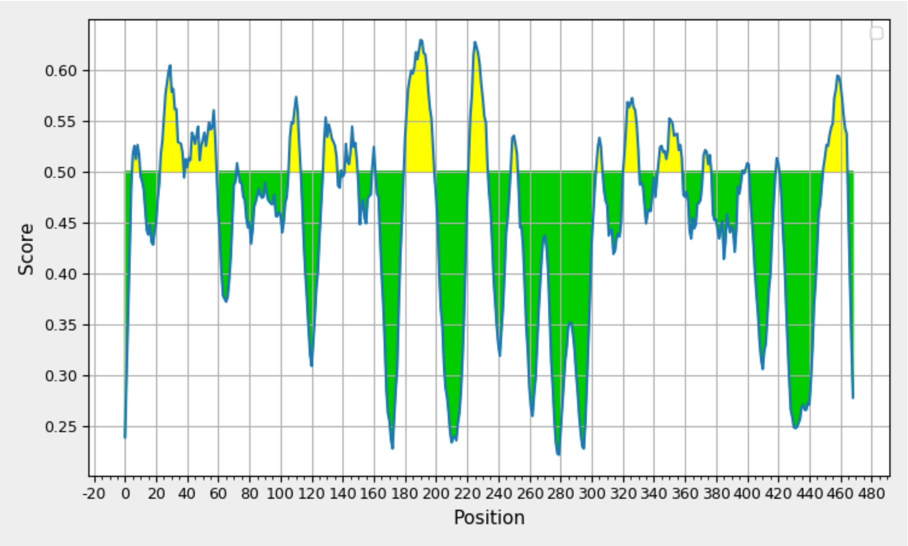

16	373	377	**EIQAN**	5
17	398	398	**G**	1
18	400	402	TKN	3
19	420	421	FR	2
20	450	**465**	**LPIERPKSVNLQGLLD**	16

Predicted peptides: XP_010760342 [Pb18]

No.	Start	End	Peptide	Length
1	6	59	RATRQALKASQSAAWSFSSSRFYSTAESDL KTVFEKAIPAKKELFQEVKKRGED	54
2	73	74	MR	2
3	107	113	**PKGTSGT**	7
4	129	137	**VPTTPQVRA**	9
5	143	150	AQKSHLPR	8
6	160	161	**PK**	2
7	181	200	KFAKAYADGLSKASYWEPTF	20
8	222	234	**EIEKVGTQELDPT**	13
9	249	253	SKVNE	5
10	304	308	LHGLA	5
11	321	331	STIGNKFTDAD	11

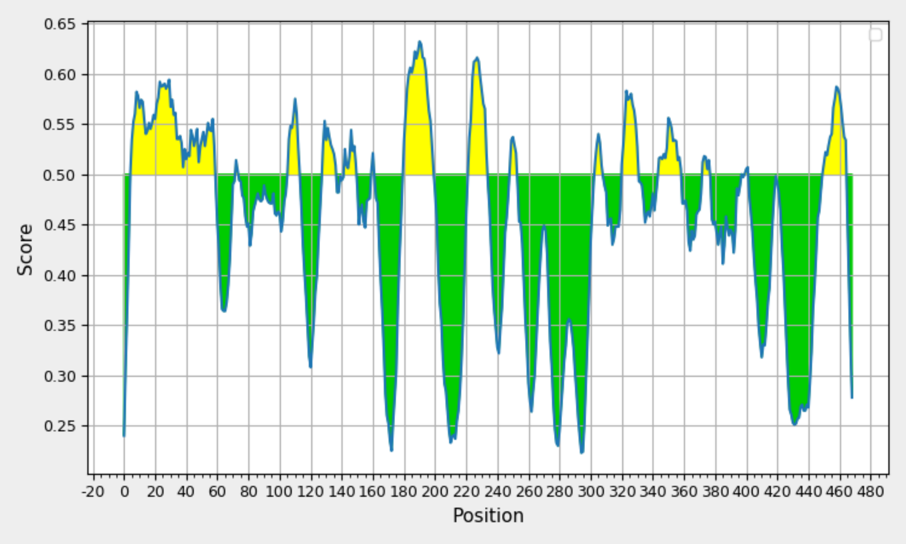

12	345	359	VVPGYGHGVLRKPDP	15
13	373	377	**EIQAN**	5
14	398	398	**G**	1
15	401	402	KN	2
16	450	465	**LPIERPKSVNLQGLLD**	16

Journal of Fungi

Protein name: Isocitrate lyase

Predicted peptides: XP_002791040 [Pb01]

No.	Start	End	Peptide	Length
1	5	62	EQEDQKYWAEVQAVKEWWQ DSRWRYTKRPFTAEQIVAK RGNLKIEYPSNVQSKKLWGI	58
2	64	69	EERFKN	6
3	109	122	DEPSPDLADYPMNT	14
4	138	160	DRKQREERLRAPKSQRASLPNID	23
5	201	216	PGTKKCGHMAGKVLVP	16
6	251	255	LITST	5
7	273	273	K	1
8	277	277	D	1
9	283	290	EQAGKNGA	8
10	297	305	DKWNAEAGL	9
11	319	323	SSIPN	5
12	340	370	NTEARAIAKKITGSDIYFDWDAARTREGFYR	31
13	398	403	LPDYAQ	6

Bepipred Linear Epitope Prediction 2.0

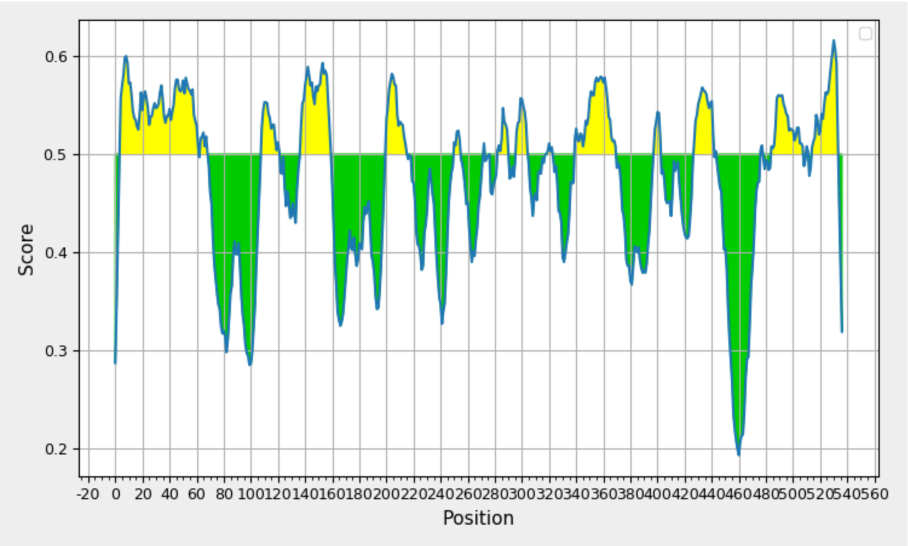

14	428	442	FNWKAAMPRGEQETY	15
15	444	444	R	1
16	477	478	MN	2
17	485	508	ELVQEPEMENGVDVVTHQKWSGAN	24
18	511	512	DE	2
19	515	534	KMVSGGISSTSAMGKGVTED	20

Predicted peptides: XP_010757468 [Pb18]

No.	Start	End	Peptide	Length
1	5	62	EQEDQKYWAEVQAVKEWWQD SRWRYTKRPFTAEQIVAKR GNLKIEYPSNVQSKKLWGI	58
2	64	69	EERFKN	6
3	109	121	DEPSPDLADYPMN	13
4	137	160	HDRKQREERLRAPKSQRASLPNID	24
5	200	216	APGTKKCGHMAGKVLVP	17
6	218	219	SE	2
7	251	255	LITST	5
8	270	273	PNIK	4
9	286	290	GKNGA	5

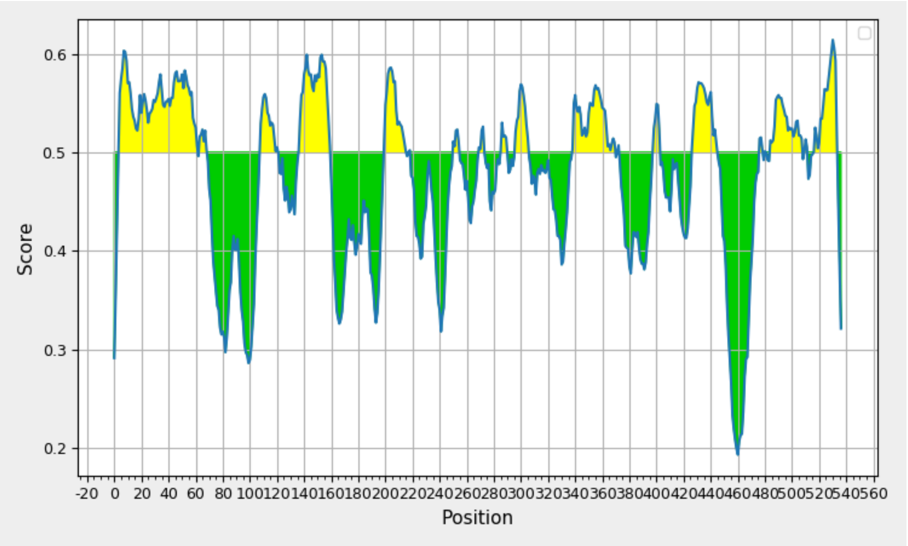

10	297	306	DKWNAEAGLK	10
11	339	370	SNTEARAIAKKITASDTYFDW DAARTREGFYR	32
12	373	373	G	1
13	399	403	PDYAQ	5
14	427	445	SFNWKAAMPRGEQETYIRR	19
15	477	479	TNG	3
16	481	481	**R**	1
17	485	508	ELVQEPEMENGIDVVTHQKWSGAN	24
18	510	512	VDE	3
19	517	534	VSGGISSTSAMGKGVTED	18

Journal of Fungi

Protein name: Transketolase

Predicted peptides: XP_002793534 [Pb01]

No.	Start	End	Peptide	Length
1	5	7	**NDV**	3
2	52	59	NPKNPQWI	8
3	85	110	AVSLDDLKDFRRLDSITPGHPEAHDT	26
4	138	149	**GAVFNKPGFDLI**	12
5	193	210	IDGDTKVAFTEDVMKRFE	18
6	221	226	DGNNDL	6
7	255	358	GSELQGTGGVHGNPLKADDSKGVKK RFGFDPEQSFVVPQQVYDLYHKK AAEGAAKEQEWNALLQQYASKYPAEHA DFTRRLSGKLPEGWEKNLPRYSPTDPAIA	104
8	387	408	SNNTRWKDAVDFQPPSTGLGDW	22
9	530	542	**NLPQLEASSIEKA**	13
10	594	602	**VFDVQDKEY**	9
11	623	626	GWDR	4
12	640	649	SGPYKEVYKK	10
13	651	653	**EFT**	3

Bepipred Linear Epitope Prediction 2.0

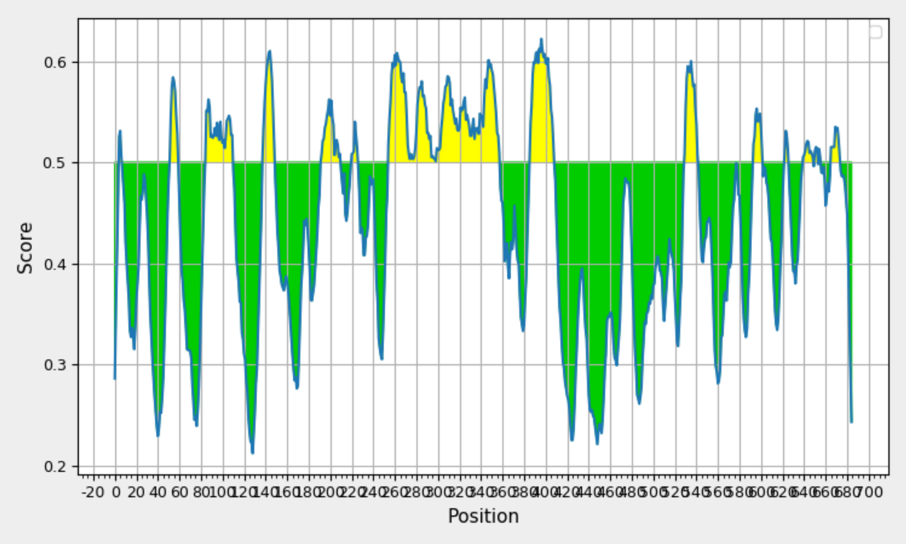

14	655	656	EG	2
15	666	674	**EFYKGSPVL**	9

Predicted peptides: XP_010760067 [Pb18]

No.	Start	End	Peptide	Length
1	5	10	**NDVDQL**	6
2	12	50	INTIRVLAVSIFYLSTIITQSACPISQSVLF LKSRKITA	39
3	95	102	NPKNPQWI	8
4	128	153	AVSLDDLKDFRKLDSITPGHPEAHDT	26
5	181	192	**GAVFNKPGFDLI**	12
6	235	253	SIDGDTKVAFTEDVMKRFE	19
7	262	270	VEDGNNDLA	9
8	298	315	GSELQGTGGVHGNPLKAD	18
9	324	400	FGFDPEQSFVVPQQVYDLYH KKAAEGAAKEQEWNALLQQYASKYPAEHA DFTRRLSGKLPEGWEKSLPRYSPTDPAI	77
10	430	452	SNNTRWKDAVDFQPPSTGLGDWT	23
11	573	585	**NLPQLEASSIEKA**	13
12	637	645	**VFDVQDKEY**	9
13	647	649	LSV	3

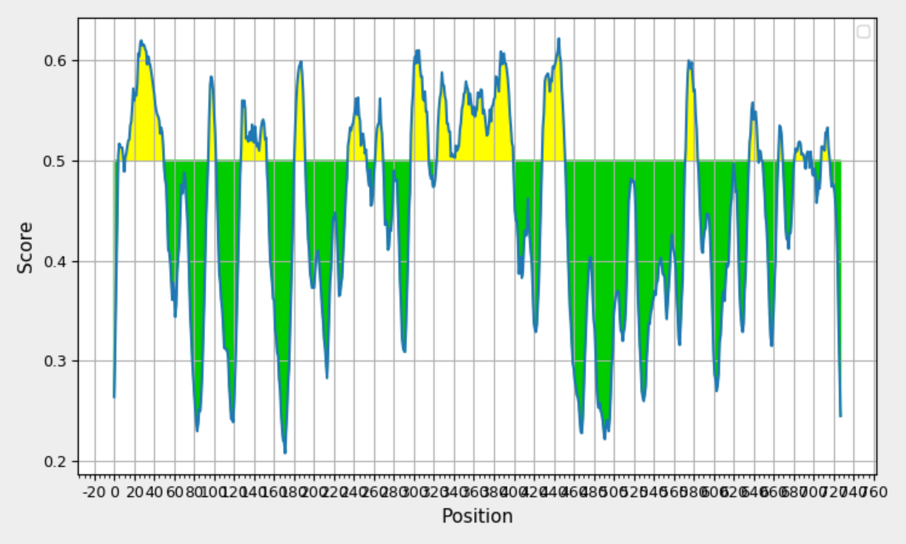

14	666	670	GWDRY	5
15	682	692	ASGPYKEVYKK	11
16	694	696	**EFT**	3
17	698	698	E	1
18	709	717	**EFYKGSPVL**	9

Protein name: Enolase

Bepipred Linear Epitope Prediction 2.0

Predicted peptides: XP_015703472 [Pb01]

No.	Start	End	Peptide	Length
1	13	16	**DSRG**	4
2	40	60	STGQHEACELRDGDQSKWLGK	21
3	82	89	**IDVKDQSK**	8
4	97	106	LDGTPNKSKL	10
5	138	143	AGTKKP	6
6	158	163	SHAGGR	6
7	195	232	RLRSLSQTEGPRQEEVRQSAGNVGDEG GVAPDIQTPEE	38
8	247	247	T	1
9	264	292	ADEKKYDLDFKNPDSDKSKWLTYEQLADL	29
10	310	318	AEDDWEAWS	9
11	338	346	PIRIKKAIE	9
12	387	391	**GETED**	5
13	435	443	**VYAGDKFRA**	9

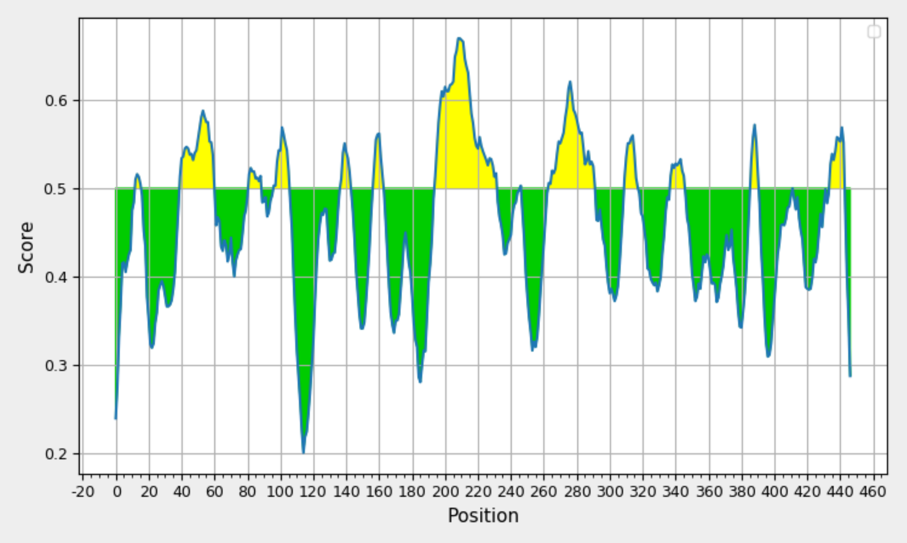

Predicted peptides: XP_010759701 [Pb18]

No.	Start	End	Peptide	Length
1	13	16	DSRG	4
2	40	60	STGQHEACELRDGDKSKWLGK	21
3	82	89	IDVKDQSK	8
4	96	106	KLDGTPNKSKL	11
5	137	144	LAGTKKPY	8
6	157	164	GSHAGGRL	8
7	198	210	KKKYGQSAGNVGD	13
8	218	223	IQTPEE	6
9	237	238	YT	2
10	256	283	DEKKYDLDFKNPDSDKSKWLTYEQLADL	28
11	301	310	AEDDWEAWSY	10
12	330	337	IRIKKAIE	8
13	378	382	GETED	5
14	423	423	S	1
15	426	434	VYAGDKFRA	9

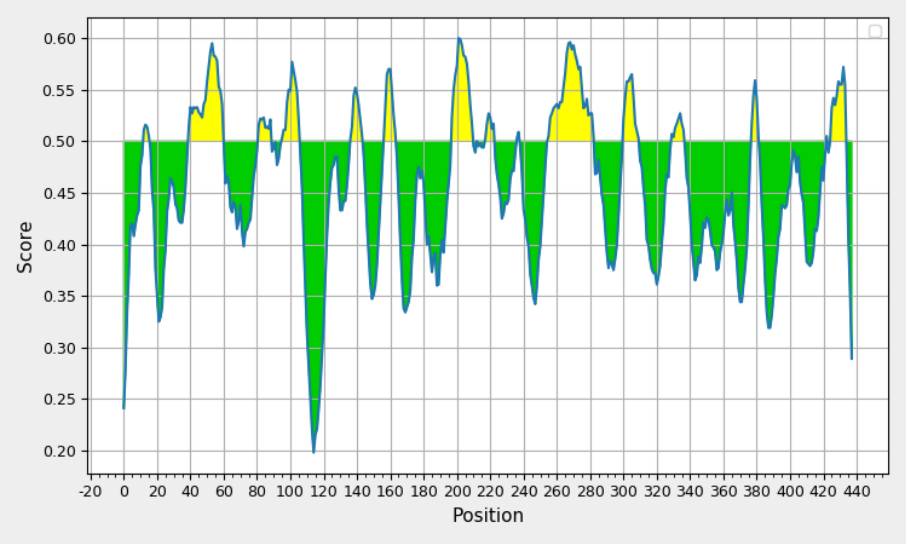

Journal of Fungi

Protein name: Heat shock protein 60, mitochondrial

Predicted peptides: XP_002789992 [Pb01]

No.	Start	End	Peptide	Length
1	5	49	FTSSRASVLSSASSTRAPLSRFRSAG VGLQQQRFAHKELKFGVEA	45
2	69	70	GP	2
3	84	84	P	1
4	99	103	**LQDKF**	5
5	120	123	**EVAG**	4
6	166	166	E	1
7	168	195	LQTNKRDITTTEEIAQVATISANGDTHV	28
8	204	209	**EKVGKE**	6
9	219	221	TID	3
10	231	232	RF	2
11	234	252	RGYVSPYFITDTKAQKVEF	19
12	258	284	LLSEKKISAVQDIIPALEASTSLRRPL	27
13	290	290	D	1
14	292	295	EGEA	4

Bepipred Linear Epitope Prediction 2.0

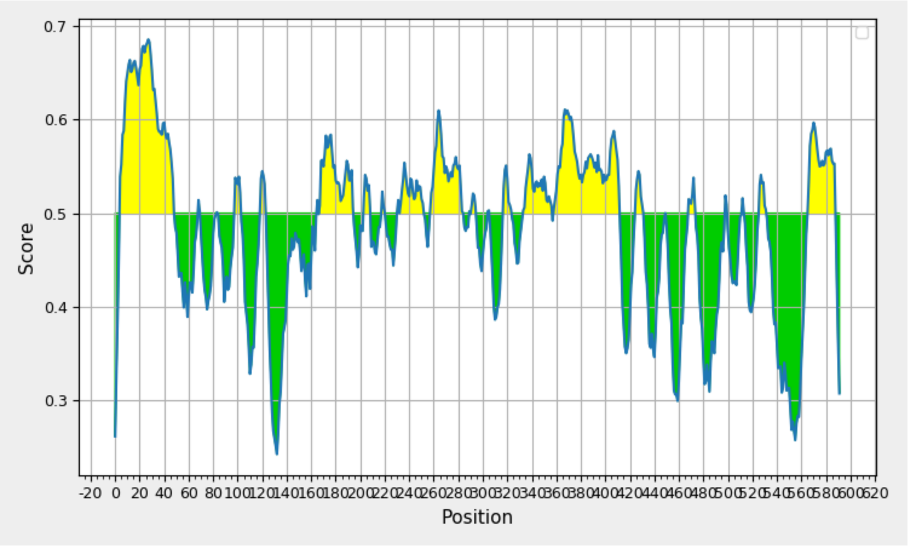

15	305	306	RG	2
16	318	324	FGDNRKS	7
17	334	357	NATVFTDELDLKLEKATPDMLGST	24
18	359	412	SITITKEDTIILNGEGSKDAIAQRCEQ IRSVISDPATSDYEKEKLQERLAKLSG	54
19	426	432	EVGEKKD	7
20	450	450	**L**	1
21	469	474	**NPTNFD**	6
22	499	500	LE	2
23	513	514	AG	2
24	527	532	YVDMIG	6
25	567	588	**PEEKVPAGSGAGGMGGGMGGMG**	22

Predicted peptides: XP_010763632 [Pb18]

No.	Start	End	Peptide	Length
1	5	11	FTSSRAL	7
2	17	51	SSASSTRAPLSRFRSAGVGLQQQRF AHKELKFGVE	35
3	72	72	G	1
4	86	88	SPK	3
5	102	106	**LQDKF**	5
6	123	126	**EVAG**	4
7	172	187	QANKRDITTTEEIAQV	16
8	191	198	SANGDTHV	8
9	204	204	N	1
10	207	212	**EKVGKE**	6
11	221	224	KTID	4
12	234	255	RFDRGYVSPYFITDTKAQKVEF	22
13	261	286	LLSEKKISAVQDIIPALEASTSLRRP	26
14	296	298	GEA	3
15	321	328	FGDNRKSI	8
16	341	415	FTDELDLKLEKATPDMLGSTGSITITK EDTIILNGEGSKDAIAQRCEQIRSVIS	75

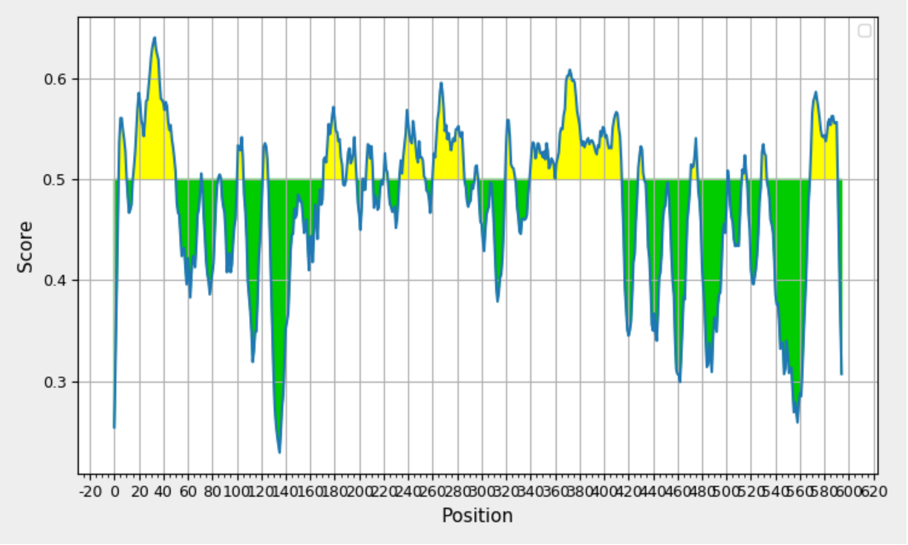

			DPATSDYEKEKLQERLAKLSG	
17	429	433	EVGEK	5
18	472	477	**NPTNFD**	6
19	502	502	**L**	1
20	514	517	DFAS	4
21	530	533	YVDM	4
22	570	591	**PEEKVPAGSGAGGMGGGMGGMG**	22

Protein name: Uncharacterized protein

Predicted peptides: XP_002797075 [Pb01]

No.	Start	End	Peptide	Length
1	5	11	ATRQVVN	7
2	22	27	SPRTLV	6
3	41	42	**EV**	2
4	45	61	FLNLKNTPSPRARNTGR	17
5	72	72	A	1
6	74	78	NAAVE	5
7	86	148	IPSLSKLLPESSMFNSSKESYPT KSALAKPRIKARTKEARRNRERDK KREKKLADYGDGNSPE	63
8	158	187	SHHDHNVAVMQNPYYNAFHVAT NSLEVQDL	30
9	192	242	TICSEGVGYSEAPTKSPGDLVLKPPPW LQHMDCFPKKKRMFPFRHQANVLR	51
10	260	317	REVMEKLKVKKAMTSNEEANEMAQEQEQ EQEQEQEQEQEQGQRREQEQEQEREMEGIR	58

Bepipred Linear Epitope Prediction 2.0

12

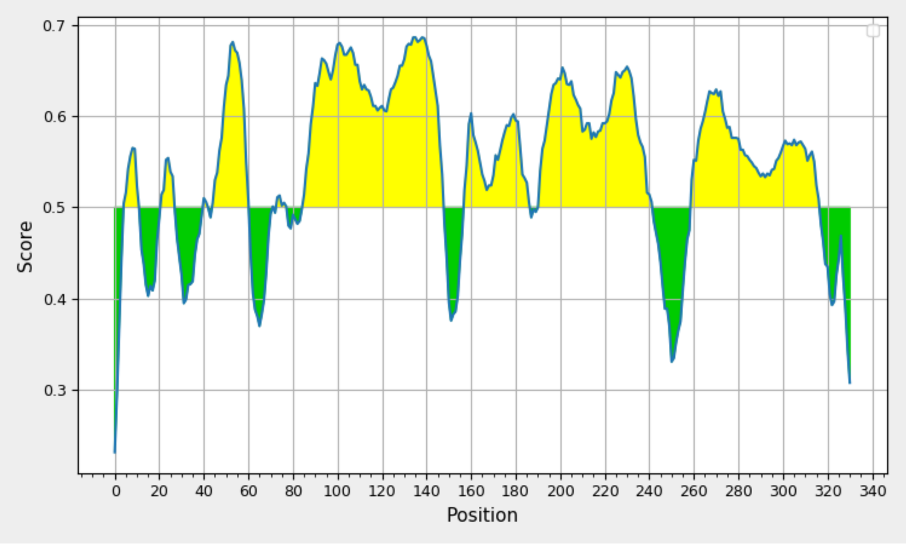

Predicted peptides: XP_010759641 [Pb18]

No.	Start	End	Peptide	Length
1	5	11	AARQVAN	7
2	22	28	SPRTLVE	7
3	41	42	EV	2
4	46	61	LNLKNTPSPRAMNTGR	16
5	71	71	D	1
6	85	150	PIPSLSKLLPESSMLNSGKESYP TKSTLAKPRIKARTREARRNRERAKKK RQNLADYDGDGSSPAP	66
7	159	244	SHHDHNVAVMQNPYYNAF HIATNLLEVQDLMKTATNCS EGVGYSEEPTKSLGDLVLKPPPWLQH MDCFPKKKRMFPFRHQGNVLRI	86
8	262	308	EVMEKLKVKKAMTSNKKANEMA QEKEQGQGQRIEQEQEQERESRELE	47

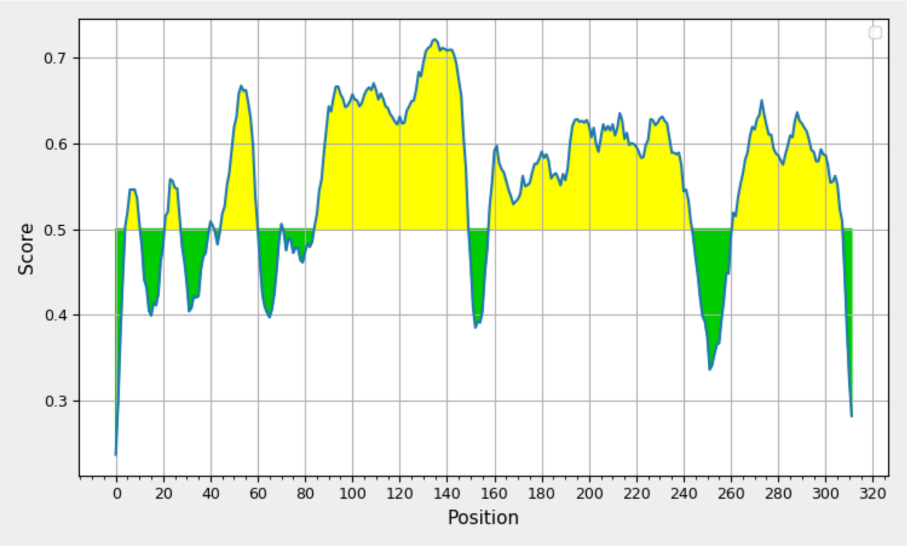

Journal of Fungi

Protein name: Nucleoside diphosphate kinase

Predicted peptides: XP_002794019 [Pb01]

No.	Start	End	Peptide	Length
1	15	21	GVQRGLV	7
2	38	63	IKLVTPSKEHLEKHYEDLSSKPFFNG	26
3	95	111	NPADSAPGTIRGDYAID	17
4	123	129	VESAKKE	7
5	131	131	A	1
6	133	149	WFKPEELVKYEQSQANW	17

Bepipred Linear Epitope Prediction 2.0

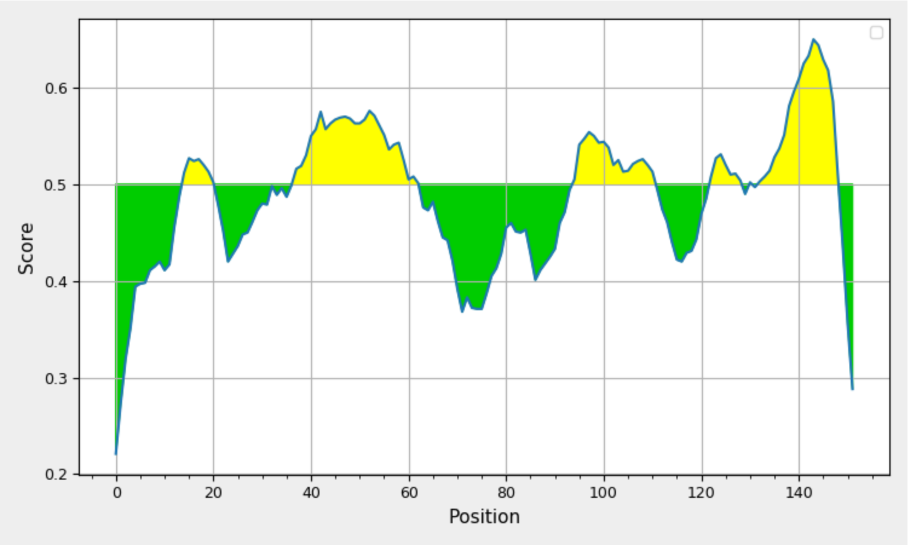

Predicted peptides: XP_010762888 [Pb18]

No.	Start	End	Peptide	Length
1	15	21	GVQRGLV	7
2	38	63	IKLVTPSKEHLEKHYEDLSSKPFFNG	26
3	94	111	TNPADSAPGTIRGDYAID	18
4	123	129	VESAKKE	7
5	131	131	A	1
6	133	149	WFKPEELVKYEQSQANW	17

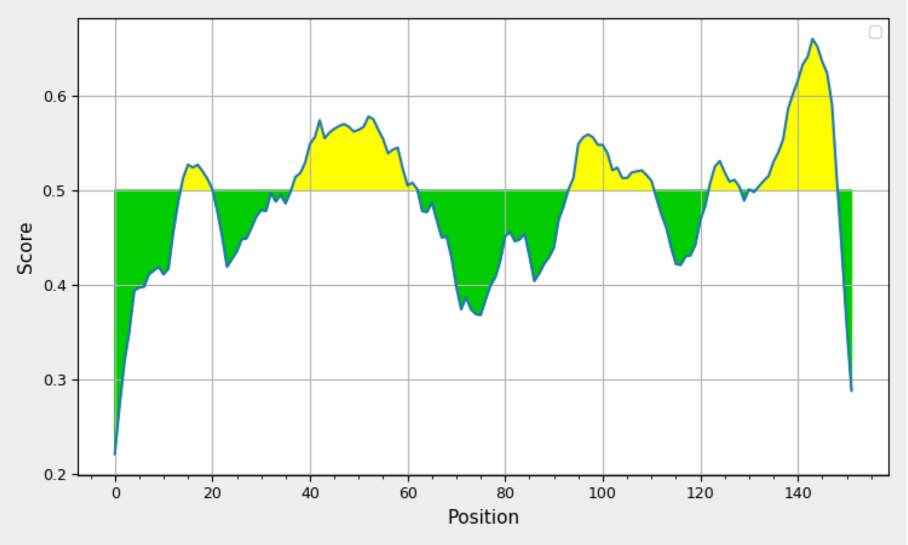

Journal of Fungi

Protein name: Uncharacterized protein (Thioredoxin-like superfamily)

Predicted peptides: XP_002794671 [Pb01]

No.	Start	End	Peptide	Length
1	4	17	EQRAPLRLGSTAPN	14
2	20	20	**A**	1
3	27	34	IDFHEFIG	8
4	81	97	**KSHYDWINDINEVTGSD**	17
5	115	130	DMIDYQDTTNVDEKGM	16
6	151	161	SYPASTGRNAA	11
7	172	200	**TTDKNGVNTPINWNVGDDVIIPPAVKTED**	29
8	203	203	K	1
9	205	206	FG	2

Bepipred Linear Epitope Prediction 2.0

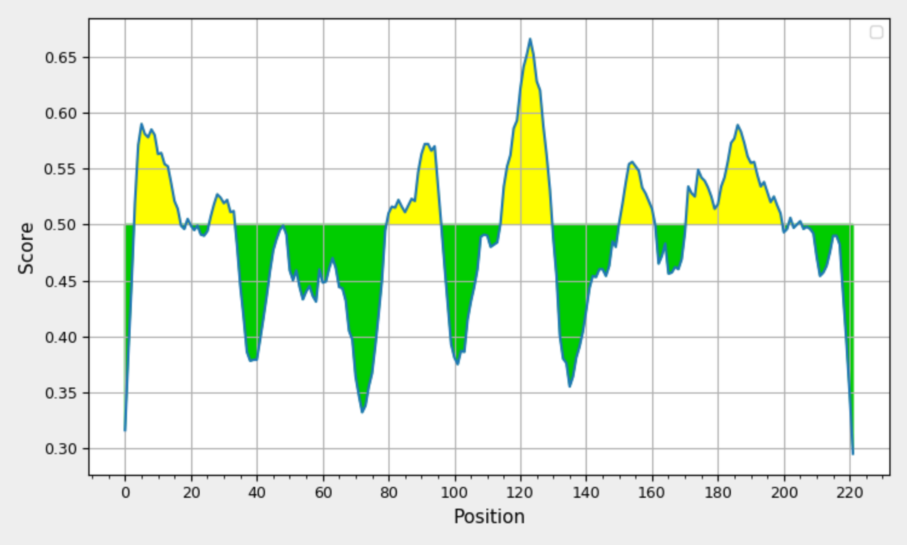

Predicted peptides: XP_010758730 [Pb18]

]	Start	End	Peptide	Length
1	4	17	EHRAPLRLGSTAPN	14
2	20	20	**A**	1
3	27	34	IDFYEFIG	8
4	49	49	P	1
5	81	97	**KSHYDWINDINEVTGSD**	17
6	116	129	MIDYQDTTNVDEKG	14
7	152	161	YPASTGRNAA	10
8	172	200	**TTDKNGVNTPINWNVGDDVIIPPAVKTED**	29
9	202	203	IK	2
10	205	209	FGDVR	5

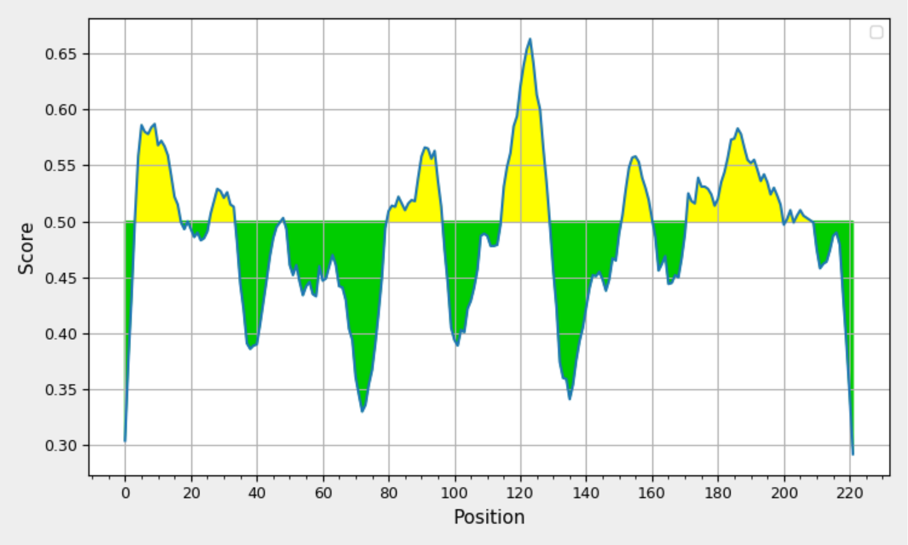

Journal of Fungi

Protein name: Deoxyuridine 5'-triphosphate nucleotidohydrolase

Predicted peptides: XP_002792326 [Pb01]

No.	Start	End	Peptide	Length
1	5	78	TTPRPQTKASEESTTTNITANHP PPSLPSSPLPKRAKITDMARATESSTA PAAASSETATSTNPAVTSLSQLTP	74
2	92	99	**APTRGSAF**	8
3	197	213	VLEVEELEESVRGVGGF	17

Bepipred Linear Epitope Prediction 2.0

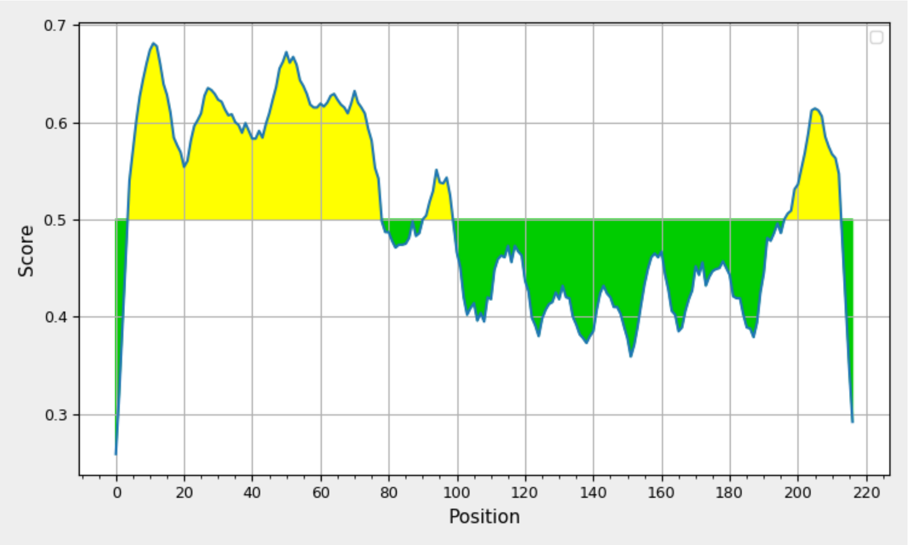

Predicted peptides: XP_010763035 [Pb18]

No.	Start	End	Peptide	Length
1	5	75	TTPRPQTKASAESTTVNHPP PSLPSSPLPKRAKITDMAPATESFTAPA AAYSETATSTNPAVTSLSQLTPP	71
2	88	95	APTRGSAF	8
3	194	209	LEVEELEESVRGVGGF	16

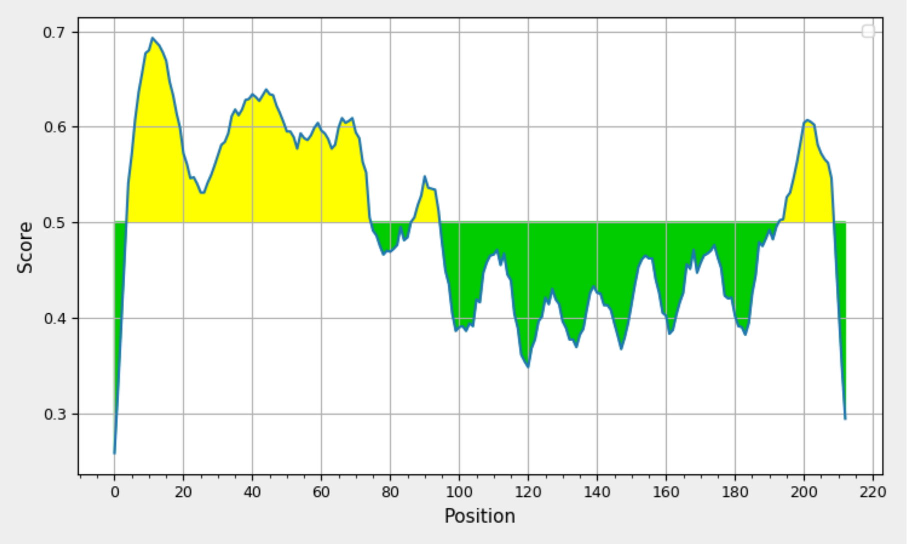

Journal of Fungi

Protein name: Proliferating cell nuclear antigen

Predicted peptides: XP_015700963 [Pb01]

No.	Start	End	Peptide	Length
1	5	13	LPQTLYSIP	9
2	19	50	IPTDTLNLSFTHISTTPGRTGAQYLHSTISPA	32
3	58	64	FNIYPPQ	7
4	151	152	QN	2
5	187	197	MDIDQEHLAIP	11
6	221	221	N	1
7	251	262	RQHTNVDKPDQN	12

Bepipred Linear Epitope Prediction 2.0

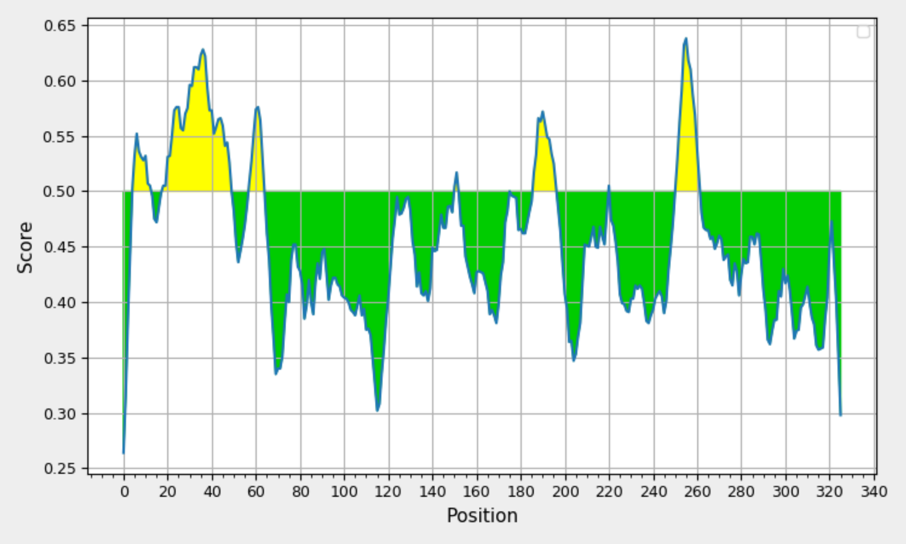

Predicted peptides: XP_010759909 [Pb18]

No.	Start	End	Peptide	Length
1	5	12	SRFSTDSA	8
2	15	125	TGEGEGQEDWALQEDLSPSQPPLDSSTANF<br>APRGPPTFQDRIRNRLPVPRQLKPLAKHQ<br>TLARISDFCKVWGRFFRFHDVDF<br>PLLTGYTFRIDKAVVSTTIGKSENERFLE	111
3	139	168	EQCAPSYSAITGLLSNTRNNEDQSSTRAAN	30
4	193	200	SRQGSDWN	8
5	247	250	SFDS	4
6	264	290	LVSRGYRLSTPMPPITRLEEQTQTRRC	27
7	323	354	NLEKYYDIYDFSPEELEETNALLA<br>DSAMEDGT	32
8	383	391	EGGEADIAN	9
9	397	397	E	1
10	400	404	QRLVA	5
11	417	449	LNEQDHNMIPPNPQAKLAPNKDHH<br>RAQLRRLNS	33
12	468	484	SDASLERATDDTDFGTT	17
13	505	505	A	1
14	511	584	FATLENSGRLSRPTSSLKSPASPTFS	74

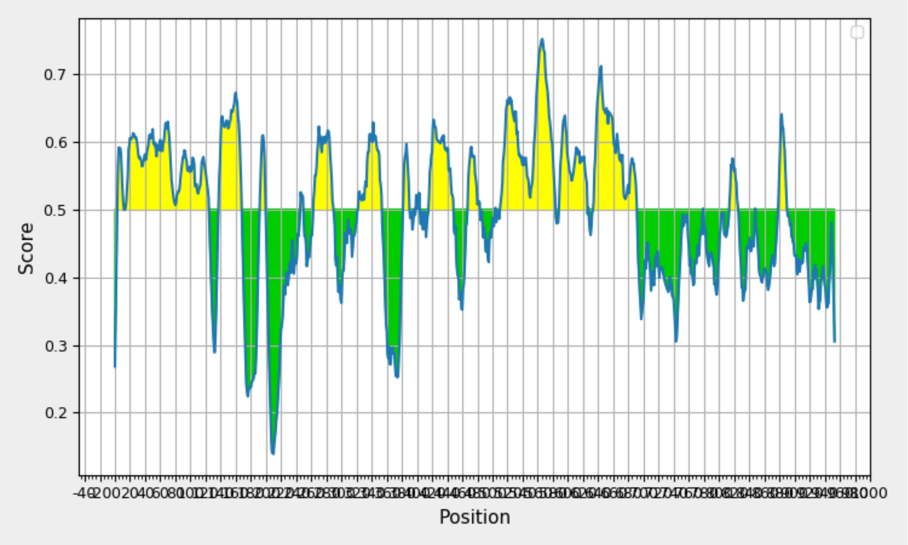

			LSGVTAVDGGPPEALRALTGEDQQQQ QQQQQQHQQSLQSPDNNMDDDE	
15	589	626	IALPLKRNSMSREERIARMKE ERARQAMVREKLDANTH	38
16	628	628	L	1
17	635	692	IKLRPRKKASSNGARLPLTRR DSCLATRRRALWPRKSSTAKLQL PSTLHRAPLSPTNS	58
18	780	780	**N**	1
19	815	826	MDIDQEHLAIPD	12
20	849	849	N	1
21	880	891	QHTNVDKPDQNV	12

Journal of Fungi

Protein name: Uncharacterized protein (14-3-3 Superfamily)

Predicted peptides: XP_002791205 [Pb01]

Bepipred Linear Epitope Prediction 2.0

No.	Start	End	Peptide	Length
1	17	21	ERYEE	5
2	31	31	**S**	1
3	33	40	DKELSVEE	8
4	57	57	A	1
5	64	80	SIEQKEESKGNEAQVAL	17
6	82	87	**KEYRQN**	6
7	108	114	**IASAETG**	7
8	138	145	DKRKDSAD	8
9	149	149	E	1
10	159	165	**QTDLAPT**	7
11	187	189	**PDQ**	3
12	191	191	C	1
13	206	215	**DTLSEESYKD**	10
14	230	262	WTSSEAEPAPEVAPAPAEQ KDEAPASTSEDAPK	33

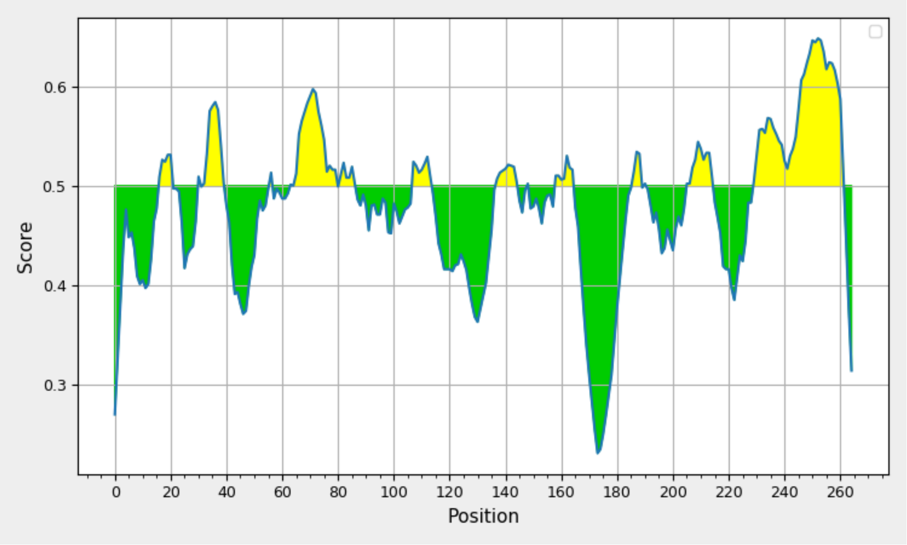

Predicted peptides: XP_010759842 [Pb18]

No.	Start	End	Peptide	Length
1	10	10	**S**	1
2	13	18	KELSVE	6
3	35	36	RA	2
4	38	38	W	1
5	45	59	EQKEESKGNEAQVAL	15
6	61	66	**KEYRQN**	6
7	87	93	**IASAETG**	7
8	117	123	DKRKDSA	7
9	138	144	**QTDLAPT**	7
10	166	168	**PDQ**	3
11	185	194	**DTLSEESYKD**	10
12	209	241	WTSSEAEPAPEVAPAPSEQKDEA PAATSEDAPK	33

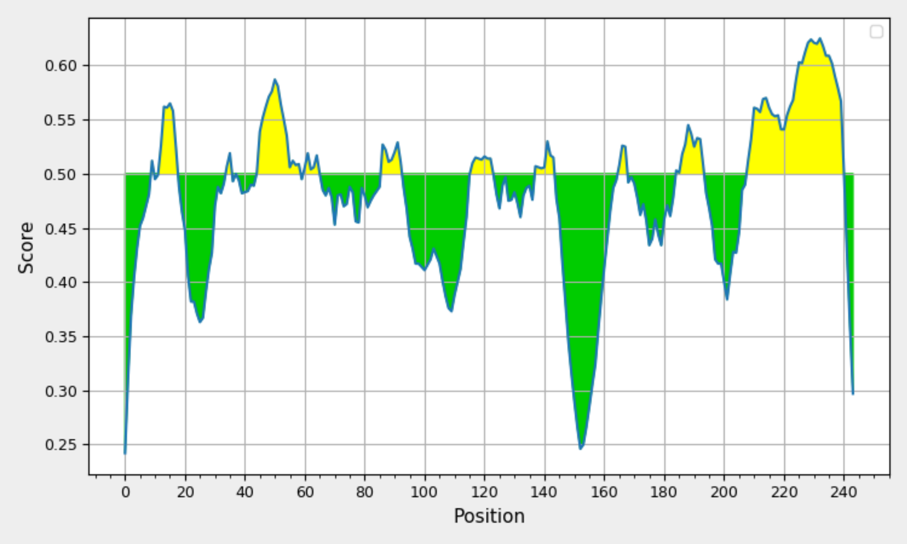

Journal of Fungi

Protein name: 14-3-3 family protein epsilon

Predicted peptides: XP_002796914 [Pb01]

No.	Start	End	Peptide	Length
1	5	6	RE	2
2	19	23	**ERYDE**	5
3	33	33	N	1
4	36	41	GELTVD	6
5	69	82	**QKEDSKGSEKHVAI**	14
6	85	86	**EY**	2
7	88	89	**QK**	2
8	110	116	**IPKAESG**	7
9	140	141	**NK**	2
10	143	143	**K**	1
11	161	167	QTELTPT	7
12	189	191	PDR	3
13	207	217	**LDSLSEESYRD**	11
14	233	289	TSSDGAEPEPVAAGDGKDDKTAEGEEG PATATTTDAPAPEEVAAPAAADEEPKKEGT	57

Bepipred Linear Epitope Prediction 2.0

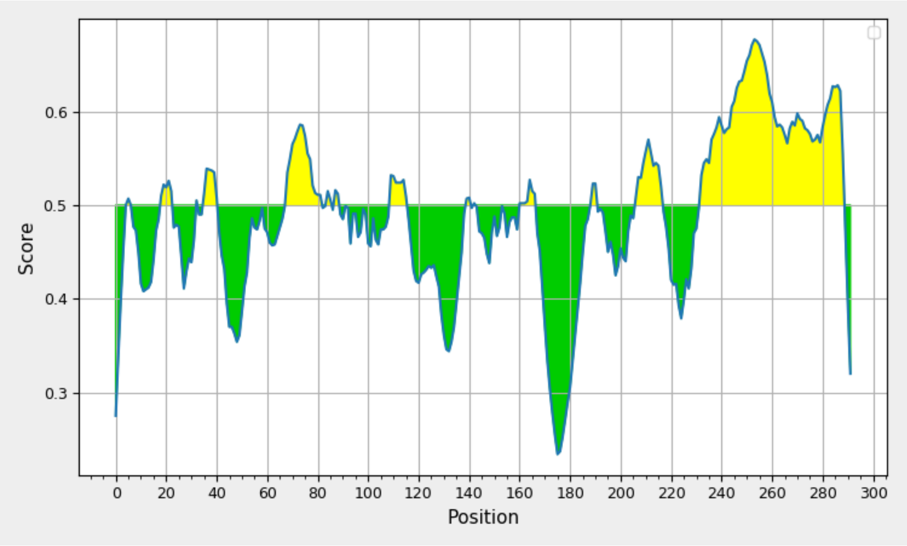

Predicted peptides: XP_010759301 [Pb18]

No.	Start	End	Peptide	Length
1	8	28	LPPIHSSGVCRANYNLSYSVK	21
2	41	45	**ERYDE**	5
3	55	63	NLGGELTVD	9
4	91	104	**QKEDSKGSEKHVAI**	14
5	107	108	**EY**	2
6	110	111	**QK**	2
7	132	138	**IPKAESG**	7
8	162	163	**NK**	2
9	165	165	**K**	1
10	176	176	K	1
11	183	183	Q	1
12	187	187	T	1
13	212	213	DR	2
14	229	239	**LDSLSEESYRD**	11
15	255	311	TSSDGAEPEPAAAGDGKDDKTAEGEEGS ATATTADPPAPEEMAAPAAADEEPKKEGT	57

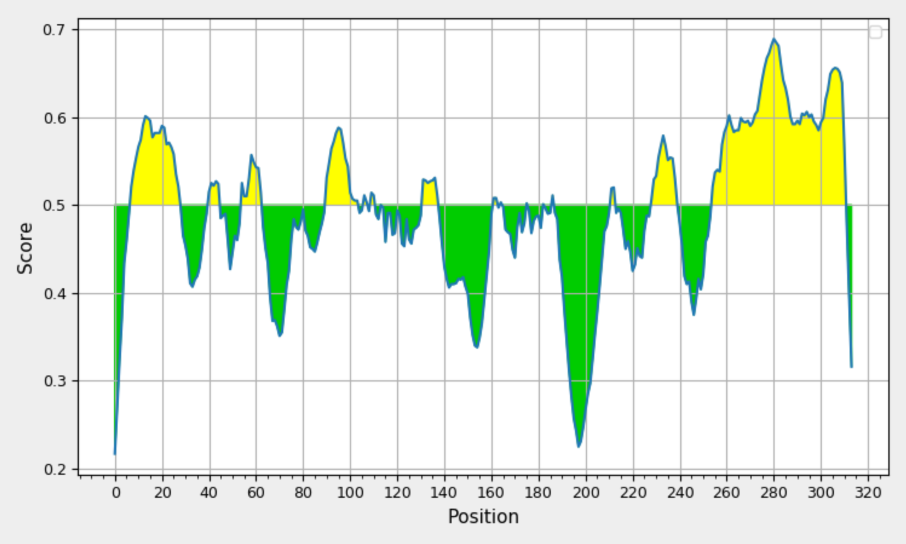

Journal of Fungi

Protein name: Spermidine synthase

Predicted peptides: XP_002793533 [Pb01]

No.	Start	End	Peptide	Length
1	5	10	THPTIK	6
2	18	24	SDMWPGQ	7
3	35	39	HHEKS	5
4	65	71	TERDEFS	7
5	108	110	ETV	3
6	128	139	**KYLPGMSVGFQH**	12
7	171	179	EGPAEALFQ	9
8	203	208	QWLHLS	6
9	218	219	RE	2
10	246	271	**KDSSRDVREPVRTWTREEEEKLCRYY**	26
11	273	273	K	1

Bepipred Linear Epitope Prediction 2.0

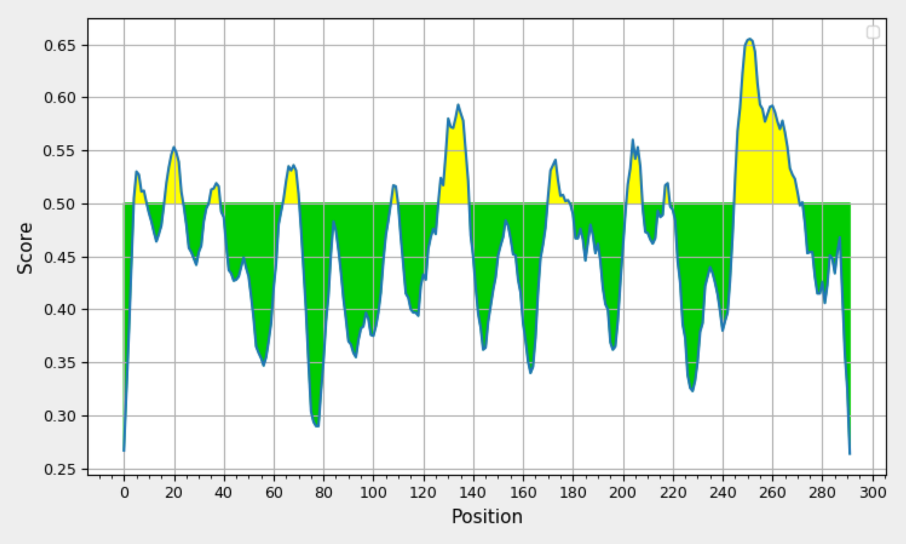

Predicted peptides: XP_010760066 [Pb18]

No.	Start	End	Peptide	Length
1	17	20	HEKS	4
2	47	51	ERDEF	5
3	89	92	ETVE	4
4	109	120	**KYLPGMSVGFQH**	12
5	152	156	EGPAE	5
6	183	191	ENQWLHLSL	9
7	200	201	**RE**	2
8	228	**253**	**KDSSRDVREPVRTWTREEEEKLCRYY**	26

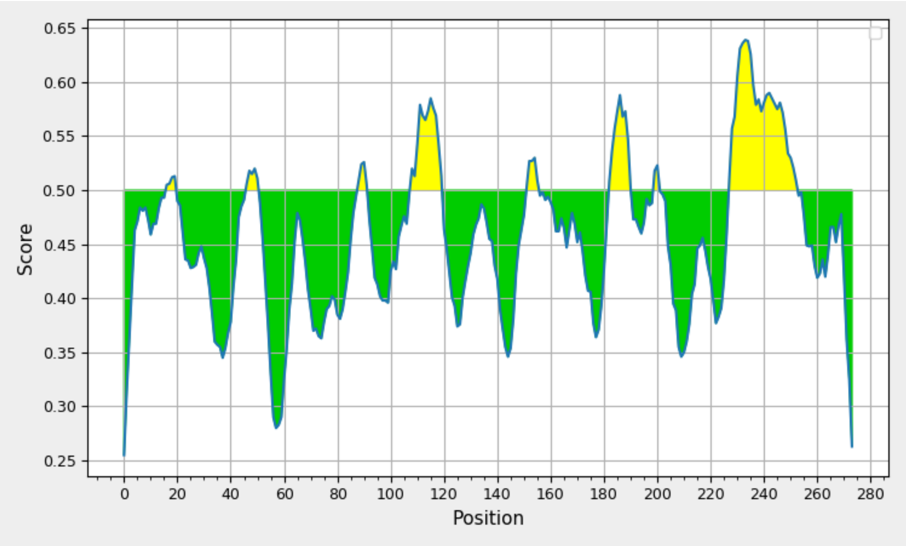

Journal of Fungi

Protein name: Phosphomannomutase

Predicted peptides: XP_002797030 [Pb01]

No.	Start	End	Peptide	Length
1	5	15	AGVYPPLEARP	11
2	29	39	LTPARGTVAPE	11
3	61	75	LAKQQEQLGTQTTDV	15
4	97	97	M	1
5	100	113	NSFIKWLGEEKYQN	14
6	126	137	KLPRKRGTFVEY	12
7	146	168	PVGRSASVEERNEFEEFDKVHNI	23
8	178	182	KEFAD	5
9	214	220	EKNISGV	7
10	231	247	KCFKGGNDHEIYEDSRT	17
11	256	257	DD	2
12	259	260	MR	2

Bepipred Linear Epitope Prediction 2.0

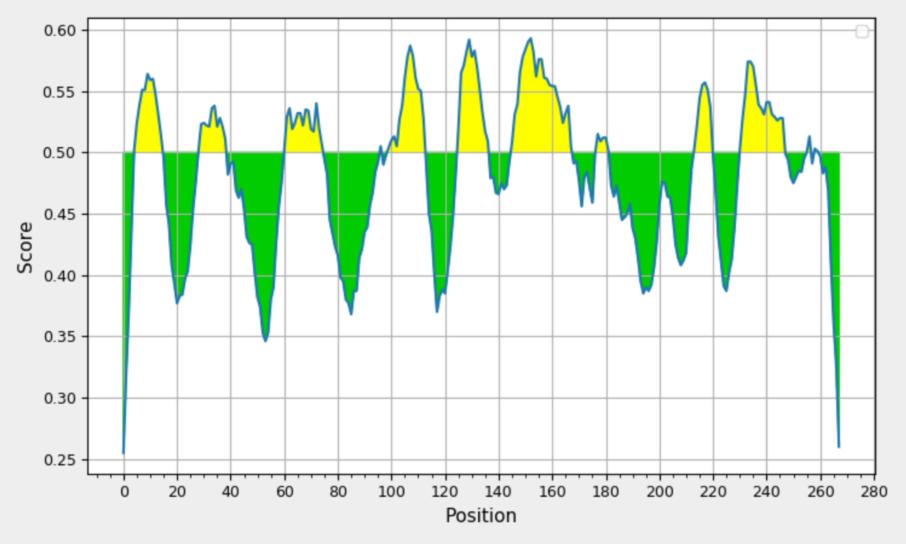

Predicted peptides: XP_010759656 [Pb18]

No.	Start	End	Peptide	Length
1	5	16	AGVYPPLEARPL	12
2	30	39	TPARGTVAPE	10
3	62	67	AKQQEQ	6
4	69	70	GT	2
5	73	75	TDV	3
6	99	113	SNSFIKWLGEEKYQN	15
7	122	168	ISSVKLPRKRGTFVEYRNGMVNISPVG RSASVEERNEFEEFDKVHNI	47
8	177	182	RKEFAD	6
9	215	220	KNISGV	6
10	232	249	CFKGGNDHEIYEDSRTIG	18
11	255	261	PDDTMRQ	7

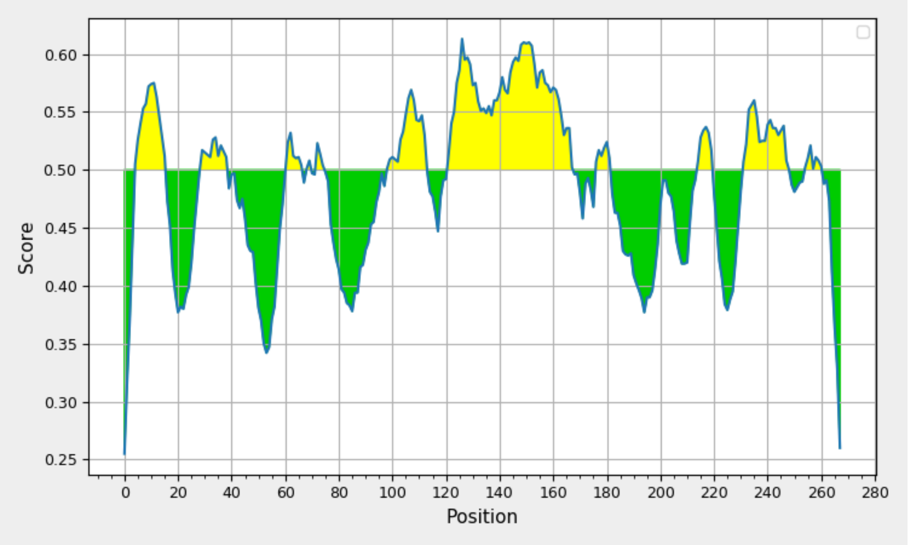

Journal of *Fungi*

Protein name: 4-hydroxyphenylpyruvate dioxygenase

Predicted peptides: XP_002789912 [Pb01]

No.	Start	End	Peptide	Length
1	5	25	AVSPPPSTRGTQQHNDLSSYK	21
2	54	63	AYRGLETGSR	10
3	84	102	RSLDDLSQFSPEEQALLTE	19
4	108	**109**	**EK**	2
5	139	149	VSGIKTLEDCQ	11
6	173	197	YNGIFLPGYREENTKDPLSSLLPPV	25
7	212	240	EEMEKVCEYYEKALGFHRFWSVDDKDICT	29
8	266	280	**KGKKQSQIEEYVNFY**	15
9	309	338	FIKVPETYYTEMKQRLERSGMKLKEDFEML	30
10	376	383	**NFSGFGAG**	8
11	394	399	**REQALR**	6

Bepipred Linear Epitope Prediction 2.0

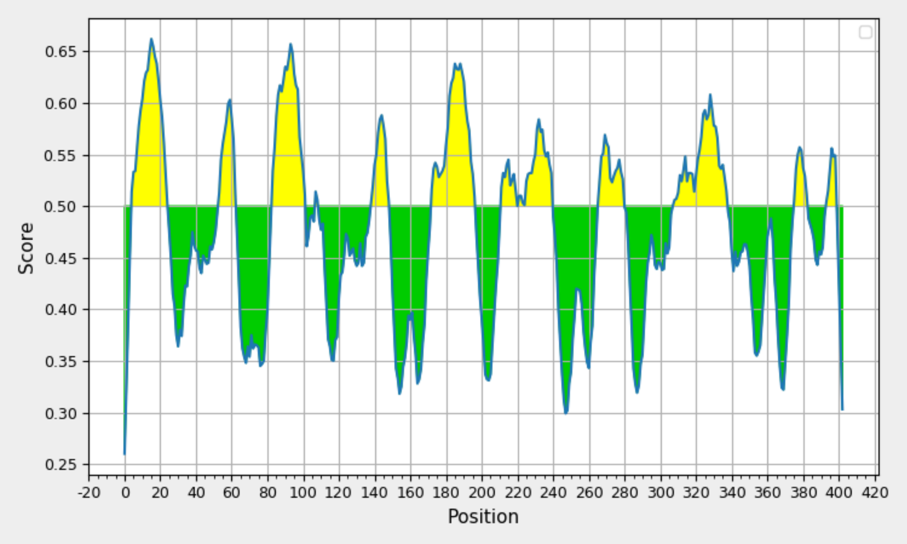

Predicted peptides: XP_010763814 [Pb18]

No.	Start	End	Peptide	Length
1	5	25	AVSPPPTTRDTQQHNNDLSSY	21
2	54	64	IAYRGLETGSR	11
3	85	103	RSLDDLSQFSPEEQTLLTE	19
4	106	107	SH	2
5	109	110	**EK**	2
6	141	150	SSIKTLEDSQ	10
7	174	198	YKGIFLPGYREEHTKDPLSSLLPPV	25
8	213	221	EEMEKVCEY	9
9	224	224	K	1
10	227	241	GFHRFWSVDDKDICT	15
11	267	281	**KGKKQSQIEEYVNFY**	15
12	309	339	EFIKVPETYYTEMKQRLERSGMKLKEDFEML	31
13	377	384	**NFSGFGAG**	8
14	395	400	**REQALR**	6

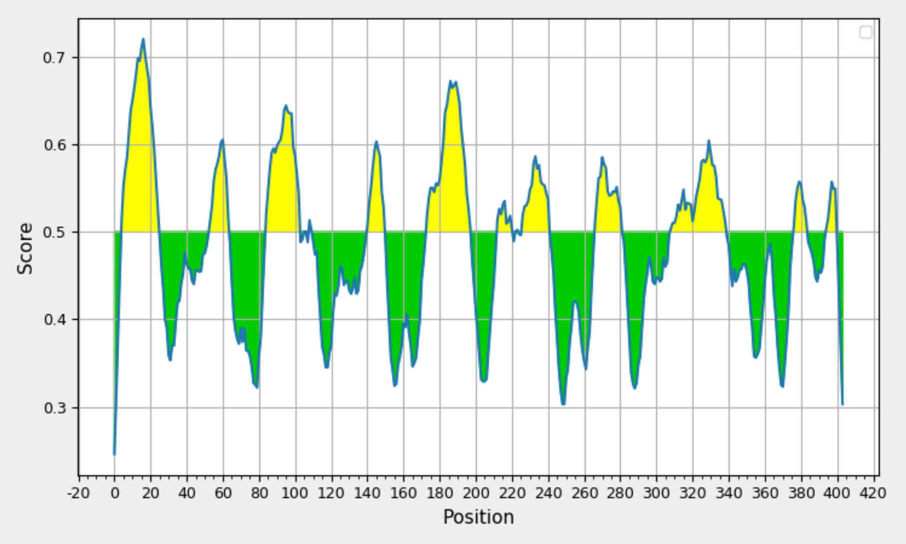

Journal of Fungi

Protein name: Uncharacterized protein (Cyclophilin superfamily)

Predicted peptides: XP_015701903 [Pb01]

No.	Start	End	Peptide	Length
1	5	27	TGTSSDSLPSNHTRSPRDALERR	23
2	65	79	TGEKGEGKSGKLLSY	15
3	98	123	FTAFNGTGGESIYGEKFDDENFDLKH	26
4	175	185	KSQTDTSDKPY	11
5	194	258	GQLSGDSYEAAKEVSVDETGDKY EDYPEDAGETLSGAEYYKIAS EVKEFGNKAFKSGNLDLGLEK	65
6	260	261	QK	2
7	267	282	NECPEPSDSDPPELQG	16
8	304	308	**RFDDA**	5
9	321	329	AAQAKDTDR	9
10	344	350	**KDDEAAL**	7
11	360	365	**APGDAA**	6
12	367	368	**VS**	2
13	371	388	ARVKKTIAEQQRKEKEML	18

Bepipred Linear Epitope Prediction 2.0

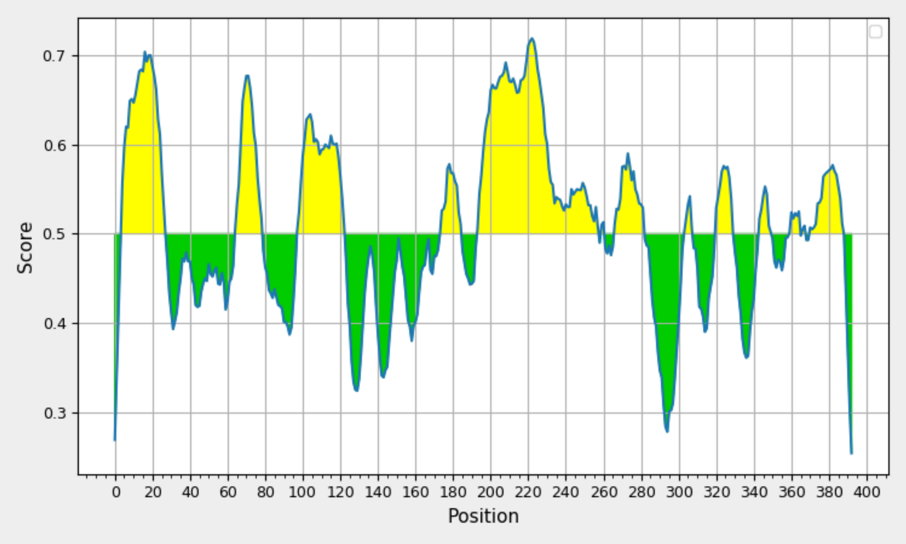

Predicted peptides: XP_010761900 [Pb18]

No.	Start	End	Peptide	Length
1	45	59	TGEKGEGKSGKQLSY	15
2	79	103	TAFNGTGGESIYGEKFDDENFDLKH	25
3	155	165	KSPTDTSDKPH	11
4	175	237	QLSGDSYEAAKEVSVDETGDKYED YPEDAGETLSGAEYYKIASEVK EFGNKAFKSGNLDLGLE	63
5	248	262	ECPEPSDSDPPELQG	15
6	284	288	**RFDDA**	5
7	299	310	ATAAQAKDTDRA	12
8	324	330	**KDDEAAL**	7
9	340	345	**APGDAA**	6
10	347	348	**VS**	2
11	351	368	ARVKKSIAEQQRKEKEML	18

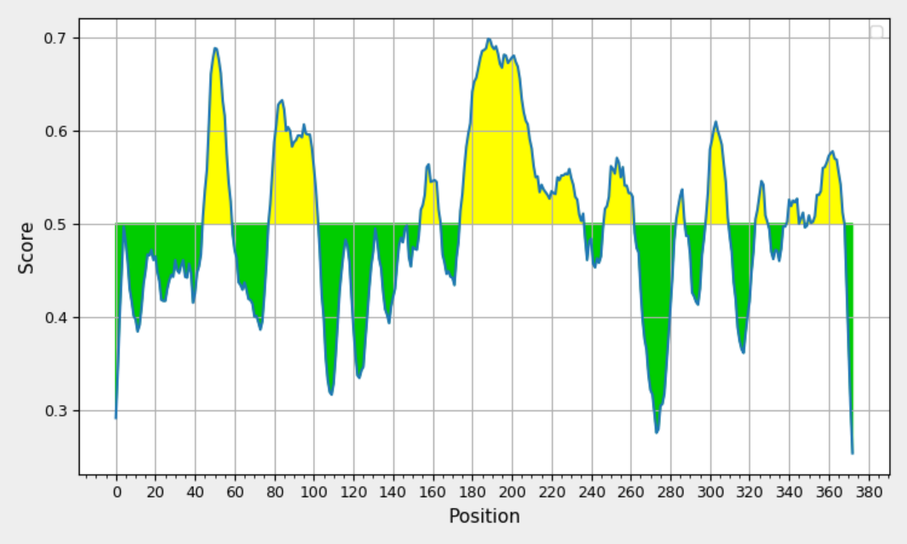

Journal of Fungi

Protein name: Thioredoxin reductase

Predicted peptides: XP_002791109 [Pb01]

No.	Start	End	Peptide	Length
1	36	42	MANGTAA	7
2	52	63	VENFPGFPHGIG	12
3	90	111	**VDLSCRPFKLWKEFSDGPDDAP**	22
4	126	139	ARRLDLPGEQQYWQ	14
5	151	156	AVPIFR	6
6	192	197	**LRASKT**	6
7	224	224	**A**	1
8	234	242	IKNVVSGVE	9
9	260	262	TAL	3
10	268	268	E	1
11	283	283	Y	1
12	322	355	AESEGGDEPPFVATSIEQSNQ GENAPPTLEYTSN	34

Bepipred Linear Epitope Prediction 2.0

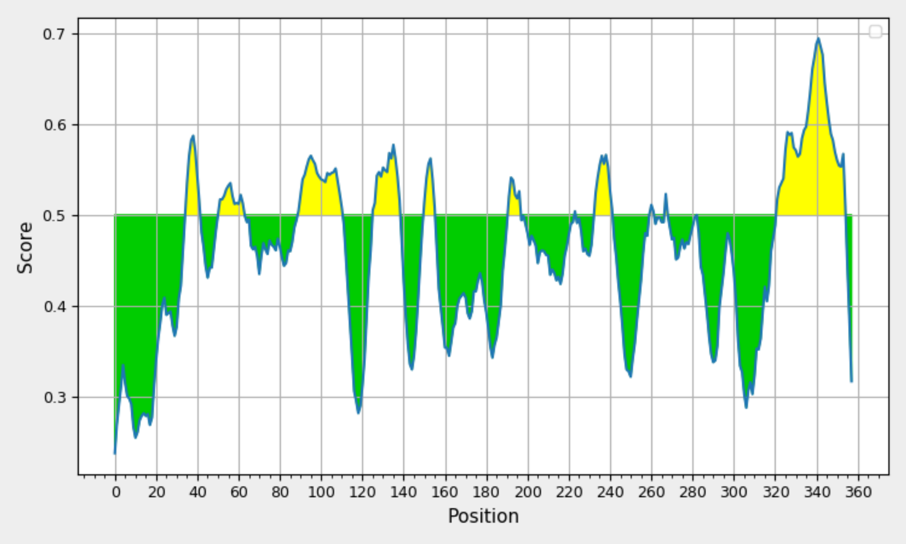

Predicted peptides: XP_010756960 [Pb18]

No.	Start	End	Peptide	Length
1	35	43	MMANGTAAG	9
2	51	63	DIENFPGFPNGIG	13
3	90	111	**VDLSCRPFKLWKEFSDGPDDAP**	22
4	127	139	RRLDLPGEQQYWQ	13
5	150	156	GAVPIFR	7
6	192	197	**LRASKT**	6
7	199	199	A	1
8	224	224	**A**	1
9	234	242	IKNVVSGGE	9
10	258	258	P	1
11	260	269	TALVKGQIET	10
12	322	355	AESEGGDEPPLVATSIEQSNQG ENAPPTLEYTSN	34

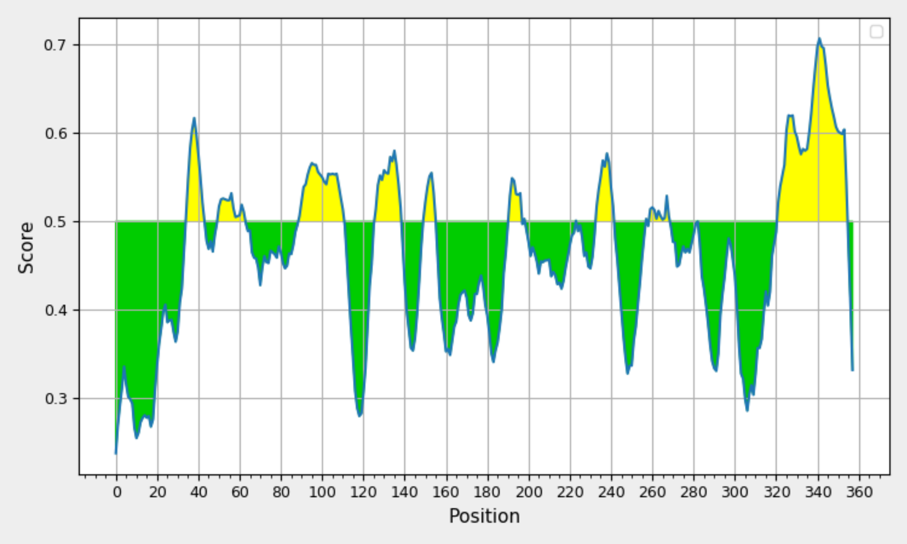

Journal of *Fungi*

Protein name: ATP synthase subunit beta

Predicted peptides: XP_002789970 [Pb01]

No.	Start	End	Peptide	Length
1	5	6	RY	2
2	18	33	LAANPIAQRPSESRFE	16
3	46	69	LTKIRSSHPHFLDSLTRNLAPHPV	24
4	84	141	SPFRQVPPPLLHITSIMFKSGVART FARSALARSSLRLPQNPALRSLASRYASTSSGQ	58
5	160	163	**EQLP**	4
6	198	204	**GTEGLVR**	7
7	214	218	PIMIP	5
8	234	265	PIDERGPIKHSKKAPIHAEAPEFVEQSTTAEV	32
9	294	294	**G**	1
10	323	328	**TREGND**	6
11	337	343	RVIQLDG	7
12	357	357	P	1
13	378	378	E	1
14	405	423	GRIPSAVGYQPTLAVDMGG	19

Bepipred Linear Epitope Prediction 2.0

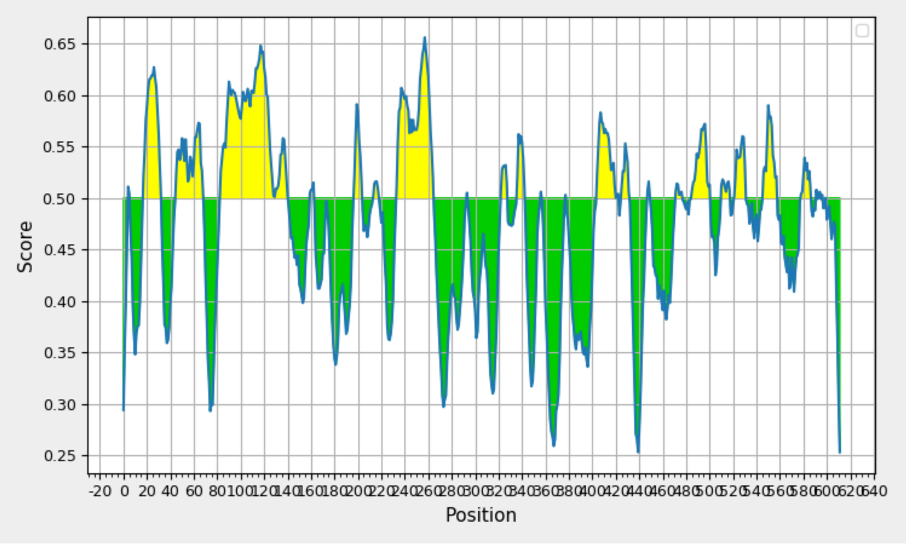

15	426	432	**ERITTTT**	7
16	448	450	DLT	3
17	472	477	SELGIY	6
18	486	501	KSRMLDPRIVGEEHYE	16
19	511	518	**QEYKSLQD**	8
20	522	531	**ILGMDELSEA**	10
21	547	558	SQPFTVAQVFTG	12
22	579	587	GEGDDLPEA	9
23	592	592	V	1
24	594	595	DF	2
25	597	597	S	1

Predicted peptides: XP_010763627 [Pb18]

No.	Start	End	Peptide	Length
1	8	30	RTFARSALARHSLRLPQNPALRS	23
2	33	41	SRYASTSSG	9
3	61	64	**EQLP**	4
4	99	105	**GTEGLVR**	7
5	116	119	IMIP	4
6	134	167	DPIDERGPIKHSKKAPIHAEAPEFV EQSTTAEVL	34
7	195	195	**G**	1
8	224	229	**TREGND**	6
9	237	244	TRVIQLDG	8
10	258	259	PP	2
11	279	279	**E**	1
12	306	320	GRIPSAVGYQPTLAV	15
13	327	333	**ERITTTT**	7
14	349	350	DL	2
15	373	379	SELGIYP	7
16	385	402	DSKSRMLDPRIVGEEHYE	18

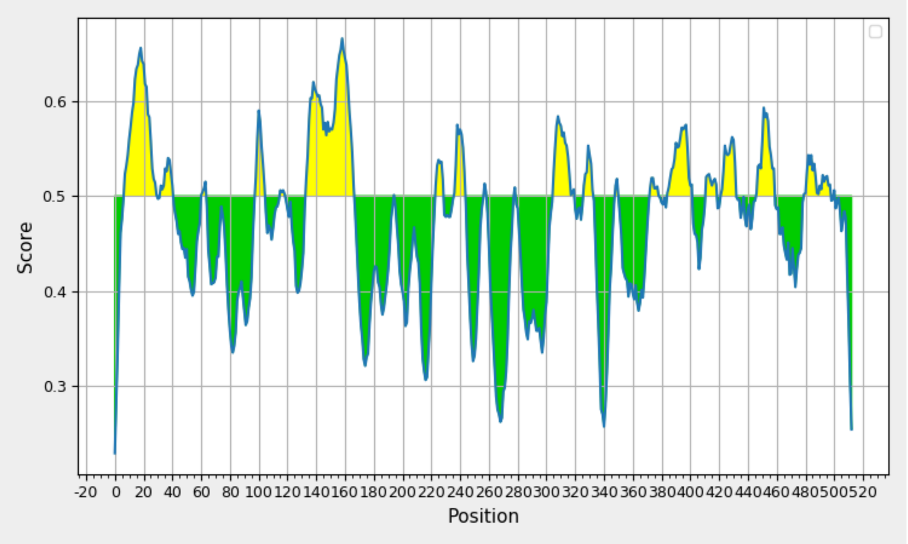

17	412	419	**QEYKSLQD**	8
18	423	432	**ILGMDELSEA**	10
19	447	459	LSQPFTVAQVFTG	13
20	480	498	GEGDDLPEAAFYMVGDFES	19
21	501	501	A	1

Journal of Fungi

Protein name: Hexokinase

Predicted peptides: XP_002791907 [Pb01]

No.	Start	End	Peptide	Length
1	5	13	SSSSSVLDE	9
2	23	30	YSAEELNR	8
3	37	37	**R**	1
4	40	52	**HEGLEEEGATLRQ**	13
5	54	55	PT	2
6	58	65	TAVPEGTE	8
7	105	110	LMTTKN	6
8	129	154	HQSEHFDLHIKKRQTGQITAPYTDDH	26
9	168	173	QHGINS	6
10	181	191	KGFDIKEAVGK	11
11	230	231	**GP**	2
12	254	274	RVTKLDKKNGDSAAAKYDRAT	21
13	287	294	**DNHLSVLP**	8
14	304	313	NDSVNPGIQM	10
15	339	370	AVKLFSNDSAARQNGLVTIIPADSGLHKQWGI	32

Bepipred Linear Epitope Prediction 2.0

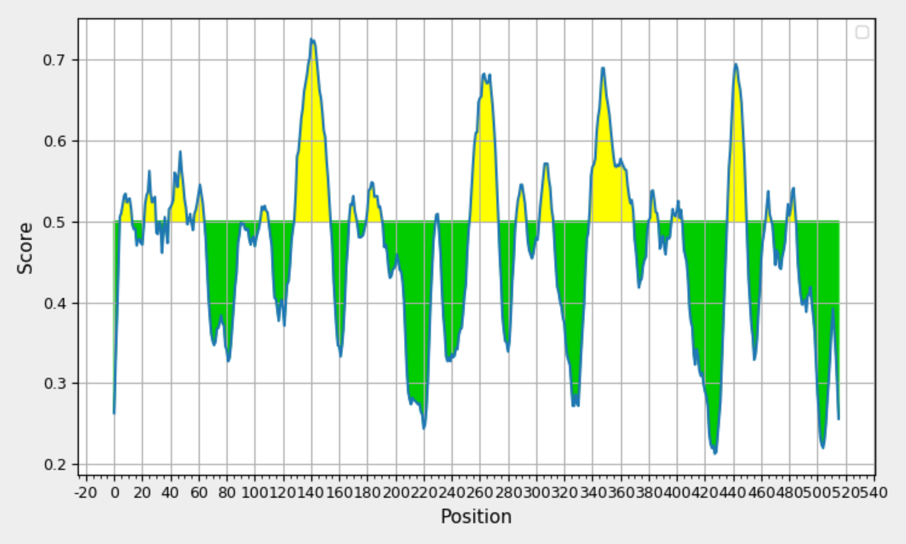

16	381	387	DDSPAFD	7
17	398	404	**GIENAST**	7
18	437	450	GRLRGEGEVEKGDG	14
19	465	468	PGFE	4
20	480	485	**GIGENG**	6

Predicted peptides: XP_010763342 [Pb18]

No.	Start	End	Peptide	Length
1	5	13	SSSSPVLDE	9
2	23	30	YPAEELNR	8
3	33	34	RE	2
4	37	37	**R**	1
5	40	52	**HEGLEEEGATLRQ**	13
6	55	55	T	1
7	58	64	TAVPEGT	7
8	109	110	KN	2
9	130	155	QSEHFDLHIKKRQTGEITAPYTDDHI	26
10	169	172	HGIN	4
11	182	191	GFDIKEAVGK	10
12	230	231	**GP**	2

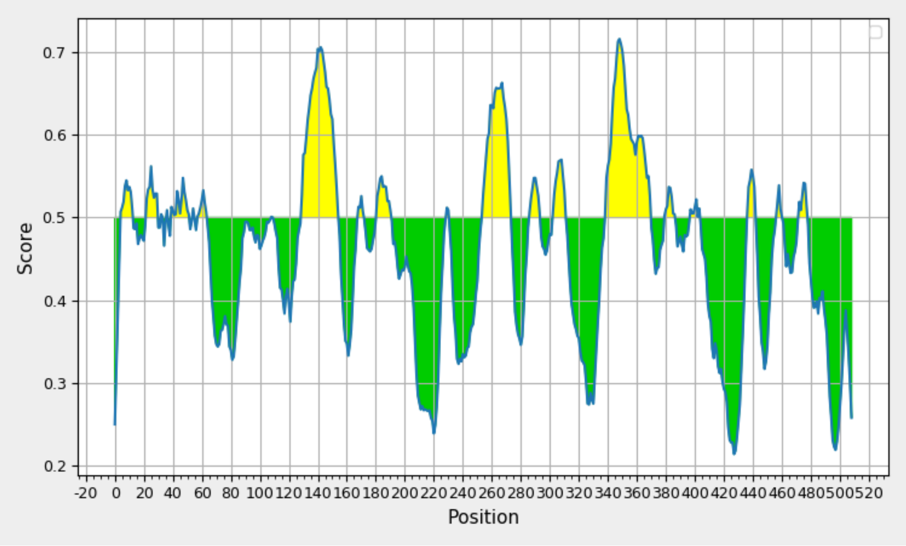

13	254	274	RVTKLDKKNGNSADAKYDRAT	21
14	287	294	**DNHLSVLP**	8
15	303	312	DNDSVNPGIQ	10
16	339	370	AVMLFNNDSAAGQNGLVTIIP ADSDLHKQWGI	32
17	380	387	ADDSPAFD	8
18	398	404	**GIENAST**	7
19	437	442	DRLRGD	6
20	457	461	YPGFE	5
21	473	478	**GIGENG**	6

Journal of Fungi

Protein name: Hsp75-like protein

Predicted peptides: XP_015700721 [Pb01]

No.	Start	End	Peptide	Length
1	5	6	**EV**	2
2	35	38	**NEQG**	4
3	61	68	NQAAMNPQ	8
4	79	102	GRRFDDPVVKKDVESWPFKVVDQG	24
5	138	138	**K**	1
6	140	140	**A**	1
7	154	159	FNDNQR	6
8	193	196	RSEK	4
9	251	267	**ERKTKKDLSGDPRALRR**	17
10	277	277	R	1
11	280	281	SN	2
12	330	334	**NIDKS**	5
13	356	369	SDFFNGKKLEKSIN	14
14	388	394	QATSADT	7
15	410	431	AMEGNIFAPVVPRGQTVPTIKK	22

Bepipred Linear Epitope Prediction 2.0

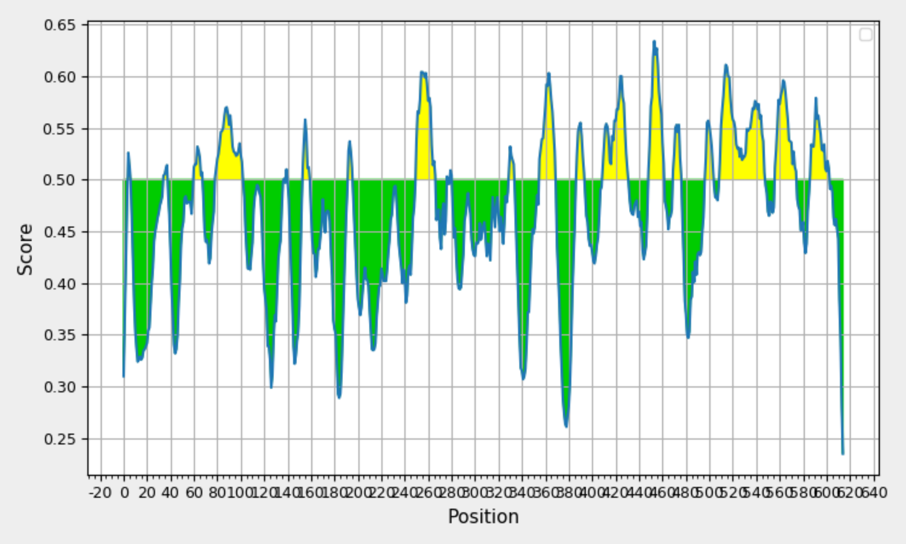

16	449	462	**YQGERTNCEDNTSL**	14
17	470	477	**IPPMKAGD**	8
18	498	504	EKSSGRS	7
19	510	548	SNAVGKLSSTEIENMINDAAR FKSSDEAFSKRFESRQQL	39
20	557	575	**EIISDPTMSLKIKRGNKEK**	19
21	587	603	**LEIEDSSPEDLKKKELA**	17

Predicted peptides: XP_010758595 [Pb18]

No.	Start	End	Peptide	Length
1	5	6	**EV**	2
2	35	38	**NEQG**	4
3	62	66	QAAMN	5
4	68	68	Q	1
5	79	103	GRRFDDPVVKKDVESWPFKVVDQGG	25
6	115	116	TK	2
7	138	138	**K**	1
8	140	140	**A**	1
9	154	160	FNDNQRQ	7
10	193	197	RSEKE	5

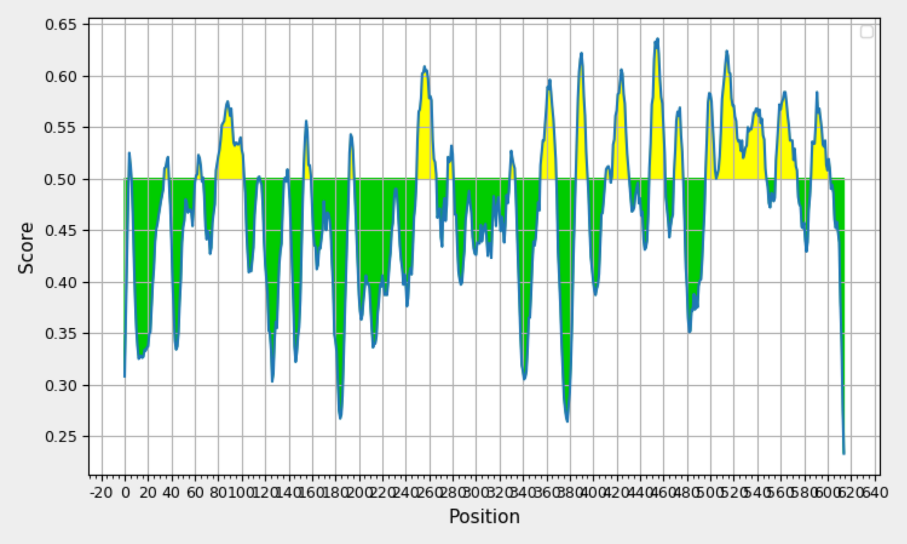

11	251	267	**ERKTKKDLSGDPRALRR**	17
12	277	281	RTLSN	5
13	330	334	**NIDKS**	5
14	356	369	**SDFFNGKKLEKSIN**	14
15	387	396	GQATSADTND	10
16	412	415	EGNI	4
17	417	431	APVVPRGQTVPTIKK	15
18	449	462	**YQGERTNCEDNTSL**	14
19	470	477	**IPPMKAGD**	8
20	497	548	TEKSSGRSANITISNAVGKLSSTEIE NMINDAAKFKSSDEAFSKRFESRQQL	52
21	557	575	**EIISDPTMSLKIKRGNKEK**	19
22	587	603	**LEIEDSSPEDLKKKELA**	17

Journal of Fungi

Protein name: (M20_dimer domain-containing protein)

Predicted peptides: XP_002789172 [Pb01]

No.	Start	End	Peptide	Length
1	6	26	LPISRIAASLPPPTRLRAAVQ	21
2	48	102	PSPYPSSSFYPLSIPRAVRPPSPSTSTVTRI HRRSFYANIDDPKMAPQLEPFFKE	55
3	104	109	DNLQDA	6
4	112	112	**D**	1
5	123	135	VSAQDDKRKDVVK	13
6	145	146	KR	2
7	149	150	**AE**	2
8	154	166	**RPLGKEHGREHLD**	13
9	195	204	**ALLEDGWNTE**	10
10	207	211	KLTVD	5
11	243	243	**V**	1
12	273	276	KKFF	4
13	290	292	LGT	3
14	317	326	**DLHSGVFGGS**	10

Bepipred Linear Epitope Prediction 2.0

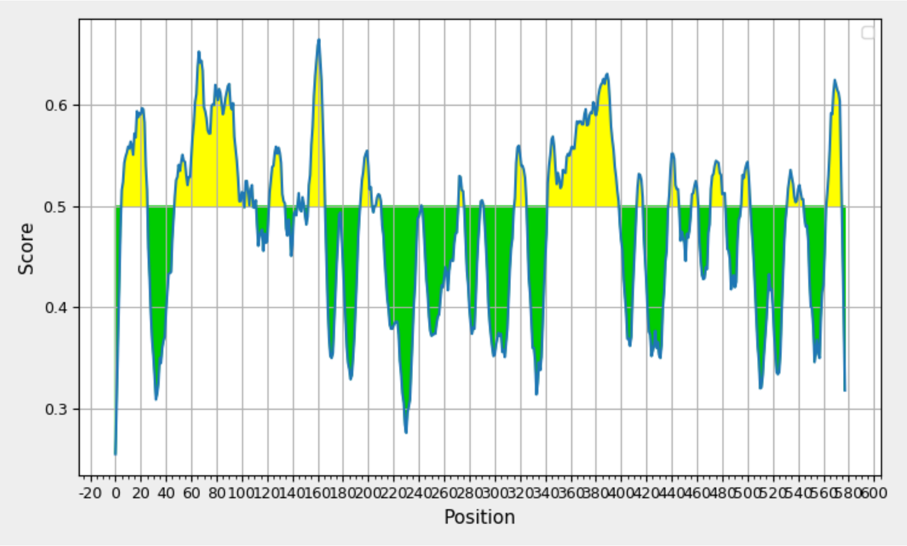

15	343	399	DVQGNILIPGINELVAPLTEDEKGLY KDIAFSMENLYESLGSTTSIFSDKEPTLMRR	57
16	414	418	YSAPG	5
17	439	446	**MESEDVNK**	8
18	457	462	**AKLNSK**	6
19	472	483	**DGKWWVASPKHW**	12
20	496	503	FGVEPDFT	8
21	533	545	**DMAHSTNEKLDKR**	13
22	564	575	EPIKEQINTDNV	12

Predicted peptides: XP_010761260 [Pb18]

No.	Start	End	Peptide	Length
1	9	24	AASLPPPIRLRRAAVQ	16
2	57	96	LSLPRTIRPPSPSTSTVIRIHRRSFYANI DDPKMAPQLEP	40
3	99	100	KE	2
4	102	104	DIL	3
5	106	107	DA	2
6	110	110	**D**	1
7	121	133	VSAQDDNRKDVVK	13

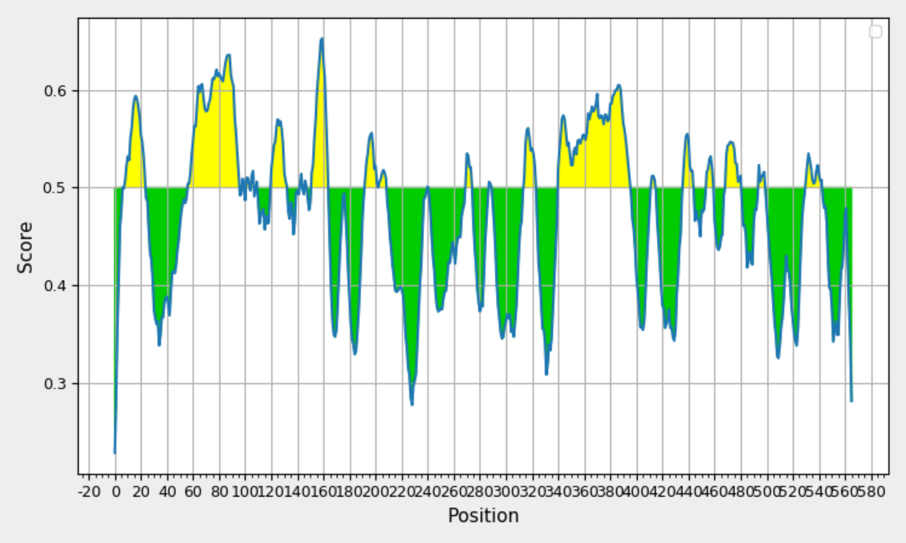

8	143	144	RR	2
9	147	148	**AE**	2
10	152	164	**RPLGKEHGREHLD**	13
11	193	202	**ALLEDGWNTE**	10
12	204	209	FKLTVD	6
13	241	241	**V**	1
14	270	274	SKKYF	5
15	288	290	**LGT**	3
16	315	324	**DLHSGVFGGS**	10
17	341	396	DVQGNILIPGINELVAPLTEDEKGLYKDIA FSMENLYESLGSTTSIFSEKEPTLMR	56
18	412	415	YSAP	4
19	437	444	**MESEDVNK**	8
20	455	460	**AKLNSK**	6
21	470	481	**DGKWWVASPKHW**	12
22	495	499	GVEPD	5
23	531	543	**DMAHSTNEKLDKR**	13

Journal of Fungi

Protein name: Acetyltransferase component of pyruvate dehydrogenase complex

Predicted peptides: XP_002797511 [Pb01]

No.	Start	End	Peptide	Length
1	6	6	P	1
2	16	24	**WQKKVGDAL**	9
3	55	63	REAGEKDIA	9
4	75	145	GTDITPFESFSLEDAGGEKSSALK EPEQPKKELKVAPAAPKEESTPAA EEEPVSTGERLQPSLDRESFIAP	71
5	156	200	VPLKDIKGTGPGGRVTKNDVEK YQPAGTAVSASGPAFEDIPASSM	45
6	202	203	KI	2
7	206	206	N	1
8	209	215	VQSMREN	7
9	238	245	NSADGKYK	8
10	305	309	**AHTLG**	5
11	328	333	**KLKPEE**	6
12	379	385	GEDGTSV	7
13	418	421	**VENP**	4

Bepipred Linear Epitope Prediction 2.0

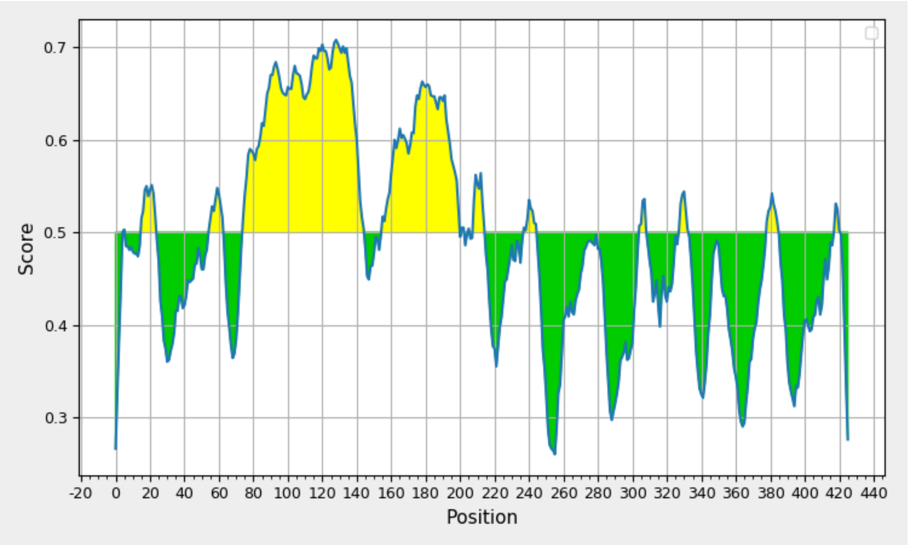

Predicted peptides: XP_010762465 [Pb18]

No.	Start	End	Peptide	Length
1	7	82	SRSASCRSLFSRSPPTCIRESGHMYRL RDASRYGQCSRIWLLSLMFTLILVV HRGRRQLPAFAALARFYASKSYPP	76
2	92	94	SPT	3
3	103	111	**WQKKVGDAL**	9
4	142	152	REAGEKDVTVG	11
5	159	233	VEEGTDITPFESFSLEDAGGEKAPTLK QPEQPKEELKVAPAAPKEESTPAAEE EPVSTGERLQPSLDRESFIAPA	75
6	239	239	L	1
7	243	285	VPLKDIKGTGPGGRVTKNDVEKY QPAGTAVSGPPYEDIPASSM	43
8	294	299	LQSMRE	6
9	326	328	NGK	3
10	330	330	K	1
11	390	394	**AHTLG**	5
12	413	418	**KLKPEE**	6
13	464	470	GEDSTSV	7
14	503	506	**VENP**	4

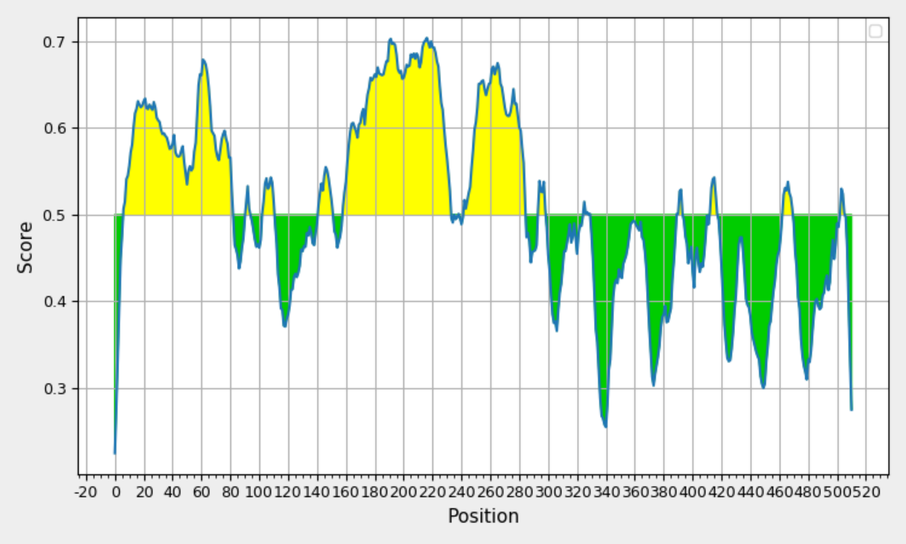

Protein name: Protein disulfide-isomerase

Predicted peptides: XP_002797127 [Pb01]

No.	Start	End	Peptide	Length
1	23	29	ADEAASD	7
2	57	60	**CGHC**	4
3	91	100	**ELCQEYGVEG**	10
4	111	124	EQVKPYSGPRKSSS	14
5	130	150	VKQSLPAVTVVTVDNLEDVKT	21
6	164	170	DKATNET	7
7	210	248	DEGKTVYKGELTQEQVTSFIKLSSTPL IGELGPHTYAGY	39
8	264	271	EEREEFSK	8
9	303	304	LK	2
10	318	334	NNKKYPFDQELKITHDT	17
11	337	337	**T**	1
12	339	378	VQDVLDGKVEPSIKSEPIPEKQEGP VTVVVARSYQELVID	40
13	392	395	CGHC	4
14	408	416	LYADNPEFA	9

Bepipred Linear Epitope Prediction 2.0

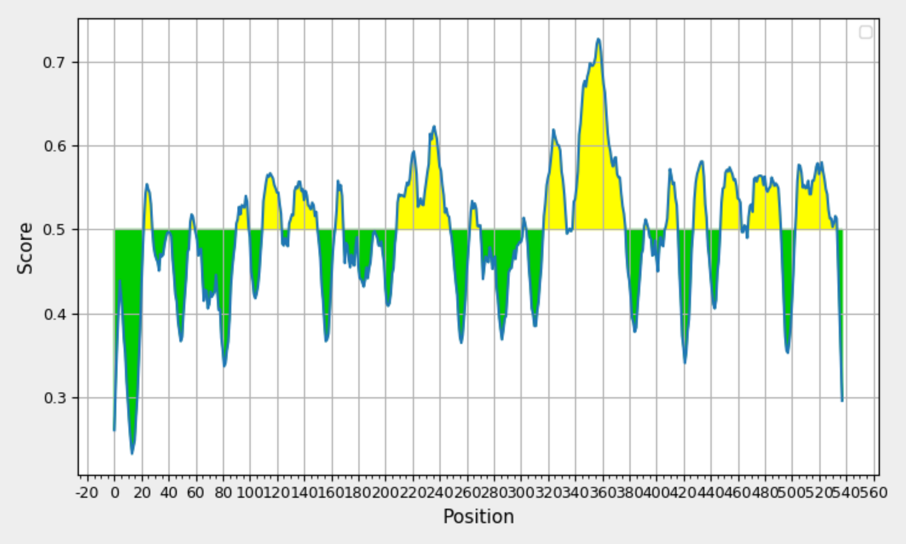

15	429	438	**NDVPEEIQGF**	10
16	449	463	KDKPFDYQGLRTIQG	15
17	466	467	DF	2
18	469	492	RDNGKHKVDAYDESKVNEDDDDDD	24
19	504	534	SSSSTKTAEKKKDSKTTSSAAPKDTAAESAR	31

Predicted peptides: XP_010759621 [Pb18]

No.	Start	End	Peptide	Length
1	22	30	AADEAAVSD	9
2	58	61	**CGHC**	4
3	92	101	**ELCQEYGVEG**	10
4	112	125	EQVKPYSGPRKSAS	14
5	132	149	KQSLPAVTVVTVDNLEDV	18
6	151	151	**T**	1
7	165	170	DKATNE	6
8	194	194	A	1
9	211	225	DEGKAVYSGELTQEQ	15
10	227	229	TSF	3
11	231	251	KLSSTPLIGELGPHTYARYIQ	21
12	264	272	PEEREEFSK	9

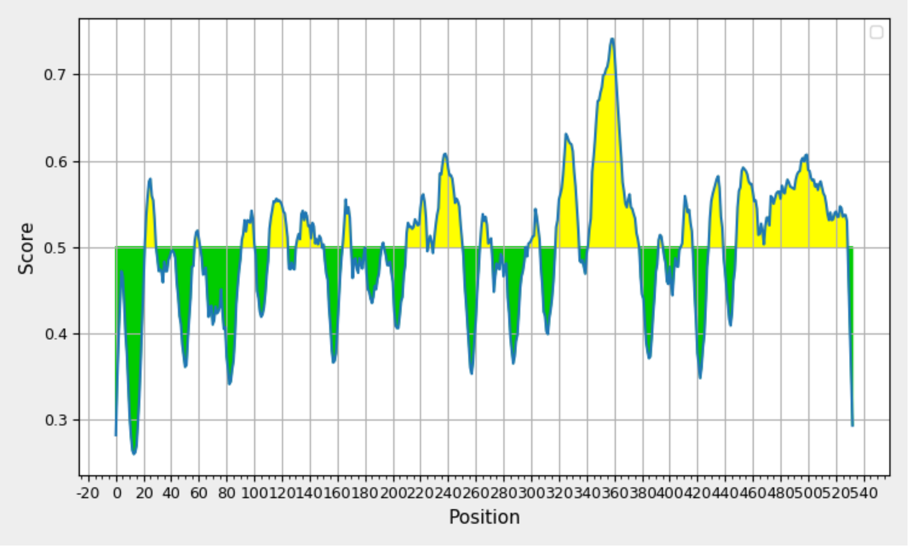

13	299	307	AGNLNLKVD	9
14	319	335	NNKKYPFDQELKITHDI	17
15	342	378	DVLDGKVEPSIKSEPIPEKQEGPV TVVVAHSYQELVI	37
16	393	396	CGHC	4
17	410	417	YADNPEFA	8
18	430	439	**NDVPEEIQGF**	10
19	449	529	SKDKPFDYQGSRTIQGLAEFVRDNGKH KVDAYDESKVTEDDDVTETPASPSSSSTKA ADEKKETKTTSSAAPKDTAAEGAR	81

Journal of Fungi

Protein name: HSP7-like protein

Predicted peptides: XP_015703361 [Pb01]

No.	Start	End	Peptide	Length
1	5	29	RFSRALPRAVPSFARSSSRSSAYKL	25
2	34	48	FRRWNSTEGGEEKVK	15
3	107	107	V	1
4	119	144	LIGRKFTDPECQRDLKEVPYKIVQHA	26
5	194	198	**NDSQR**	5
6	287	302	KKESGIDLSNDRMAIQ	16
7	316	321	SSALQT	6
8	330	337	**ADSSGAKH**	8
9	370	374	**NLQAK**	5
10	398	408	IFGRDPAKSVN	11
11	426	430	GEVTD	5
12	438	464	PLSLGIETLGGVFTRLINRNTTIPTKK	27
13	473	473	D	1
14	484	499	QGERELVRDNKLLGNF	16
15	502	512	VGIPPAHRGVP	11

Bepipred Linear Epitope Prediction 2.0

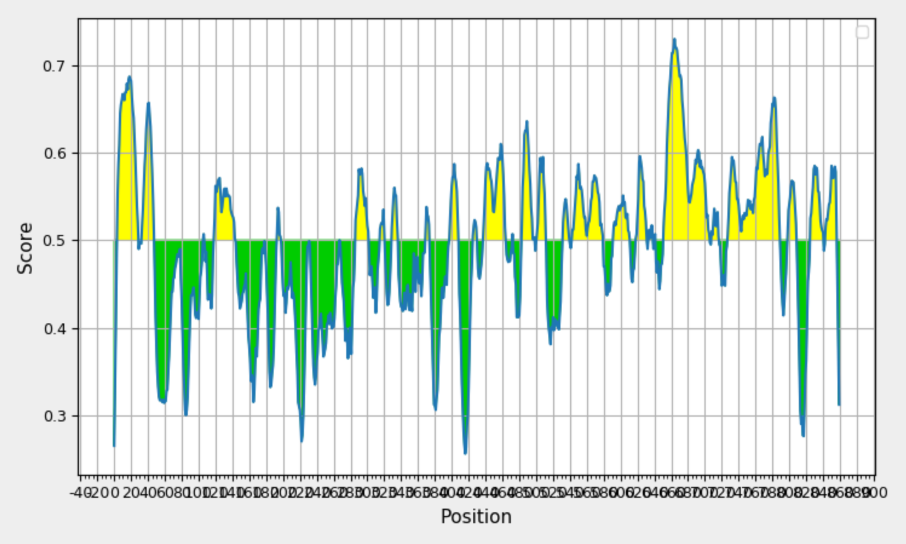

16	533	540	KSTGKDQS	8
17	544	580	ASGSGLSDTEIQNMVDDAEKFGAQD KERKAAIEAANR	37
18	592	611	LKEFEDKLDKTEADQIKEKI	20
19	619	632	**AKSQSGEGSITADE**	14
20	634	639	KAKVDE	6
21	641	642	QN	2
22	652	706	HKARSEEGQQQQSQQSNEGQQGDSNLL TETFDLIVSPANSFGRLDSAFNGAISRA	55
23	709	717	LPHHNYNSF	9
24	728	789	RWRGYAPPGSCTLVPFPAEI AWTAPWVHCVRLCGCRSVEGVE RGGRVQGEGDREGDGGERKR	62
25	799	809	VVGVGNVSKEK	11
26	826	841	ERTERWRRLEWSMLGR	16
27	844	857	FEVKKTWREKQKAW	14

Predicted peptides: XP_010756347 [Pb18]

No.	Start	End	Peptide	Length
1	5	29	RFSRALPRAVPSFARSSARSSAYKL	25
2	31	31	A	1
3	35	47	RRWNSTEGGEEKV	13
4	106	107	VV	2
5	120	138	IGRKFTDPECQRDLKEVPY	19
6	142	143	QH	2
7	179	179	G	1
8	194	198	**NDSQR**	5
9	230	232	REQ	3
10	267	268	TH	2
11	287	303	KKESGIDLSNDRMAIQR	17
12	315	321	LSSALQT	7
13	330	337	**ADSSGAKH**	8
14	370	374	**NLQAK**	5
15	398	409	IFGRDPAKSVNP	12
16	426	428	GEV	3
17	447	449	GGV	3

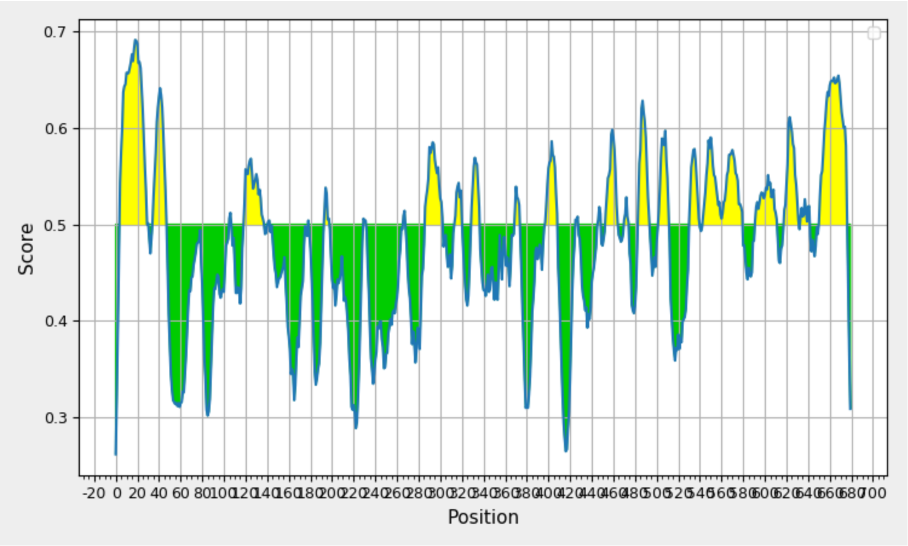

18	454	465	INRNTTIPTKKS	12
19	471	475	AADFQ	5
20	484	494	QGERELVRDNK	11
21	503	512	GIPPAHRGVP	10
22	531	540	KDKSTGKDQS	10
23	544	580	ASGSGLSDAEIQNMVDDAEKFG AQDKERKAAIEAANR	37
24	592	612	LKEFEDKLDKTEADQIKEKIA	21
25	619	632	**AKSQSGEGSITADE**	14
26	634	642	KAKVDELQN	9
27	651	677	MHKARSEDGQQQQSQQSNEGQQSGEGE	27

Journal of Fungi

Protein name: Uncharacterized protein (NBD sugar kinase HSP70 actin superfamily)

Predicted peptides: XP_002790451 [Pb01]

No.	Start	End	Peptide	Length
1	28	31	NEVS	4
2	55	60	QEISNL	6
3	72	91	GRSFNDPDVQIEQDYNSATL	20
4	93	97	DVNGE	5
5	106	110	GKKEQ	5
6	131	134	**ELKL**	4
7	147	152	FTDAQR	6
8	185	193	FDLPTAEEK	9
9	248	262	KEKFNIDIKTNLKAW	15
10	274	275	KI	2
11	277	278	SA	2
12	306	306	**E**	1
13	328	331	LKPE	4
14	354	366	**EFFGKPLSFTLNQ**	13
15	382	392	SPVFRVRDFSV	11

Bepipred Linear Epitope Prediction 2.0

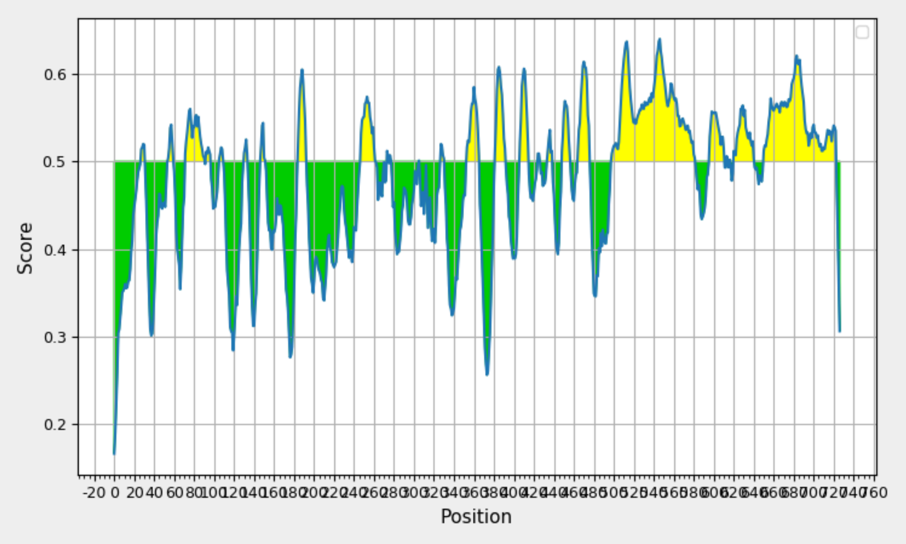

16	407	415	APDIPDEAT	9
17	425	427	VMP	3
18	434	439	FYRKQP	6
19	449	457	IDGLPGKTN	9
20	465	476	VKGITESSDGDF	12
21	499	582	VEDVEVEEPIPEKEGDAMDTDAPNG DAEDGKPKMRKVKKQVRKGDLPVSGGTA GLDPESKERLTEKENAMYMEDKLVADTEDKK	84
22	597	611	DGVYSEFASDEEKTK	15
23	614	614	**T**	1
24	617	617	D	1
25	621	641	**DWLYEDGEDTTKAVYISKMDD**	21
26	651	723	QRYTDKVEAERAAAQKAQEEAAAKR REEQEAAQEAAKKAAGPPPAADAEM KDMDAAAPAGEVDKNADESMAEV	73

Predicted peptides: XP_010758132 [Pb18]

No.	Start	End	Peptide	Length
1	5	8	DKLS	4
2	33	36	EISN	4
3	49	64	GRSFNDPDVQIEQDYN	16
4	71	73	VNG	3
5	83	88	GKKEQF	6
6	108	111	**ELKL**	4
7	125	127	TODA	3
8	163	170	DLPTAEEK	8
9	224	240	FKEKFNIDIKTNLKAWT	17
10	254	254	S	1
11	283	283	**E**	1
12	304	308	DLKPE	5
13	331	343	**EFFGKPLSFTLNQ**	13
14	360	370	PVFRVRDFSVH	11
15	383	392	RAPDIPDEAT	10
16	401	417	NVMPSTKILTFYRKQPF	17
17	426	433	IDRLPGKT	8

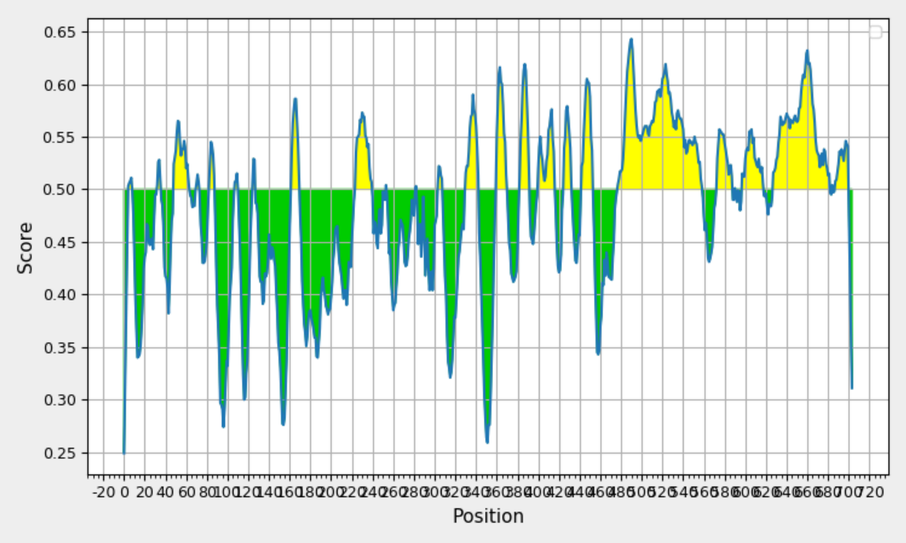

18	443	453	KGITESSDGDF	11
19	478	559	DVEVEEPIPEKEGDAMDTDAPNGDA EDGKPKMRKVKKQVRKGDLPVSGGTAGLD AESKERLTEKENAMYMEDKLVADTEDKK	82
20	573	588	IDGVYSEFASDEEKTK	16
21	591	591	**T**	1
22	598	618	**DWLYEDGEDTTKAVYISKMDD**	21
23	628	682	QRYTDKVEAERAAAQKAQEE AAAKRREEQEAAEEAAKKAA GPPPAADAEMKDMDA	55
24	685	685	P	1
25	688	700	EVDKNADESMAEV	13

Protein name: Aconitate hydratase, mitochondrial

Predicted peptides: XP_015699682 [Pb01]

No.	Start	End	Peptide	Length
1	6	23	RFARVGALASKSRNLLGA	18
2	31	56	ENPLDKKVEMCNYEKGNYINYKKMAE	26
3	66	74	NRPLTYAEK	9
4	81	92	DDPHEQDIHRGK	12
5	121	124	MPSV	4
6	139	156	QVGGEKDLARANEINKEV	18
7	278	283	VNSLSC	6
8	314	341	KATKRQAIGDFARSYAHSLREDEGAEYD	28
9	358	366	PFTPDLATP	9
10	372	380	DAVKENGWP	9
11	397	397	E	1
12	411	414	DHGI	4
13	436	440	DGQLK	5
14	461	470	WDRRDVKKGE	10
15	482	488	FTGRNDA	7

Bepipred Linear Epitope Prediction 2.0

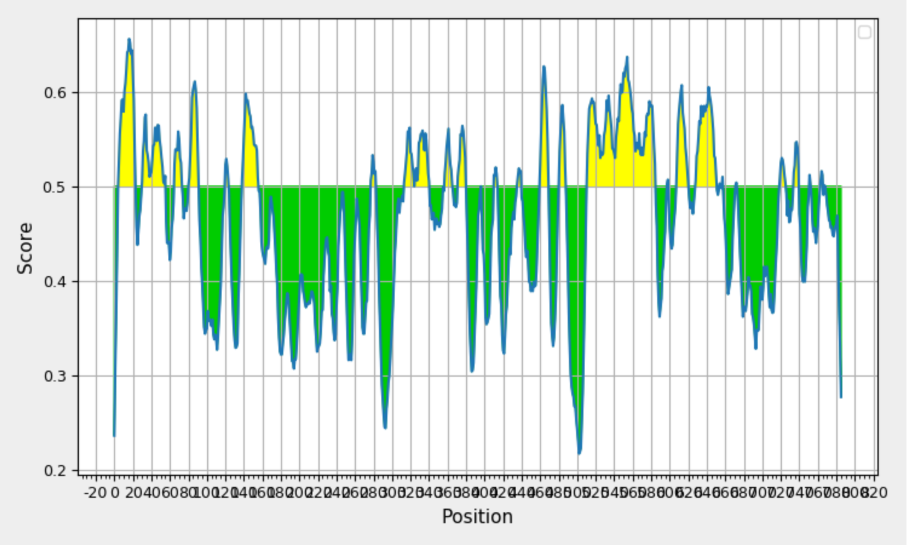

16	512	585	**NFNPLTDTLKDKDGKEFKLKEPTG AGLPANGYDPGRDTYQAPPKDRSSIS VAVSPTSDRLQILKAFQPWDGKDA**	74
17	598	599	**TT**	2
18	608	620	PWLKYRGHLDNIS	13
19	629	651	NAENGEANKVKNFKTGEYGPVPA	23
20	655	656	DY	2
21	658	658	A	1
22	672	673	GE	2
23	720	726	DYDRIRP	7
24	735	740	**TELAVG**	6
25	752	753	**GK**	2
26	764	766	**NEP**	3
27	768	769	LQ	2

Predicted peptides: XP_010760563 [Pb18]

No.	Start	End	Peptide	Length
1	6	15	DIVRSRLNRP	10
2	29	39	DPHEQDIHRGK	11
3	69	70	PS	2
4	86	105	QVGGEKDLARANEINKEVYD	20
5	226	230	NSLSC	5
6	255	255	R	1
7	261	288	**KATKRQAIGDFARSYAHSLREDEGAEYD**	28
8	305	316	PFTPDLATPISK	12
9	318	328	KDAVKENGWPE	11
10	358	361	**DHGI**	4
11	383	387	**DGQLK**	5
12	408	417	**WDRRDVKKGE**	10
13	429	435	**FTGRNDA**	7
14	459	532	**NFNPLTDTLKDKDGKEFKLKEPT GAGLPANGYDPGRDTYQAPPKDRSS ISVAVSPTSDRLQILKAFQPWDGKDA**	74
15	545	546	**TT**	2
16	555	566	PWLKYRGHLDNI	12

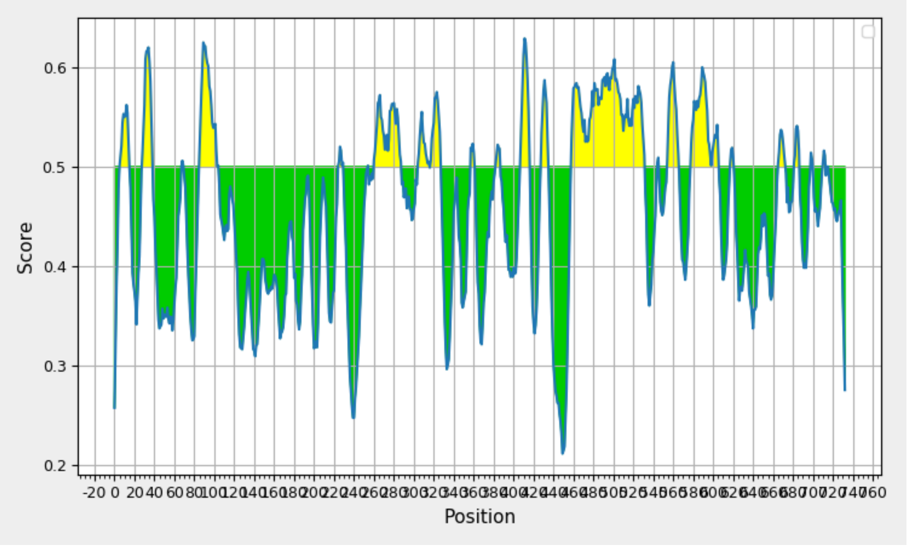

17	579	606	NGEANKVKNFKTGEYGPVPATARDYKAR	28
18	618	621	YGEG	4
19	666	673	ADYDRIRP	8
20	682	687	**TELAVG**	6
21	699	700	**GK**	2
22	711	713	**NEP**	3
23	716	716	Q	1

Reference

1. de Camargo, Z.P.; Unterkircher, C.; Campoy, S.P.; Travassos, L.R. Production of *Paracoccidioides brasiliensis* exoantigens for immunodiffusion tests. *J Clin Microbiol* **1988**, *26*, 2147-2151.
2. Gegembauer, G.; Araujo, L.M.; Pereira, E.F.; Rodrigues, A.M.; Paniago, A.M.; Hahn, R.C.; de Camargo, Z.P. Serology of paracoccidioidomycosis due to *Paracoccidioides lutzii*. *PLoS Negl Trop Dis* **2014**, *8*, e2986, doi:10.1371/journal.pntd.0002986.
3. Laskowski, R.A.; MacArthur, M.W.; Moss, D.S.; Thornton, J.M. PROCHECK: a program to check the stereochemical quality of protein structures. *Journal of Applied Crystallography* **1993**, *26*, 283-291, doi:10.1107/S0021889892009944.
4. Darzi, Y.; Letunic, I.; Bork, P.; Yamada, T. iPath3.0: interactive pathways explorer v3. *Nucleic Acids Research* **2018**, *46*, W510-W513, doi:10.1093/nar/gky299.
5. Jespersen, M.C.; Peters, B.; Nielsen, M.; Marcatili, P. BepiPred-2.0: improving sequence-based B-cell epitope prediction using conformational epitopes. *Nucleic Acids Research* **2017**, *45*, W24-W29, doi:10.1093/nar/gkx346.
